# Supplementary material for: Postpartum management of hypertensive disorders of pregnancy: a systematic review
Source: BMJ Open. 2017 Nov 28;7(11):e018696. doi: 10.1136/bmjopen-2017-018696 (PMC5719299; doi:10.1136/bmjopen-2017-018696)
Supplement: Supplementary file 1 [file bmjopen-2017-018696supp001.pdf]

Appendix S1: Management of hypertensive disorders of pregnancy in the postpartum period: A systematic review protocol

Registration: PROSPERO CRD42015015527

http://www.crd.york.ac.uk/PROSPERO/display_record.asp?ID=CRD42015015527#.VL4ZI9KsWCk

Authors: Alexandra E Cairns, Louise Pealing, Nia Roberts, Richard J McManus
Corresponding author: Alexandra E Cairns, Nuffield Department of Primary Care Health Sciences, University of Oxford, New Radcliffe House, Radcliffe Observatory Quarter, Woodstock Road, Oxford, OX2 6GG
alexandra.cairns@phc.ox.ac.uk

Amendments: Protocol first published 22/12/2014 (version 1.0). Protocol amended (version 2.0 25/03/2015) to include all reporting items from the PRISMA-P 2015 checklist, and PROSPERO registration number.

Review funder: NIHR Collaborations for Leadership in Applied Health Research and Care (funding A Cairns' fellowship)

Review sponsor: University of Oxford

Abstract

Rationale: Hypertensive disorders of pregnancy (gestational hypertension and pre-eclampsia) are a leading cause of direct maternal death in the UK, and affect approximately 5-10% of pregnancies. Hypertensive disorders of pregnancy persist during the postpartum period, and complications can occur during this time.

Research question: How should hypertensive disorders of pregnancy be managed in the postnatal period to minimise harm to patients and optimise quality of life?

Objectives:

1. Organisation of care: how should blood pressure be monitored in women with hypertensive disorders of pregnancy in the postnatal period?
2. What blood pressure thresholds should be used for anti-hypertensive treatment initiation, adjustment and cessation in the postnatal period?
3. Which anti-hypertensive medication(s) should be used in the postnatal period?
4. What are the benefits and harms of other therapeutic interventions for women with hypertensive disorders of pregnancy in the postnatal period?

Search strategy: Medline and nine other electronic databases will be searched for articles published from inception until October 2014 using a search strategy designed to capture all the relevant literature concerning the management of hypertensive disorders of pregnancy in the postnatal period.

Study eligibility criteria:
Population: postnatal women with gestational hypertension or pre-eclampsia as defined by study
Intervention: therapeutic intervention for hypertensive disorders of pregnancy
Comparisons: another intervention, placebo or no intervention
Study design: RCT, prospective or retrospective cohort study or case-control study
Publication date: no restrictions
Language: no restrictions

Data management and extraction: Two reviewers will first review the titles of articles yielded by the search, and then the abstracts of articles of potential relevance. The full papers of potentially eligible papers will be assessed, and data extracted independently by the two reviewers using a data extraction sheet. Differences in study selection and data extraction will be resolved by discussion.

Assessment of methodological quality: This will be done using the Cochrane Collaboration's tool for assessing risk of bias in randomised trials, and for the assessment of bias in cohort and case-control studies we will use the Newcastle-Ottawa quality assessment scales.

Systematic review registration: This systematic review is registered with PROSPERO (International prospective register of systematic reviews).

Rationale

Definitions

The National Institute for Health and Clinical Excellence (NICE) defines gestational hypertension as new-onset raised blood pressure (≥ 140/90mmHg) beyond 20 weeks gestation. NICE defines pre-eclampsia as new-onset raised blood pressure (≥ 140/90mmHg) together with new-onset significant proteinuria (≥ 300mg/24hr), beyond 20 weeks gestation (1).
The International Society for the Study of Hypertension in Pregnancy (ISSHP) defines pre-eclampsia as new-onset raised blood pressure (as defined by NICE) in association with one of new-onset significant proteinuria (as defined by NICE), maternal organ dysfunction or uteroplacental insufficiency (2).

Epidemiology

Hypertensive disorders of pregnancy remain the second commonest direct cause of maternal death in the USA (3). Until recently this has also been the case in the UK (CMACE 2006-8)(4), but the most recent Confidential Enquiry into maternal deaths showed that for the triennium 2009-11, pre-eclampsia and eclampsia was the fourth commonest cause of direct death (behind thrombosis, genital tract sepsis and haemorrhage), with a rate of 0.42 deaths per 100,000 maternities (5).

A recent population-based retrospective study in the United States found the rate of pre-eclampsia to be 3.4%. This study showed a slight, but significant increase, in the rates of both mild, and to a greater extent, severe pre-eclampsia over the period studied (1980-2010) (6).

Reviews of the literature, and national guidelines, quote rates of gestational hypertension between 6% (7) and 15% (8). A retrospective study using data from the National Hospital Discharge Survey in the United States (1987-2004) demonstrated an incidence of 30.6 cases of gestational hypertension per 1000 deliveries in 2003-2004 (3.1%) (9). In a well-designed large randomised controlled trial assessing preventative strategies for hypertensive disorders of pregnancy in low risk, nulliparous women the incidence of gestational hypertension across both groups was 6% (10).

Physiology of blood pressure in pregnancy and postpartum

As a result of a significant decrease in systemic vascular resistance (as early as 5 weeks gestation) (11) there is a decrease in arterial pressures from early in the first trimester. Arterial pressures reach a nadir in the second trimester, and then begin to rise in the third trimester, before reaching near-preconception levels in the postnatal period (12).

Figure 1: Serial blood pressures before, during and after pregnancy (reproduced from the data of Mahendru et al. 2014) (12)

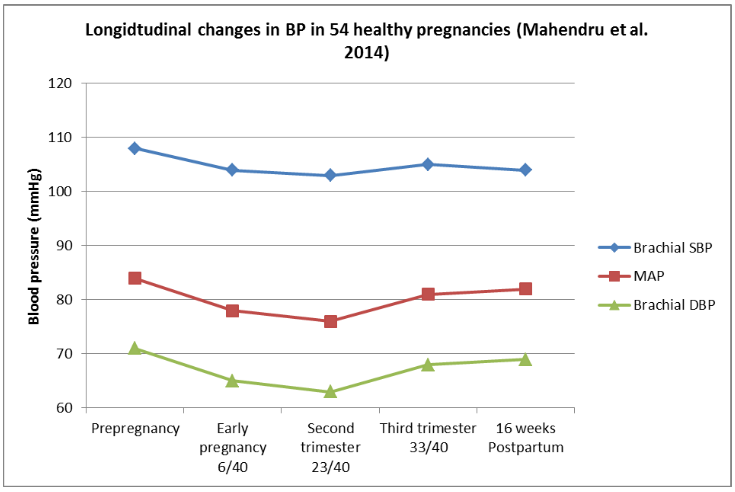

In gestational hypertension and pre-eclampsia the normal pregnancy-induced vasodilatation is reversed. In untreated women with pre-eclampsia significant increases in systemic vascular resistance are seen and result in elevation of blood pressure (13).

Hypertensive disorders of pregnancy in the postpartum period

There has been considerable focus on blood pressure control during pregnancy, especially with respect to pregnancy outcome. However, it is recognised that hypertensive disorders of pregnancy do persist during the postpartum period, and that complications can occur during this time. A small retrospective observational study published in 1987 looked at 67 women with moderate-severe pre-eclampsia: there was often an initial decrease in blood pressure after delivery, but this was followed by a rise to hypertensive levels in many women. In 50% of cases the blood pressure was 150/100mmHg or higher on day 5 after birth. The authors recommended continuing blood pressure monitoring and treatment in the postpartum period for women with a diagnosis of pre-eclampsia (14).

Most women with hypertensive disorders of pregnancy will be treatment-free by 3 months postpartum. In women whose blood pressure normalised after delivery the mean time to normalisation in a retrospective cohort study of 62 women was 5.4 weeks (15). This rapidly changing blood pressure, with shifting medication requirement, poses an additional challenge in terms of how best to manage this down-titration.

Approximately one third of eclamptic seizures occur postpartum, and studies suggest that over half of these seizures occur more than 48 hours after birth. Chames et al. (2002) highlight the importance of education of women and clinicians regarding prodromal symptoms of eclampsia in the postnatal

period (16). A case series published in 2005 of patients who sustained a stroke in association with severe pre-eclampsia or eclampsia, showed that more than half (57%) of these strokes occurred in the postpartum period (17).

Current guidelines

NICE guidelines highlight that very few clinical studies have addressed the management of blood pressure postpartum, and in practice clinical care is typically to continue antepartum antihypertensive medication and monitor blood pressure in the community with a focus on prevention of over-treatment.

NICE recommend frequency of monitoring in the postnatal period for both pre-eclampsia and gestational hypertension. The guidelines also stipulate thresholds for considering increasing or starting anti-hypertensive medication during this period (150/100 mmHg), and for reduction or stopping anti-hypertensive medication (consider at < 140/90 mmHg, and reduce at < 130/80 mmHg) (1).

Research question

How should hypertensive disorders of pregnancy be managed in the postnatal period to minimise harm to patients and optimise quality of life?

Objectives

The aim is to establish what evidence exists to guide the optimal approach to management of gestational hypertension and pre-eclampsia in the postnatal period. We want to address the specific sub-questions:

1. Organisation of care: how should blood pressure be monitored in women with hypertensive disorders of pregnancy in the postnatal period?
2. What blood pressure thresholds should be used for anti-hypertensive treatment initiation, adjustment and cessation in the postnatal period?
3. Which anti-hypertensive medication(s) should be used in the postnatal period?
4. What are the benefits and harms of other therapeutic interventions for women with hypertensive disorders of pregnancy in the postnatal period?

Information sources and search strategy

The systematic review of ‘management of hypertensive disorders of pregnancy in the postpartum period’ will be conducted in line with the PRISMA statement (18). Completion of a systematic review

is an iterative process, and it may be that modifications to the original review protocol are required during its conduct.

A search strategy designed to capture all the relevant literature concerning the management of hypertensive disorders of pregnancy in the postnatal period will be developed by an experienced trial search co-ordinator. Potentially relevant studies will be identified following screening of title and abstract of studies captured by the search and full text assessed for suitability.

Resources to be searched from inception to October 2014:

- Medline (Appendix 3) and 9 other electronic databases
- Trial registers (ClinicalTrials.gov; Current Controlled Trials; WHO; PROSPERO)
- Meta Search Engines
- Hand searches of reference lists
- Citation searching on Scopus and Web of Science
- Related articles search on PubMed
- Contact with authors and professional bodies / organisations: Experts in this field will be contacted for their recommendations of potentially relevant citations (19)

Study eligibility criteria

INCLUSION CRITERIA

Population: postnatal women with hypertensive disorders of pregnancy (gestational hypertension or pre-eclampsia).

Intervention: therapeutic intervention for management of hypertensive disorders of pregnancy

Comparisons: other intervention, placebo or no intervention

Study design: randomised controlled trial, cohort study (prospective and retrospective) or case-control study; human studies only

Publication Date: no restrictions

Language: no restrictions

EXCLUSION CRITERIA

Exclude report / study if **any** exclusion criteria fulfilled:

Population: antenatal or intrapartum women with hypertensive disorders of pregnancy; end-organ complications of pre-eclampsia (eclampsia, renal failure, HELLP syndrome)

Intervention: treatment of HELLP syndrome (haemolysis, elevated liver enzymes and low platelets); prevention or management of eclampsia; prevention of postpartum hypertension; choice of anaesthetic or sedative in pre-eclampsia; observational studies

Comparisons: no control group

Study design: guidelines, reviews, expert opinions, letters, commentaries, audits, case series and case reports excluded; animal studies

Data extraction

Two reviewers (AC and LP) will screen the titles and abstracts of articles yielded by the search against the eligibility criteria. Discrepancies will be resolved by consensus before determining the list of full papers for review. The reports will be screened independently by the two reviewers, and discrepancies will be resolved by discussion before deciding which papers to include in the review.
Data from included studies will be extracted independently by the two reviewers using a piloted and standardised data extraction sheet. Differences in data extraction will be resolved by discussion.
In the event that there is more than one report published about a single study: the reports will be reviewed separately but the data from that study grouped in our analysis, and the primary reference will be used.
In the event that data is missing from a report (for example the sole publication is a conference abstract) we will contact the authors directly to request further detail.
The study characteristics (study size, population, setting, study design, methodology, intervention, controls if applicable, outcome measures, and follow up period) will be recorded and reported.

Data synthesis

The data extracted will be aggregate.

Due to the heterogeneous nature of the outcomes reported in these studies a narrative synthesis is planned.

For trials where the population study is peripartum (i.e. a mixture of antepartum, intrapartum and postpartum) we will extract the data for the postpartum women and analyse this. If this is not feasible from the reported data then we will contact the study authors to request the data for this subgroup.

Outcomes

The results of all clinically relevant outcomes in hypertensive disorders of pregnancy that would be important to clinicians and patients will be extracted and reported.
The main outcomes we are interested in are listed in table 1 below:

Table 1

	Outcome measures	Timing
**Primary outcome(s)**	Maternal mortality<br>Major maternal morbidity (ischaemic stroke, intracranial haemorrhage, eclamptic seizure)<br>Systolic blood pressure control<br>Diastolic blood pressure control<br>Mean arterial pressure control	Direct maternal deaths upto day 42 postpartum; later maternal deaths upto 1 year postpartum
**Secondary outcome(s)**	Critical care admission<br>Postnatal readmission to secondary care<br>Length of hospital stay following delivery<br>Anti-hypertensive medication requirement<br>Maternal side effects of intervention<br>Development of pre-eclampsia with severe features<br>Postnatal complication requiring intervention<br>Urine output<br>Laboratory values	

Assessment of methodological quality

We will assess the risk of bias in each study. For randomised trials this will be done using the Cochrane Collaboration's tool for assessing risk of bias in randomised trials (Appendix 1, Table 2) (20). For each study the key domains will be identified, and then an overall assessment of bias within each trial made, according to the guidance published by the Cochrane Collaboration (Appendix 1, Table 2).

For the assessment of bias in cohort and case-control studies we will use the Newcastle-Ottawa quality assessment scales (Appendix 2, Tables 4 and 5) (21).

We will make a global assessment of bias across trials, based on the guidance from the Cochrane Collaboration (Appendix 1, Table 3):

- EITHER Most information is from trials at low risk of bias;
- OR most information is from trials at low or unclear risk of bias;
- OR the proportion of information from trials at high risk of bias is sufficient to affect the interpretation of results

Discussion

A Cochrane Review (2013) addresses the question of 'prevention and treatment of postpartum hypertension'. This only includes randomised controlled trials (9 in total), and does not address the issue of monitoring blood pressure during this period (22). Given the paucity of evidence cited in this area we believe there is a place for a review looking at all available evidence for the optimal approach to management of hypertensive disorders of pregnancy in the postpartum period.

Conflicts of interest

Neither AC nor LP have any conflicts of interest.

References

1. National Institute for Health and Care Excellence. NICE Clinical Guideline 107: Hypertension in pregnancy: the management of hypertensive disorders during pregnancy. 2011.
2. Tranquilli AL, Dekker G, Magee L, Roberts J, Sibai BM, Steyn W, et al. The classification, diagnosis and management of the hypertensive disorders of pregnancy: A revised statement from the ISSHP. Pregnancy Hypertension: An International Journal of Women's Cardiovascular Health. 2014;4(2):97-104.
3. American College of Obstetricians and Gynecologists. Diagnosis and management of preeclampsia and eclampsia. ACOG Practice Bulletin. ACOG Practice Bulletin No. 33: 2002.
4. Cantwell R, Clutton-Brock T, Cooper G, Dawson A, Drife J, Garrod D, et al. Saving Mothers' Lives: Reviewing maternal deaths to make motherhood safer: 2006-2008. The Eighth Report of the Confidential Enquiries into Maternal Deaths in the United Kingdom. BJOG : an international journal of obstetrics and gynaecology. 2011;118 Suppl 1:1-203.
5. Knight M, Kenyon S, Brocklehurst P, Neilson J, Shakespeare J, Kurinczuk J. Saving Lives, Improving Mothers' Care (MBRRACE-UK). 2014.
6. Ananth CV, Keyes KM, Wapner RJ. Pre-eclampsia rates in the United States, 1980-2010: age-period-cohort analysis. Bmj. 2013;347:f6564.
7. Yoder SR, Thornburg LL, Bisognano JD. Hypertension in pregnancy and women of childbearing age. The American journal of medicine. 2009;122(10):890-5.
8. National Institute for Health and Care Excellence. NICE Clinical Guideline 107: Hypertension in pregnancy: Costing report. Implementing NICE guidance. 2010.
9. Wallis AB, Saftlas AF, Hsia J, Atrash HK. Secular trends in the rates of preeclampsia, eclampsia, and gestational hypertension, United States, 1987-2004. American journal of hypertension. 2008;21(5):521-6.
10. Sibai BM, Caritis SN, Thom E, Klebanoff M, McNellis D, Rocco L, et al. Prevention of preeclampsia with low-dose aspirin in healthy, nulliparous pregnant women. The National Institute of Child Health and Human Development Network of Maternal-Fetal Medicine Units. The New England journal of medicine. 1993;329(17):1213-8.
11. Chapman AB, Abraham WT, Zamudio S, Coffin C, Merouani A, Young D, et al. Temporal relationships between hormonal and hemodynamic changes in early human pregnancy. Kidney international. 1998;54(6):2056-63.
12. Mahendru AA, Everett TR, Wilkinson IB, Lees CC, McEniery CM. A longitudinal study of maternal cardiovascular function from preconception to the postpartum period. Journal of hypertension. 2014;32(4):849-56.
13. Visser W, Wallenburg HC. Central hemodynamic observations in untreated preeclamptic patients. Hypertension. 1991;17(6 Pt 2):1072-7.
14. Walters BN, Walters T. Hypertension in the puerperium. Lancet. 1987;2(8554):330.
15. Podymow T, August P. Postpartum course of gestational hypertension and preeclampsia. Hypertension in pregnancy. 2010;29(3):294-300.
16. Chames MC, Livingston JC, Ivester TS, Barton JR, Sibai BM. Late postpartum eclampsia: a preventable disease? American journal of obstetrics and gynecology. 2002;186(6):1174-7.
17. Martin JN, Jr., Thigpen BD, Moore RC, Rose CH, Cushman J, May W. Stroke and severe preeclampsia and eclampsia: a paradigm shift focusing on systolic blood pressure. Obstetrics and gynecology. 2005;105(2):246-54.
18. Moher D, Liberati A, Tetzlaff J, Altman DG, Group P. Preferred reporting items for systematic reviews and meta-analyses: the PRISMA statement. PLoS medicine. 2009;6(7):e1000097.
19. McManus RJ, Wilson S, Delaney BC, Fitzmaurice DA, Hyde CJ, Tobias RS, et al. Review of the usefulness of contacting other experts when conducting a literature search for systematic reviews. Bmj. 1998;317(7172):1562-3.

20. Higgins JPT, Altman DG, Gøtzsche PC, Jüni P, Moher D, Oxman AD, et al. The Cochrane Collaboration's tool for assessing risk of bias in randomised trials2011 2011-10-18 10:55:48.
21. Wells GA, Shea B, O'Connell D, J. P, V. W, M. L, et al. The Newcastle-Ottawa Scale (NOS) for assessing the quality of nonrandomised studies in meta-analyses.
22. Magee L, von Dadelszen P. Prevention and treatment of postpartum hypertension. The Cochrane database of systematic reviews. 2013;4:CD004351.

Appendix 1

Table 2: Cochrane Collaboration's tool for assessing risk of bias (*adapted from Higgins and Altman*)(20)

**Bias domain**	**Source of bias**	**Support for judgment**	**Review authors' judgment** (assess as low, unclear or high risk of bias)
**Selection bias**	Random sequence generation	Describe the method used to generate the allocation sequence in sufficient detail to allow an assessment of whether it should produce comparable groups	Selection bias (biased allocation to interventions) due to inadequate generation of a randomised sequence
	Allocation concealment	Describe the method used to conceal the allocation sequence in sufficient detail to determine whether intervention allocations could have been foreseen before or during enrolment	Selection bias (biased allocation to interventions) due to inadequate concealment of allocations before assignment
**Performance bias**	Blinding of participants and personnel*	Describe all measures used, if any, to blind trial participants and researchers from knowledge of which intervention a participant received. Provide any information relating to whether the intended blinding was effective	Performance bias due to knowledge of the allocated interventions by participants and personnel during the study
**Detection bias**	Blinding of outcome assessment*	Describe all measures used, if any, to blind outcome assessment from knowledge of which intervention a participant received. Provide any information relating to whether the intended blinding was effective	Detection bias due to knowledge of the allocated interventions by outcome assessment
**Attrition bias**	Incomplete outcome data*	Describe the completeness of outcome data for each main outcome, including attrition and exclusions from the analysis. State whether attrition and exclusions were reported, the numbers in each intervention group (compared with total randomised participants), reasons for attrition or exclusions where reported, and any re-inclusions in analyses for the review	Attrition bias due to amount, nature, or handling of incomplete outcome data
**Reporting bias**	Selective reporting	State how selective outcome reporting was examined and what	Reporting bias due to selective

		was found	outcome reporting
**Other bias**	Anything else, ideally Pre-specified	State any important concerns about bias not covered in the other domains in the tool	Bias due to problems not covered elsewhere

*Assessments should be made for each main outcome or class of outcomes.

Table 3: Approach to formulating summary assessments of risk of bias for each important outcome (across domains) within and across trials (*adapted from Higgins and Altman*)(20)

Risk of bias	Interpretation	Within a trial	Across trials
**Low risk of bias**	Bias, if present, is unlikely to alter the results seriously	Low risk of bias for all key domains	Most information is from trials at low risk of bias
**Unclear risk of bias**	A risk of bias that raises some doubt about the results	Low or unclear risk of bias for all key domains	Most information is from trials at low or unclear risk of bias
**High risk of bias**	Bias may alter the results seriously	High risk of bias for one or more key domains	The proportion of information from trials at high risk of bias is sufficient to affect the interpretation of results

Appendix 2

Table 4: Newcastle-Ottawa quality assessment scale case control studies(21)

A study can be awarded a maximum of one star for each numbered item within the Selection and Exposure categories. A maximum of two stars can be given for Comparability.

**Selection**	Is the case definition adequate?	a) Yes, with independent validation ✵<br>b) Yes, e.g. record linkage or based on self-reports<br>c) No description
	Representativeness of the cases	a) Consecutive or obviously representative series of cases ✵<br>b) Potential for selection biases not stated
	Selection of controls	a) Community controls ✵<br>b) Hospital controls<br>c) No description
	Definition of controls	a) No history of disease (endpoint) ✵<br>b) No description of source
**Comparability**	Comparability of cases and controls on the basis of the design or analysis	a) Study controls for <<_>> (select the post important factor) ✵<br>b) Study controls for any additional factor ✵
**Exposure**	Ascertainment of exposure	a) Secure records (e.g. surgical records) ✵<br>b) Structured interview where blind to case/control status ✵<br>c) Interview not blinded to case/control status<br>d) Written self-report or medical record only<br>e) No description
	Same method of ascertainment for cases and controls	a) Yes ✵<br>b) No
	Non-response rate	a) Same rate for both groups ✵<br>b) Non-respondents described<br>c) Rate different and no designation

Table 5: Newcastle-Ottawa quality assessment scale cohort studies(21)

A study can be awarded a maximum of one star for each numbered item within the Selection and Outcome categories. A maximum of two stars can be given for Comparability.

**Selection**	Representativeness of the exposed cohort	a) Truly representative of the average <<_>> (describe) in the community ✵<br>b) Somewhat representative of the average <<_>> (describe) in the community □<br>c) Selected group of users e.g. nurses, volunteers<br>d) No description of the derivation of the cohort
	Selection of the non-exposed cohort	a) Drawn from the same community as the exposed cohort ✵<br>b) Drawn from a different source<br>c) No description of the derivation of the non-exposed cohort
	Ascertainment of exposure	a) Secure record (e.g. surgical records) ✵<br>b) Structured interview ✵<br>c) Written self-report<br>d) No description

	Demonstration that the outcome of interest was not present at start of study	a) Yes ✹<br>b) No
**Comparability**	Comparability of cases and controls on the basis of the design or analysis	a) Study controls for <<_>> (select the post important factor) ✹<br>b) Study controls for any additional factor ✹
**Outcome**	Assessment of outcome	a) Independent blind assessment ✹<br>b) Record linkage ✹<br>c) Self-report<br>d) No description
	Was follow-up long enough for outcomes to occur	a) Yes (select an adequate follow up period for outcome of interest) ✹<br>b) No
	Adequacy of follow-up of cohorts	a) Complete follow-up – all subjects accounted for ✹<br>b) Subjects lost to follow-up unlikely to introduce bias: >_ _ % (select an adequate %) follow-up rate, or description provided of those lost) ✹<br>c) Follow-up rate < _ _ % (select an adequate %) and no description of those lost<br>d) No statement

Appendix 3: Medline search strategy

# ▼	Searches	Results
1	Pregnancy/ and Hypertension/	9226
2	exp Hypertension, Pregnancy-Induced/	29022
3	((pregnan* or gestation* or maternal or prenatal or pre-natal or antenatal or ante-natal or antepart* or ante-part* or obstetric*) and (hypertens* or blood pressure or bp or dbp or sbp or diastolic or systolic)).ti.	6787
4	((pregnan* or gestation* or maternal or prenatal or pre-natal or antenatal or ante-natal or antepart* or ante-part* or obstetric*) adj3 (hypertens* or blood pressure or bp or dbp or sbp or diastolic or systolic)).ti,ab.	12434
5	(eclamp* or preeclamp* or pre-eclamp* or hellp).ti,ab.	25194
6	1 or 2 or 3 or 4 or 5	46611
7	Postnatal Care/	4044
8	Aftercare/	6684
9	Postpartum Period/ and Maternal Health Services/	126
10	exp Puerperal Disorders/ and Maternal Health Services/	196
11	Postpartum period/ and (exp Antihypertensive agents/ or exp calcium channel blockers/ or exp Angiotensin-Converting Enzyme Inhibitors/ or exp Adrenergic beta-Antagonists/ or exp Diuretics/)	187
12	exp Puerperal disorders/ and (exp Antihypertensive agents/ or exp calcium channel blockers/ or exp Angiotensin-Converting Enzyme Inhibitors/ or exp Adrenergic beta-Antagonists/ or exp Diuretics/)	237
13	Postpartum period/ and exp Curettage/	30
14	exp Puerperal disorders/ and exp Curettage/	118
15	Postpartum period/ and hypertension/dt, th	33
16	exp Puerperal disorders/ and hypertension/dt, th	54
17	exp Puerperal disorders/dt, th	6408
18	((postnatal or post-natal or postpart* or post-part* or puerper*) adj5 (care or healthcare or service* or program* or scheme* or intervention*)).ti,ab.	4407
19	((postnatal or post-natal or postpart* or post-part* or puerper*) adj5 (clinic? or unit? or visit* or referral? or appointment?)).ti,ab.	1491
20	((postnatal or post-natal or postpart* or post-part* or puerper*) adj5 (manage* or treat* or therap* or medication? or recovery)).ti,ab.	7287
21	((postnatal or post-natal or postpart* or post-part* or puerper*) adj5 (antihypertens* or anti-hypertens* or calcium channel block* or beta block* or b block* or ace inhibitor* or angiotensin converting enzyme inhibitor* or diuretic*)).ti,ab.	41
22	((postnatal or post-natal or postpart* or post-part* or puerper*) adj5 (evaluat* or assess* or screen* or diagnos* or monitor* or follow up or supervis*)).ti,ab.	7562
23	((postnatal or post-natal or postpart* or post-part* or puerper*) adj5 curet*).ti,ab.	82
24	(postnatal or post-natal or postpart* or post-part* or puerper*).ti.	41491
25	7 or 8 or 9 or 10 or 11 or 12 or 13 or 14 or 15 or 16 or 17 or 18 or 19 or 20 or 21 or 22 or 23 or 24	64775

26	6 and 25	1896
27	((postnatal or post-natal or postpart* or post-part* or puerper*) and (hypertens* or blood pressure)).ti.	270
28	26 or 27	1990
29	exp animals/ not humans.sh.	4079856
30	(rat or rats or rodent? or mice or mouse or cow or cows or cattle or calf or calves or ewe? or sheep or goat or ruminant? or pig or pigs or minipig? or chicken? or horse or horses or murine or bovine or ovine or porcine or animal?).ti.	1682619
31	29 or 30	4373527
32	28 not 31	1881
