# Supplementary material for: Postpartum management of hypertensive disorders of pregnancy: a systematic review
Source: BMJ Open. 2017 Nov 28;7(11):e018696. doi: 10.1136/bmjopen-2017-018696 (PMC5719299; doi:10.1136/bmjopen-2017-018696)
Supplement: Supplementary file 3 [file bmjopen-2017-018696supp003.pdf]

Appendix S3: Primary reasons for article exclusion (n = 35)

	Population not postnatal	Intervention not targeted at management of BP	No control group	Study design
**n**	4	6	22	3
**Study IDs**	Berks 2015<br>Gerard 1983<br>Scardo 1999<br>Wacker 2006	Chandrasekaran 2015<br>Ehrenberg 2004<br>Ehrenberg 2006<br>Ossada 2016<br>Wasden 2012<br>Younger-Lewis 2016	Al Waili 2004<br>Alicino 1962<br>Barton 1991<br>Belfort 1988<br>Belfort 1992<br>Bittle 2014<br>Bosio 2003<br>Correa 1982<br>Dulitzky 1987<br>Hirshberg 2016<br>Hirshberg 2017<br>Hunter 1961<br>Onishi 2015<br>Robinson 1964<br>Rodriguez 2012<br>Saghir<br>Smith 2005<br>Sukerman-Voldman 1985<br>Taslimi 1991<br>Tkacheva 2006<br>Wacker 1994<br>Walters 1984	Editor, Emergency Medicine 1990<br>Cursino 2015<br>Gallegos 1961
