# Supplementary material for: Postpartum management of hypertensive disorders of pregnancy: a systematic review
Source: BMJ Open. 2017 Nov 28;7(11):e018696. doi: 10.1136/bmjopen-2017-018696 (PMC5719299; doi:10.1136/bmjopen-2017-018696)
Supplement: Supplementary file 4 [file bmjopen-2017-018696supp004.pdf]

APPENDIX S4: Main characteristics of included studies (n=39)

Author and year	Methods		Participants				Intervention		Outcomes	
	Study design	*Duration*	*n*[*]	*Age /y*[†]	*Setting*	*Country*	*Intervention*	*Control(s)*	*Primary*	*Secondary*
**ANTIHYPERTENSIVE MEDICATIONS (18 studies)**										
**Calcium channel blockers**										
**Barton 1990**[32]	RCT	Enrolled immediately after birth<br>F/U 48h	31	24.0<br>26.3	Tertiary referral hospital	USA	Nifedipine 10mg PO 4-hourly for 48 hours	Placebo	MAP	SBP, DBP<br>Maternal heart rate<br>AHT requirement<br>Urine output<br>Laboratory values (urine protein, creatinine clearance, HCT, plt, ALT, BUN, creatinine, serum electrolytes, UA, urine specific gravity)
Vermillion 1999[21]	RCT	Enrolled within 24h of birth<br>F/U 3 – 24h	21	27.2±7.3<br>27.0±6.4	Tertiary referral hospital	USA	Nifedipine 10mg stat PO then 20mg every 20min until BP <160/110mmHg or max 5 doses + IV placebo	Labetalol 20mg, then 40mg, then 80mg IV every 20min until BP <160/110mmHg or max 5 doses (300mg) + PO placebo	SBP + DBP	SBP (failure to achieve target <160mmHg)<br>DBP (failure to achieve target <110mmHg)<br>Maternal side effects<br>AHT requirement<br>Urine output
**Sayin 2005**[34]	RCT	Enrolled 24h after birth<br>F/U 72h after BP controlled	83	17-41	Tertiary referral hospital	Turkey[‡]	Nifedipine 10mg PO QDS until BP <150/100mmHg for 48h	Methyldopa 250mg PO TDS	SBP + DBP	Maternal mortality<br>AHT requirement<br>Hypertensive retinopathy
**Vasodilators**										
Palot 1979[36]	Retrospective cohort study	Not specified	54	24.5 (17-37)	Not specified	France[†]	Hydralazine 5mg IV stat then 1% IV infusion, furosemide 20mg IV stat and 30% hypertonic glucose	(1) Clonidine IV and furosemide 20mg IV stat<br>*Or* (2) Non-systematic treatment	Maternal morbidity (development of pre-eclampsia with severe features)	BP (time to resolution of hypertension)

Abbreviations: AHT = antihypertensive; ALT = alanine aminotransferase; BD = twice daily; BP = blood pressure; BUN = blood urea nitrogen; DBP = diastolic blood pressure; F/U = follow-up; IM = intramuscular; IV = intravenous; MAP = mean arterial pressure; plt = platelets; PN = postnatal; OD = once daily; PO = oral; QDS = four times daily; RCT = randomised controlled trial; SBP = systolic blood pressure; S/L = sublingual; TDS = three times daily; UA = uric acid

[*] n = postnatal population (antenatal excluded)

[†] Where separate data available for study groups, intervention group listed first

[‡] Non-English language manuscript

Author and year	Methods		Participants				Intervention		Outcomes	
	Study design	*Duration*	*n*[*]	*Age /y*[†]	*Setting*	*Country*	*Intervention*	*Control(s)*	*Primary*	*Secondary*
**Griffis 1989**[38,39]	RCT	F/U 24h	26	Not specified	Tertiary referral hospital	USA	Hydralazine 20mg IM QDS for 24h	Methyldopa 250mg IV QDS x 24h	MAP	Maternal side effects<br>AHT requirement<br>Urine output (time to diuresis)
**Walss Rodriguez 1991**[40]	RCT	Not specified	38	16-40	Not specified	Mexico[†]	Hydralazine 40mg PO QDS, duration not specified; if DBP >110mmHg PRN nifedipine 10mg SL every 30min, to max 3 doses	Nifedipine 10mg SL every 30min if DBP ≥110mmHg	SBP	DBP<br>AHT requirement
Begum 2002[17]	Quasi-randomised trial	Not specified	15	24.09±4.93<br>22.72±5.08	Tertiary referral hospital	Bangladesh	Hydralazine 5mg then 2mg IV bolus every 15min until DBP 90-95mmHg	Hydralazine 20mg/200ml normal saline IV infusion; 10 drops per min, increased by 5 drops at 15min intervals; until DBP 90-95mmHg	DBP	Maternal side effects<br>AHT requirement<br>Maternal heart rate
**Vigil-De Gracia 2007**[35]	RCT	Enrolled day 2-3 after birth<br>F/U not specified	82	29.9±5.9<br>31.3±5.5	Tertiary referral hospital	Panama	Hydralazine 5mg IV every 20min until BP <160/110mmHg or max 5 doses	Labetalol 20mg, then 40mg, then 80mg IV every 20min until BP <160/110mmHg or max 5 doses (300mg)	SBP + DBP	Maternal mortality<br>Maternal morbidity (development of pre-eclampsia with severe features)<br>Maternal side effects<br>AHT requirement<br>Maternal heart rate
Hennessy 2007[23]	RCT	F/U 3h	37	21-43<br>(mean 33)	Tertiary referral hospital	Australia	Diazoxide 15mg IV every min, maximum dose 300mg	Hydralazine 5mg IV every 2min, maximum 15mg	SBP + DBP	SBP (10mmHg above target after 1 hour)<br>DBP (10mmHg above target after 1 hour)<br>Maternal side effects (including hypotension)<br>Time taken to administer drug
**Beta blockers**										
Garden 1982[24]	RCT	Enrolled immediately after birth<br>F/U 45-64h	6	25-44<br>20-28	Tertiary referral hospital	South Africa	Labetalol 200mg/200ml 5% dextrose, 20mg/h IV infusion, doubled every 30min until DBP <100mmHg or maximum dose 160mg/h	Dihydralazine 100mg/200ml 5% dextrose, 10mg/h IV infusion, doubled every 30min until DBP <100mmHg or maximum dose 80mg/h	DBP	Maternal side effects
**Fidler 1982**[42]	RCT	Enrolled 4 days after birth<br>F/U 9 days	80	29.7±1.0<br>27.8±0.9	Tertiary referral hospital	UK	Timolol 5mg PO TDS for 9 days	Methyldopa 250mg PO TDS for 9 days	DBP	SBP<br>DBP (time to achieve control, proportion achieving control)<br>Maternal side effects

Author and year	Methods		Participants				Intervention		Outcomes	
	Study design	*Duration*	*n**	*Age /y*†	*Setting*	*Country*	*Intervention*	*Control(s)*	*Primary*	*Secondary*
Mabie 1987[22]	RCT	Enrolled 1-96 hours after birth<br>F/U 3h	41	23.7±6.9<br>22.9±7.0	Tertiary referral hospital	USA	Labetalol 20mg IV every 10min then escalating until DBP <100mmHg or maximum cumulative dose reached (300mg)	Hydralazine 5mg IV every 10minuntil DBP <100mmHg	MAP	MAP (time to maximal decrease)<br>DBP (achieving target <100mmHg)<br>Maternal side effects<br>AHT requirement<br>Maternal heart rate
Shumard 2016[41]	Retrospective cohort study	F/U not specified (but >24h)	128	Not specified	Not specified	USA	Labetalol PO (variable dose and frequency)	Nifedipine PO (variable dose and frequency)	Length of hospital stay after birth	SBP, DBP<br>AHT requirement
Sharma 2017[27 28]	RCT	F/U not specified (but >24h)	50	Not specified	Tertiary referral hospital	USA	Labetalol 200mg PO BD	Nifedipine XL 30mg PO OD	SBP + DBP	Maternal side-effects<br>Length of PN hospital stay<br>AHT requirement
**Other**										
Gaisin 2013[25]	RCT	6 months	30	23-29	Not specified (hospital)	Russia	Indapamide 1.5mg PO OD, duration unclear	Adjusted dose methyldopa	SBP + DBP	Safety data<br>Laboratory values (lipid and glucose metabolism)<br>Adherence to treatment<br>Weight reduction<br>Decrease in albuminuria<br>Decrease in LV mass index<br>Endothelial function<br>Milk production
Gaisin 2014[37]	RCT	1 year	30	24-28	Not specified (hospital)	Russia	Indapamide 1.5mg PO OD with ursodeoxycholic acid 250mg PO TDS, duration unclear	Adjusted dose methyldopa	SBP + DBP	Maternal side effects<br>Laboratory values (atherogenic lipid profile, glucose metabolism. renal function)<br>Offspring adverse events<br>Weight reduction<br>Decrease in microalbuminuria<br>Decrease in LV mass index<br>Endothelial function
Krebs 1956[43 44]	Retrospective cohort study	F/U not specified (but >24h)	140	Not specified	Not specified	Switzer-land†	Reserpine 0.25mg PO or IM TDS or QDS for 7 days	Phenobarbital	SBP + DBP	SBP + DBP (non-responders)<br>Maternal side effects<br>Resolution of albuminuria<br>Resolution of oedema
Katz 2015[29-31]	RCT	F/U not specified	90	Not specified	Tertiary referral hospital	Brazil	Clonidine 0.1mg PO repeated every 20min to maximum 6 doses	Captopril 25mg PO repeated every 20min to maximum 6 doses	SBP + DBP	SBP (% reduction)<br>SBP + DBP (daily mean)<br>Maternal side effects<br>AHT requirement
**LOOP DIURETICS (3 studies)**										
**Matthews 1997[46]**	RCT	Enrolled 12-24h after birth<br>F/U 6 weeks	19	Not specified	Tertiary referral hospital	UK	Furosemide 40mg PO OD for 7 days	Placebo	MAP	Length of PN hospital stay<br>AHT requirement<br>Urine output<br>Laboratory values (hypokalemia)

Author and year	Methods		Participants				Intervention		Outcomes	
	Study design	*Duration*	*n*[*]	*Age /y*[†]	*Setting*	*Country*	*Intervention*	*Control(s)*	*Primary*	*Secondary*
**Ascarelli 2005**[16]	RCT	Enrolled 2-24h after birth<br>F/U 6 weeks	264	22.8±6.1<br>22.9±6.0	Tertiary referral hospital	USA	Furosemide 20mg PO OD + potassium 20mEq PO OD for 5 days	No intervention	SBP	Maternal morbidity (postnatal complication requiring intervention)<br>DBP<br>Length of PN hospital stay<br>AHT requirement<br>Maternal weight<br>Maternal HR<br>Duration of magnesium sulphate
Amorim 2015[45]	RCT	Enrolled immediately after birth<br>F/U 5 days	120	Not specified	Tertiary referral hospital	Brazil	Furosemide 40mg PO OD, duration not specified	Placebo	SBP + DBP	MAP<br>SBP (daily episodes ≥180mmHg)<br>DBP (daily episodes ≥110mmHg)<br>Length of PN hospital stay<br>AHT requirement<br>Urine output<br>Maternal heart rate
Veena 2017[19]	RCT	Enrolled <24h after birth	100	24.34±4.31<br>24.02±4.27	Tertiary referral hospital	India	Furosemide 20mg PO OD + nifedipine 10mg PO TDS for 3 days	Nifedipine 10mg PO TDS for 3 days	SBP + DBP	MAP<br>Maternal morbidity (postnatal complication requiring intervention)<br>Length of hospital stay after birth<br>Antihypertensive medication requirement<br>Urine output
**OTHER DRUGS (7 studies)**										
**Selective 5-HT antagonists**										
Weiner 1982[48]	RCT (crossover)	F/U not specified	5	Not specified	Tertiary referral	USA	R41468 IV (dose not specified) bolus then infusion for 90min	Placebo	MAP	MAP (rate at which hypertension returned post-infusion)<br>Urine output (infusion related diuresis)
Weiner 1984[49]	RCT (crossover)	Enrolled immediately after birth<br>F/U 3.5h	20	28±6.4	Tertiary referral	USA	Ketanserin 10mg IV bolus then 4mg/hr IV infusion. Repeat bolus after 5min if no response.	Placebo	SBP + DBP	Maternal side effects<br>DBP (target <95mmHg)<br>MAP<br>AHT requirement<br>Response rate
Montenegro 1985[50]	RCT (crossover)	Enrolled immediately after birth<br>F/U not specified	30	21.5 (13-31)	Tertiary referral hospital	USA	Ketanserin 10mg IV bolus, repeated if no response. If no response to second bolus IV infusion 4mg/hr (increments of 2mg/hr every 10min to max 12mg/hr).	Placebo	MAP	Maternal side effects

Author and year	Methods		Participants				Intervention		Outcomes	
	Study design	*Duration*	*n*[*]	*Age /y*[†]	*Setting*	*Country*	*Intervention*	*Control(s)*	*Primary*	*Secondary*
**Alternative therapies**										
**Hladunewich 2006**[51]	RCT	Enrolled immediately after birth<br>F/U 10 days	45	29±6<br>28±7	Tertiary referral hospital	USA	L-arginine 3.5g PO QDS or 10g IV TDS for 3-9 days	Placebo	MAP	Maternal side effects<br>SBP, DBP<br>AHT requirement<br>Laboratory values (glomerular filtration rate (GFR) (inulin clearance), Albumin/creatinine (A/C) ratio, vasoactive hormones (NO and cGMP), liver function tests (LFTs), plt)<br>Renal plasma flow (para-amino hippurate clearance), renal blood flow = renal plasma flow / (1-HCT), renovascular resistance
Liu 2009[52]	Quasi-randomised trial	Enrolled day 2 after birth<br>F/U 3 weeks	72	26.6±3.7<br>25.7±3.9	District general hospital	China[†]	Shengkangbao 10g PO or IV BD for 3 weeks	No intervention	Percentage of cases with positive albuminuria	SBP, DBP<br>Laboratory values (24h urinary albumin, plasma total protein, plasma albumin, urinary albumin negative inversion rate, renal function)
**Steroids**										
Barrilleaux 2005[53 54]	RCT	Enrolled immediately after birth<br>F/U 4.5 days	157 (175)	24.5±6.8<br>23.9±6.4	Tertiary referral hospital	USA	Dexamethasone 10mg x 2, then 5mg x 2 IV BD for 48 hours	Placebo	Antihypertensive medication requirement	MAP<br>Critical care admission<br>Length of PN hospital stay<br>Urine output<br>Laboratory values (plt, lactate dehydrogenase (LDH), aspartate aminotransferase (AST))<br>Stay in recovery >24h
**Atrial natriuretic peptide**										
Shigemitsu 2015[47]	Retrospective cohort study	F/U not specified	16	Not specified	Tertiary referral hospital	Japan	Carperitide (no further details)	Standard care	MAP	Maternal mortality<br>Maternal side effects<br>Need for dialysis<br>Time to diuresis
**UTERINE CURETTAGE (8 studies)**										
Salvatore 1967[58]	Prospective cohort study	Enrolled immediately after birth<br>F/U 10 days	48	16-45	Tertiary referral hospital	Brazil[††]	Uterine curettage	No intervention	SBP + DBP	Maternal morbidity (development of pre-eclampsia with severe features – seizures)
Magann 1993[59]	RCT	Enrolled immediately after birth<br>F/U 24h (telephone at 7 weeks)	32	22.9±5.6<br>23.4±6.6	Tertiary referral hospital	USA	Uterine curettage	No intervention	MAP	Maternal side effects<br>Length of PN hospital stay<br>AHT requirement<br>Urine output<br>Laboratory values (HCT, plt, AST, LDH)
Magann 1994[60]	RCT	Enrolled immediately after birth<br>F/U 48h (telephone at 7 weeks)	45	22.3±6.4<br>22.8±6.6<br>22.8±6.1	Tertiary referral hospital	USA	Uterine curettage	(1) Nifedipine PO<br>OR<br>(2) Usual care	MAP	Maternal side effects<br>Urine output<br>Laboratory values (HCT, plt, AST, LDH)

Author and year	Methods		Participants				Intervention		Outcomes	
	Study design	*Duration*	*n*[*]	*Age /y*[†]	*Setting*	*Country*	*Intervention*	*Control(s)*	*Primary*	*Secondary*
Gocmen 1996[57]	Prospective cohort study	Enrolled immediately after birth F/U 24h	50	Not specified	Tertiary referral hospital	Turkey[†]	Uterine curettage	No intervention	MAP	Urine output<br>Laboratory values (plt)
Gomez 2005[61]	RCT	Enrolled immediately after birth F/U not specified	86	Not specified	Tertiary referral hospital	Peru	Uterine curettage	No intervention	MAP	Maternal side effects<br>Length of PN hospital stay<br>AHT requirement<br>Urine output
Alkan 2006[62]	RCT	Enrolled immediately after birth F/U 24h	56	22.8±3.4<br>24.6±7.5	Tertiary referral hospital	Turkey	Uterine curettage	No intervention	MAP	Maternal side effects<br>Urine output<br>Laboratory values (plt, LDH, AST, ALT)
Ragab 2013[15]	RCT	Enrolled immediately after birth F/U 96h	420	Not specified	Tertiary referral hospital	Egypt	Uterine curettage	No intervention	MAP	Maternal mortality<br>Maternal morbidity (development of pre-eclampsia with severe features)<br>MAP (time to MAP ≤105mmHg)<br>Urine output<br>Laboratory values (creatinine, plt, UA)
Mallapur 2015[18]	RCT	Enrolled immediately after birth F/U 7 days	100	Not specified	Tertiary referral hospital	India	Uterine curettage	No intervention	MAP	Length of PN hospital stay<br>Urine output<br>Laboratory values (plt, renal and liver function)
**ORGANISATION OF CARE (2 studies)**										
York 1997[26]	RCT	Enrolled immediately after birth F/U 8 weeks	96[§]	28±7<br>27±7	Tertiary referral	USA	Contact with nurse specialist; early discharge if criteria met; 2 scheduled home visits and 10 telephone calls (twice weekly for 2 weeks, then weekly for 6 weeks) during F/U	Standard care	Postnatal readmission to secondary care	Functional status<br>Patient satisfaction<br>Neonatal rehospitalisation / acute neonatal care<br>Cost
Bibbo 2014[33]	Retrospective cohort study	F/U not specified (but >7 days)	138	Not specified	Tertiary referral hospital	USA	Specialised postpartum clinic	Usual care	Postnatal readmission to secondary care and triage visits	Primary care provider F/U

[§] Mixture of hypertension and diabetes – unable to separate
