# Supplementary material for: Postpartum management of hypertensive disorders of pregnancy: a systematic review
Source: BMJ Open. 2017 Nov 28;7(11):e018696. doi: 10.1136/bmjopen-2017-018696 (PMC5719299; doi:10.1136/bmjopen-2017-018696)
Supplement: Supplementary file 5 [file bmjopen-2017-018696supp005.pdf]

APPENDIX S5: Summary of main results for included studies (n=39)

ANTIHYPERTENSIVE MEDICATIONS (18 studies)

Calcium channel blockers

BARTON 1990[32]

Population: Postnatal women with severe pre-eclampsia
Setting: Tertiary referral centres, USA
Intervention: Nifedipine 10mg PO 4-hourly for 48 hours
Comparison: Placebo

Primary outcome	Treatment effect	Number of participants	Quality of the evidence
MAP (18-24 hours after birth)	Nifedipine group 93.9±1.6mmHg, placebo group 100.2±2.6mmHg. Difference -6.3mmHg (p<0.05).	31 (16 intervention, 15 control); follow-up complete for all participants	Double-blind RCT; overall low risk of bias

VERMILLION 1999[21]

Population: Antenatal and postnatal women with severe pre-eclampsia or super-imposed pre-eclampsia
Setting: Tertiary referral centres (USA)
Intervention: Nifedipine 10mg stat PO then 20mg every 20 minutes until BP <160/110mmHg or max 5 doses (90mg) + IV placebo
Comparison: Labetalol 20mg, then 40mg, then 80mg IV every 20 minutes until BP <160/110mmHg or max 5 doses (300mg) + PO placebo

Primary outcome	Treatment effect	Number of participants	Quality of the evidence
SBP + DBP (time to target <160/100mmHg)	Nifedipine group 25.1±13.6 minutes, labetalol group 43.6±25.4 minutes. Difference -18.5 minutes (p=0.002).	50 (21 postnatal: 10 intervention, 11 control); follow-up complete for all participants	Double-blind RCT; overall high risk of bias (other bias); small number of postnatal women (42%) (n<30): unable to obtain data for postnatal subgroup

SAYIN 200534

Population: Postnatal women with pre-eclampsia, severe pre-eclampsia, superimposed pre-eclampsia or eclampsia
Setting: Tertiary referral centres (Turkey)
Intervention: Nifedipine 10mg PO 6-hourly until BP <150/100mmHg for 48 hours
Comparison: Methyldopa 250mg PO 8-hourly

Primary outcome	Treatment effect	Number of participants	Quality of the evidence
SBP + DBP (time to target <150/100mmHg)	Nifedipine group 6.7±2.5 days; methyldopa group 8.6±5.5 days. Difference -1.9 days (NS).	83 (42 intervention, 41 control); follow-up complete for all participants	Open-label RCT; overall high risk of bias (multiple domains)

Vasodilators

PALOT 1979[36]

Population: Postnatal women with 'arterial hypertensions of labour and the postpartum period'
Setting: Not specified (France)
Intervention: Hydralazine 5mg IV stat then 1% IV infusion, furosemide 20mg IV stat and 30% hypertonic glucose
Comparison: Clonidine IV and furosemide 20mg IV stat

Primary outcome	Treatment effect	Number of participants	Quality of the evidence
Maternal morbidity (development of pre-eclampsia with severe features)	Hydralazine group: no women developed eclampsia, clonidine group: 2 women developed eclampsia. No statistical analysis.	54 (11 intervention, 24 control, 19 non-systematic treatment); completeness of follow-up not specified	Retrospective cohort study; overall high risk of bias (comparability); no statistical analysis

GRIFFIS 1989[38 39]

Population: Postnatal women with pre-eclampsia
Setting: Tertiary referral centres (USA)
Intervention: Hydralazine 20mg IM 6-hourly for 24h
Comparison: Methyldopa 250mg IV 6-hourly for 24h

Primary outcome	Treatment effect	Number of participants	Quality of the evidence
MAP (mean at 6 and 12 hours)	6 hours: hydralazine group 104.5mmHg, methyldopa group 112mmHg. Difference -7.5mmHg (p=0.0057). 12 hours: hydralazine group 100mmHg, methyldopa group108mmHg. Difference -8mmHg (NS).	26 (12 intervention, 14 control); follow-up complete for all participants	Open-label RC; overall high risk of bias (multiple domains); small sample size (n<30)

WALSS RODRIGUEZ 1991[40]

Population: Postnatal women with severe pre-eclampsia
Setting: Not specified (Mexico)
Intervention: Hydralazine 40mg PO 6-hourly, duration not specified + if DBP >110mmHg PRN nifedipine 10mg sublingual every 30 minutes, to maximum of 3 doses (30mg)
Comparison: Nifedipine 10mg sublingual every 30 minutes if DBP ≥110mmHg

Primary outcome	Treatment effect	Number of participants	Quality of the evidence
SBP (mean)	Hydralazine group 143.6mmHg, nifedipine group138.0mmHg. Difference 5.6mmHg (NS).	38 (18 intervention, 20 control); completeness of follow-up not specified	Open-label RCT; overall high risk of bias (multiple domains)
**BEGUM 2002**[17]<br>**Population:** Antenatal and postnatal women with eclampsia<br>**Setting:** Tertiary referral centres (Bangladesh)<br>**Intervention:** Hydralazine 5mg then 2mg IV bolus every 15 minutes until DBP 90-95mmHg<br>**Comparison:** Hydralazine 20mg /200ml normal saline IV infusion; 10 drops per min, increased by 5 drops at 15 min intervals; until DBP 90-95mmHg			
**Primary outcome**	**Treatment effect**	**Number of participants**	**Quality of the evidence**
DBP (time to target 90-95mmHg)	Bolus hydralazine group 65.23±23.38 minutes, hydralazine infusion group 186.36±79.77 minutes. Difference -121.13 minutes (*p*<0.001).	77 (15 postnatal: 9 intervention, 6 control); completeness of follow-up not specified	Open-label RCT; overall high risk of bias (multiple domains); small number of postnatal women (19%) (n<30): unable to obtain data for postnatal subgroup
**VIGIL DE GRACIA 2007**[35]<br>**Population:** Postnatal women with severe gestational hypertension, severe pre-eclampsia or super-imposed pre-eclampsia<br>**Setting:** Tertiary referral centres (Panama)<br>**Intervention:** Hydralazine 5mg IV every 20 minutes until BP <160/110mmHg or maximum 5 doses<br>**Comparison:** Labetalol 20mg, then 40mg, then 80mg IV every 20 minutes until BP <160/110mmHg or maximum 5 doses (300mg)			
**Primary outcome**	**Treatment effect**	**Number of participants**	**Quality of the evidence**
SBP + DBP (persistent hypertension >=160/110mmHg after 5 doses of medication)	Hydralazine group 0/42, labetalol group 1/40 (NS).	82 (42 intervention, 40 control); follow-up complete for all participants	Open-label RCT; overall high risk of bias (multiple domains)
**HENNESSY 2007**[23]<br>**Population:** Antenatal and postnatal women with pre-eclampsia, superimposed pre-eclampsia or essential hypertension<br>**Setting:** Tertiary referral (Australia)<br>**Intervention:** Diazoxide 15mg IV every 3 minutes, until target BP (140/90mmHg) reached or maximum cumulative dose 300mg<br>**Comparison:** Hydralazine 5mg IV every 20 minutes, until target BP (140/90mmHg) reached or maximum cumulative dose 15mg			
**Primary outcome**	**Treatment effect**	**Number of participants**	**Quality of the evidence**
SBP + DBP (proportion achieving target BP <=140/90mmHg)	Diazoxide group 67%, hydralazine group 43% (*p*<0.01).<br>RR 0.637 (95% CI 0.46 to 0.89) for not reaching target BP with intervention.	124 total (37 postnatal: 11 intervention, 16 control); follow-up complete for all participants	Open-label RCT; overall high risk of bias (multiple domains); small proportion of postnatal women (30%): unable to obtain data for postnatal subgroup
**Beta-blockers**			
**GARDEN 1982**[24]<br>**Population:** Antenatal and postnatal women with severe pre-eclampsia or eclampsia<br>**Setting:** Tertiary referral (South Africa)<br>**Intervention:** Labetalol 200mg/200ml 5% dextrose, 20mg/h IV infusion, doubled every 30 minutes until DBP <100mmHg or maximum dose 160mg/hour<br>**Comparison:** Dihydralazine 100mg/200ml 5% dextrose, 10mg/h IV infusion, doubled every 30 minutes until DBP <100mmHg or maximum dose 80mg/hour			
**Primary outcome**	**Treatment effect**	**Number of participants**	**Quality of the evidence**
DBP (proportion achieving target DBP 90-100mHg within 2 hours)	Labetalol group 5/6, dihydralazine group 2/6. No statistical analysis.	12 total (6 postnatal: 3 intervention, 3 control); follow-up complete for all participants	RCT (blinding not specified); overall high risk of bias (other bias); very small sample size (n<15): unable to obtain data for postnatal subgroup
**FIDLER 1982**[42]<br>**Population:** Postnatal women with gestational hypertension<br>**Setting:** Tertiary referral (UK)<br>**Intervention:** Timolol 5mg PO 8-hourly for 9 days<br>**Comparison:** Methyldopa 250mg PO 8-hourly for 9 days			
**Primary outcome**	**Treatment effect**	**Number of participants**	**Quality of the evidence**
DBP (day 1)	Timolol group 88.7mmHg, methyldopa group 93.8mmHg. Difference -5.1mmHg (*p*<0.05).	80 (40 intervention, 40 control); follow-up complete in 79/80 (99%)	RCT (blinding not specified); overall high risk of bias (multiple domains)
**MABIE 1987**[22]<br>**Population:** Antenatal and postnatal women with pre-eclampsia, superimposed pre-eclampsia, eclampsia or essential hypertension<br>**Setting:** Tertiary referral (USA)<br>**Intervention:** Labetalol 20mg IV every 10 minutes then escalating until DBP <100mmHg or maximum cumulative dose reached (300mg)<br>**Comparison:** Hydralazine 5mg IV every 10 minutes until DBP <100mmHg			

**Primary outcome**	**Treatment effect**	**Number of participants**	**Quality of the evidence**
MAP (mean maximal decrease)	Labetalol group 25.5±11.2mmHg, hydralazine group 33.3±13.2mmHg. Difference -7.8mmHg (*p*=0.02).	60 (41 postnatal: 27 intervention, 14 control); follow-up complete for all participants	Open-label RCT; overall high risk of bias (multiple domains)
**SHUMARD 2016**[41]<br>**Population:** Postnatal women with gestational hypertension or pre-eclampsia<br>**Setting:** Not specified (USA)<br>**Intervention:** Labetalol PO (variable dose and frequency)<br>**Comparison:** Nifedipine PO (variable dose and frequency)			
**Primary outcome**	**Treatment effect**	**Number of participants**	**Quality of the evidence**
Length of hospital stay after delivery	Labetalol group 3.5 days, nifedipine group 3.6 days. Difference -0.1 days (NS).	128 (42 intervention, 86 control); follow-up complete for all participants	Retrospective cohort study; overall high risk of bias (comparability); conference abstract only, authors did not provide further data
**SHARMA 2017**[27,28]<br>**Population:** Postnatal women with gestational hypertension or pre-eclampsia<br>**Setting:** Tertiary referral (USA)<br>**Intervention:** Labetalol 200mg PO 12-hourly<br>**Comparison:** Nifedipine XL 30mg PO once daily			
**Primary outcome**	**Treatment effect**	**Number of participants**	**Quality of the evidence**
SBP + DBP (time to sustained BP control: absence of severe hypertension for >=12 hours)	Labetalol group 37.6 hours, nifedipine group 38.2 hours. Difference -0.6 hours (NS).	50 (25 intervention, 25 control); follow-up complete for all participants	Open-label RCT; overall high risk of bias (multiple domains)
**Other antihypertensive medications**			
**GAISIN 2013**[25]<br>**Population:** Postnatal women with pre-eclampsia, super-imposed pre-eclampsia or essential hypertension<br>**Setting:** Not specified (Russia)<br>**Intervention:** Indapamide 1.5mg PO OD, duration unclear<br>**Comparison:** Adjusted dose methyldopa			
**Primary outcome**	**Treatment effect**	**Number of participants**	**Quality of the evidence**
SBP + DBP	Indapamide group 113±6/74±4mmHg, methyldopa group 116±5/75±4mmHg. Difference -3/+1mmHg (NS).	30 (15 intervention, 15 control); completeness of follow-up not specified	Open-label RCT; overall high risk of bias (multiple domains); conference abstract only, authors did not provide further data
**GAISIN 2014**[37]<br>**Population:** Postnatal women with pre-eclampsia<br>**Setting:** Not specified (Russia)<br>**Intervention:** Indapamide 1.5mg PO once daily + ursodeoxycholic acid 250mg PO three times daily, duration unclear<br>**Comparison:** Adjusted dose methyldopa			
**Primary outcome**	**Treatment effect**	**Number of participants**	**Quality of the evidence**
SBP + DBP	Indapamide group 122±6/75±4 mmHg, methyldopa group 126±6/78±5mmHg. Difference -4/-3mmHg (NS).	30 (allocation not described); completeness of follow-up not specified	Open-label RCT; overall high risk of bias (multiple domains); conference abstract only, authors did not provide further data; number of participants in each group not stated
**KREBS 1956**[43,44]<br>**Population:** Postnatal women with gestational hypertension, pre-eclampsia, severe pre-eclampsia or eclampsia<br>**Setting:** Not specified (Germany)<br>**Intervention:** Reserpine 0.25mg PO or intramuscular 6-8 hourly for 7 days<br>**Comparison:** Phenobarbital			
**Primary outcome**	**Treatment effect**	**Number of participants**	**Quality of the evidence**
SBP + DBP (maximal reduction)	Reserpine halved time to maximal BP reduction (no further details reported). No statistical analysis.	140 (70 intervention, 70 control); completeness of follow-up not specified	Retrospective cohort study; overall high risk of bias (selection and outcome assessment); no statistical analysis
**NORONHA NETO 2016**[29-31]<br>**Population:** Postnatal women with severe HDP<br>**Setting:** Tertiary referral (Brazil)<br>**Intervention:** Clonidine 0.1mg PO repeated every 20 minutes to max 6 doses<br>**Comparison:** Captopril 25mg PO repeated every 20 minutes to max 6 doses			

Primary outcome	Treatment effect	Number of participants	Quality of the evidence
SBP + DBP (episodes SBP ≥180mmHg and/or DBP ≥110mmHg)	Clonidine group 2.1±2.1 episodes, captopril group 3.5±4.7 episodes. Difference -1.4 episodes (NS).	90 (45 intervention, 45 control); completeness of follow-up not specified	Double-blind RCT; overall low risk of bias

DIURETICS (4 studies)

MATTHEWS 1997[46]

Population: Postnatal women with severe pre-eclampsia or eclampsia
Setting: Tertiary referral centres (UK)
Intervention: Furosemide 40mg PO once daily for 7 days
Comparison: Placebo

Primary outcome	Treatment effect	Number of participants	Quality of the evidence
MAP (decrease)	Intervention group -10.6mmHg, control group -9.75mmHg. Difference -0.85mmHg (NS).	19 (10 intervention, 9 control); follow-up complete in 18/19 (95%)	Double-blind RCT; overall high risk of bias (other bias); small sample size (n<30)

ASCARELLI 2005[16]

Population: Postnatal women with pre-eclampsia, severe pre-eclampsia or superimposed pre-eclampsia
Setting: Tertiary referral centres (USA)
Intervention: Furosemide 20mg PO once daily + potassium 20mEq PO once daily for 5 days
Comparison: No intervention

Primary outcome	Treatment effect	Number of participants	Quality of the evidence
SBP	No significant difference between groups (details not reported). Severe pre-eclampsia (n=70) day 2 SBP furosemide group 142±13mmHg, usual care group 153±19mmHg. Difference -11mmHg (*p*<0.004).	264 (132 intervention, 132 control); completeness of follow-up not specified.	Open-label RCT; overall high risk of bias (multiple domains)

AMORIM 2015[45]

Population: Postnatal women with severe pre-eclampsia
Setting: Tertiary referral (Brazil)
Intervention: Furosemide 40mg PO once daily for maximum 5 days
Comparison: Placebo

Primary outcome	Treatment effect	Number of participants	Quality of the evidence
SBP + DBP	Furosemide group had significantly improved SBP + DBP. Magnitude of difference not reported (*p*<0.001).	120 (allocation not described); follow-up complete in 118/120 (98%).	Double-blind RCT; overall high risk of bias (reporting bias); conference abstract only, authors did not provide further data; number of participants in each group not stated

VEENA 2017[19]

Population: Postnatal women with severe pre-eclampsia
Setting: Tertiary referral centre (India)
Intervention: Furosemide 10mg PO once daily plus nifedipine 10mg PO three times daily for 3 days
Comparison: Nifedipine 10mg PO three times daily for 3 days

Primary outcome	Treatment effect	Number of participants	Quality of the evidence
SBP + DBP	No significant difference between groups (absolute values and differences not reported, *p*=0.457 for SBP and *p*=0.642 for DBP).	100 (50 intervention, 50 control); follow-up complete in 98/100 (98%)	Open-label RCT; overall high risk of bias (multiple domains)

OTHER DRUGS (7 studies)

Selective 5-HT antagonists

WEINER 1982[48]

Population: Postnatal women with severe pre-eclampsia
Setting: Tertiary referral (USA)
Intervention: R41468 IV (dose not specified) bolus then infusion for 90 minutes
Comparison: Placebo

Primary outcome	Treatment effect	Number of participants	Quality of the evidence
MAP (mean maximal decline)	SSRI group -31.6mmHg, placebo group -6.0mmHg. Difference -25.6mmHg (p<0.001).	5 (crossover); follow-up complete in all participants	Double blind RCT (crossover); overall high risk of bias (other bias); conference abstract only, authors did not provide further data; very small sample size (n<15)

WEINER 1984[49]

Population: Postnatal women with pre-eclampsia and super-imposed pre-eclampsia
Setting: Tertiary referral (USA)
Intervention: Ketanserin 10mg IV bolus then 4mg/hr IV infusion. Repeat bolus after 5 minutes if no response.
Comparison: Placebo

Primary outcome	Treatment effect	Number of participants	Quality of the evidence
SBP + DBP (mean maximal decline)	SSRI group -41/-34mmHg, placebo group -7/-7mmHg. Difference -34/-27mmHg (p<0.001).	20 (crossover); follow-up complete in all participants	Double blind RCT (crossover); overall high risk of bias (other bias); small sample size (n<30)

MONTENEGRO 1985[50]

Population: Postnatal women with pre-eclampsia
Setting: Tertiary referral (USA)
Intervention: Ketanserin 10mg IV bolus, repeated if no response. If no response to second bolus IV infusion 4mg/hr (increments of 2mg/hr every 10 minutes to max 12mg/hr).
Comparison: Placebo

Primary outcome	Treatment effect	Number of participants	Quality of the evidence
MAP	SSRI group had significantly improved MAP, over 30 minutes after drug administered. F = 9.66 (*p*<0.01)	30 (crossover); follow-up complete in 23/30 (77%)	Double blind RCT (crossover); overall high risk of bias (multiple domains)

Alternative therapies

HLADUNEWICH 2006[51]

Population: Postnatal women with pre-eclampsia
Setting: Tertiary referral (USA)
Intervention: L-arginine 3.5g PO four times daily OR L-arginine 10g IV three times daily (if unable to take PO) for 3-9 days postpartum
Comparison: Placebo

Primary outcome	Treatment effect	Number of participants	Quality of the evidence
MAP	Day 3: L-arginine group 102±12mmHg, placebo group 103±12mmHg. Difference -1mmHg (NS). Day 10: L-arginine group 98±14mmHg, placebo group 96±1mmHg. Difference 2mmHg (NS).	45 (22 intervention, 23 control); follow-up complete in 39/45 (87%)	Double blind RCT; overall high risk of bias (multiple domains)

LIU 2009[52]

Population: Postnatal women with severe pre-eclampsia
Setting: District general (China)
Intervention: Shengkangbao 10g PO or IV twice daily for 3 weeks
Comparison: No intervention

Primary outcome	Treatment effect	Number of participants	Quality of the evidence
Percentage of cases with positive albuminuria	3 weeks: shengkangbao group 0.7+/-0.8% positive albuminuria, usual care group 1.5+/-0.9%. Difference -0.8% (*p*<0.01).	77 (allocation not described); follow-up complete in 72/77 (94%)	Open-label quasi-randomised study; overall high risk of bias (multiple domains)

Steroids

BARRILLEAUX 2005[53 54]

Population: Postnatal women with severe pre-eclampsia and eclampsia
Setting: Tertiary referral (USA)
Intervention: Dexamethasone 10mg x2, then 5mg x 2 IV 12-hourly for 48 hours
Comparison: Placebo (IV saline)

Primary outcome	Treatment effect	Number of participants	Quality of the evidence
Antihypertensive medication requirement	Dexamethasone group 38/77 (49%), placebo group 31/80 (39%) required antihypertensive treatment in the first 48h PN. Difference 10% (NS).	157 (77 intervention, 80 control); follow-up complete in 155/157 (99%)	Double blind RCT; overall high risk of bias (reporting bias)

Atrial natriuretic peptide

SHIGEMITSU 2015[47]

Population: Postnatal women with severe pre-eclampsia, HELLP syndrome or placental abruption
Setting: Tertiary referral (Japan)
Intervention: Carperitide (no further details supplied)
Comparison: No intervention

Primary outcome	Treatment effect	Number of participants	Quality of the evidence
MAP	Carperitide group had significantly improved MAP at 48 hours. Magnitude of difference not reported, no *p* value presented.	16 (6 intervention, 10 control); follow-up complete for all participants	Retrospective cohort study; overall high risk of bias (comparability); conference abstract only, authors did not provide further data; small sample size (n<30)

UTERINE CURETTAGE (8 studies)			
**SALVATORE 1967[58]**<br>**Population:** Postnatal women with severe pre-eclampsia or eclampsia<br>**Setting:** Tertiary referral (Brazil)<br>**Intervention:** Uterine curettage<br>**Comparison:** No intervention			
**Primary outcome**	**Treatment effect**	**Number of participants**	**Quality of the evidence**
SBP + DBP (proportion achieving target <140/90mmHg)	24 hours: curettage group 45%, usual care group 11%. No statistical analysis. 48 hours: curettage group 70%, usual care group 29%. No statistical analysis.	48 (20 intervention, 28 control; follow-up complete for all participants	Prospective cohort study; overall high risk of bias (comparability); significant differences in study groups (9/20 intervention group eclamptic at enrolment, 28/28 control group)
**MAGANN 1993[59]**<br>**Population:** Postnatal women with severe pre-eclampsia<br>**Setting:** Tertiary referral (USA)<br>**Intervention:** Uterine curettage<br>**Comparison:** No intervention			
**Primary outcome**	**Treatment effect**	**Number of participants**	**Quality of the evidence**
MAP	Curettage group had significantly improved MAP to 24 hours after birth. Difference -6 to -10mmHg (16 hours *p*<0.0002).	32 (16 intervention, 16 control); completeness of follow-up not specified	Open-label RCT; overall high risk of bias (multiple domains)
**MAGANN 1994[60]**<br>**Population:** Postnatal women with severe pre-eclampsia<br>**Setting:** Tertiary referral (USA)<br>**Intervention:** Uterine curettage<br>**Comparison:** Oral nifedipine OR no intervention			
**Primary outcome**	**Treatment effect**	**Number of participants**	**Quality of the evidence**
MAP	Curettage group had significantly improved MAP 8-48 hours after birth. Difference -9 to -13mmHg (*p*=0.0017). No difference between curettage and nifedipine.	45 (15 intervention, 15 each control group); completeness of follow-up not specified	Open-label RCT; overall high risk of bias (multiple domains)
**GOCMEN 1996[57]**<br>**Population:** Postnatal women with pre-eclampsia<br>**Setting:** Tertiary referral (Turkey)<br>**Intervention:** Uterine curettage<br>**Comparison:** No intervention			
**Primary outcome**	**Treatment effect**	**Number of participants**	**Quality of the evidence**
MAP	Curettage group had significantly improved MAP to 24 hours after birth. Magnitude of difference not reported (*p*=0.01).	50 (30 intervention, 20 control); completeness of follow-up not specified	Prospective cohort study; overall high risk of bias (comparability and outcome assessment); conference abstract only, authors did not provide further data
**GOMEZ 2005[61]**<br>**Population:** Postnatal women with severe pre-eclampsia<br>**Setting:** Tertiary referral (Peru)<br>**Intervention:** Uterine curettage<br>**Comparison:** No intervention			
**Primary outcome**	**Treatment effect**	**Number of participants**	**Quality of the evidence**
MAP	Intervention group had significantly improved MAP. Time point not specified. Magnitude of difference not reported (*p*<0.001).	86 (27 intervention, 59 control); completeness of follow-up not specified	Open-label RCT; overall high risk of bias (multiple domains); conference abstract only, authors did not provide further data
**ALKAN 2006[62]**<br>**Population:** Postnatal women with severe pre-eclampsia<br>**Setting:** Tertiary referral (Turkey)<br>**Intervention:** Uterine curettage<br>**Comparison:** No intervention			
**Primary outcome**	**Treatment effect**	**Number of participants**	**Quality of the evidence**
MAP	24 hours: curettage group 103.4±7.8mmHg, usual care group 110.2±4.8. Difference -6.8mmHg (*p*<0.05).	56 (31 intervention, 25 control); follow-up complete for all participant	Open-label RCT; overall high risk of bias (multiple domains)

RAGAB 2013[15]

Population: Postnatal women with severe pre-eclampsia or eclampsia
Setting: Tertiary referral (Egypt)
Intervention: Uterine curettage
Comparison: No intervention

Primary outcome	Treatment effect	Number of participants	Quality of the evidence
MAP	6 hours: curettage group 140.1±6.12mmHg, usual care group 152.4±3.7mmHg. Difference -12.3mmHg (*p*=0.02). 24 hours: curettage group 101.4±7.14mmHg, usual care group 110.6±2.22mmHg. Difference -9.2mmHg (*p*=0.01).	420 (220 intervention, 200 control); follow-up complete for all participants	Open-label RCT; overall high risk of bias (multiple domains)

MALLAPUR 2015[18]

Population: Postnatal women with severe pre-eclampsia or eclampsia
Setting: Tertiary referral (India)
Intervention: Uterine curettage
Comparison: No intervention

Primary outcome	Treatment effect	Number of participants	Quality of the evidence
MAP	From 4 hours after birth: curettage group 116±4.4mmHg, usual care group 123.6±6.1mmHg. Difference -7.6mmHg (*p*<0.001).	100 (50 intervention, 50 control); completeness of follow-up not specified	Open-label RCT; overall high risk of bias (multiple domains); conference abstract only, authors did not provide further data

ORGANISATION OF CARE (2 studies)

YORK 1997[26]

Population: Postnatal women with pre-eclampsia or essential hypertension, or diabetes
Setting: Tertiary referral (USA)
Intervention: Nurse specialist follow-up
Comparison: No intervention

Primary outcome	Treatment effect	Number of participants	Quality of the evidence
Postnatal readmission to secondary care	No significant difference between groups.	96 (44 intervention, 52 control); completeness of follow-up not specified	Open-label RCT; overall high risk of bias (multiple domains); population mixed diabetes and/or hypertension – unable to separate

BIBBO 2014[33]

Population: Postnatal women with pre-eclampsia
Setting: Tertiary referral (USA)
Intervention: Specialised postpartum clinic
Comparison: No intervention

Primary outcome	Treatment effect	Number of participants	Quality of the evidence
Postnatal readmission to secondary care and triage visits	Clinic group 21.7%, usual care group 8.7%. Difference 13% (*p*<0.039).	138 (69 intervention, 69 control); completeness of follow-up not specified.	Retrospective cohort study; overall high risk of bias (comparability); conference abstract only, authors did not provide further data
