# Supplementary material for: Postpartum management of hypertensive disorders of pregnancy: a systematic review
Source: BMJ Open. 2017 Nov 28;7(11):e018696. doi: 10.1136/bmjopen-2017-018696 (PMC5719299; doi:10.1136/bmjopen-2017-018696)
Supplement: Supplementary file 6 [file bmjopen-2017-018696supp006.pdf]

Appendix S6: Risk of bias in included studies (n=38)

Appendix S6a: Risk of bias in included RCTs and quasi-randomised studies (n=31)

Study ID	Random sequence generation	Allocation concealment	Blinding of participants and personnel	Blinding of outcome assessment	Incomplete outcome data	Selective reporting	Other bias
**ANTIHYPERTENSIVE MEDICATIONS**							
Fidler 1982[42]	Unclear	Unclear	Unclear	Unclear	High	High	Low
Garden 1982[24]	Unclear	Unclear	Unclear	Unclear	Low	Unclear	High
Mabie 1987[22]	Unclear	Unclear	High	High	Low	Low	High
Griffis 1989[38 39]	Unclear	Low	High	High	High	High	High
Barton 1990[32]	Low	Low	Low	Low	Low	Low	Low
Walss Rodriguez 1991[40]	Low	Low	High	High	Unclear	Unclear	Low
Vermillion 1999[21]	Low	Low	Low	Low	Low	Low	High
Begum 2002[17]	High	High	High	High	Unclear	Unclear	High
Sayin 2005[34]	Unclear	Unclear	High	High	Low	Unclear	High
Hennessy 2007[23]	Unclear	Low	High	High	Low	Low	High
Vigil-de-Gracia 2007[35]	Low	Low	High	High	Low	Low	Low
Gaisin 2013[25]	Unclear	Unclear	High	High	Unclear	High	High
Gaisin 2014[37]	Unclear	Unclear	High	High	Unclear	Unclear	High
Noronha Neto 2016[29-31]	Low	Low	Low	Unclear	Low	Low	Low
Sharma 2017[27 28]	Low	Low	High	High	Unclear	Low	Low
**DIURETICS**							
Matthews 1997[46]	Unclear	Low	Low	Low	Low	Unclear	High
Ascarelli 2005[16]	Unclear	Low	High	High	Unclear	High	Low
Amorim 2015[45]	Low	Low	Low	Low	Low	High	Low
Veena 2017[19]	Low	Low	High	High	Unclear	Unclear	Unclear
**OTHER DRUGS**							
Weiner 1982[48]	Unclear	Unclear	Low	Low	Unclear	Unclear	High
Weiner 1984[49]	Low	Unclear	Low	Low	Low	Unclear	High
Montenegro 1985[50]	Unclear	Unclear	Low	Low	High	High	High
Barrilleaux 2005[53 54]	Low	Low	Low	Low	Low	High	High
Hladunewich 2006[51]	Low	Low	Low	Low	High	High	High
Liu 2009[52]	High	High	High	High	High	Unclear	High
**UTERINE CURETTAGE**							
Magann 1993[59]	Low	Low	High	High	Unclear	Unclear	Low
Magann 1994[60]	Low	Unclear	High	High	Unclear	Unclear	Low
Gomez 2005[61]	Unclear	Unclear	High	High	Unclear	High	Low
Alkan 2006[62]	Unclear	Unclear	High	High	Low	High	High
Ragab 2013[15]	Low	Low	High	High	Low	Low	Low
Mallapur 2015[18]	Low	Unclear	High	High	Unclear	Unclear	High
**ORGANISATION OF CARE**							
York 1997[26]	Unclear	Low	High	High	Unclear	Unclear	High

Appendix S6b: Risk of bias in included cohort studies (n=7)

Study ID	Selection				Comparability[1]	Outcome		
	Representative-ness[2]	Selection of non-exposed[3]	Ascertainment of exposure[4]	Outcome of interest not present at start		Assessment[5]	F/U long enough	Adequacy of F/U[6]
**ANTIHYPERTENSIVE MEDICATIONS**								
Krebs 1956[43 44]	Low (a)	Low (a)	Unclear (d)	Low (Yes)	Low (a)	High (b)	Low (Yes)	Unclear (d)
Palot 1979[36]	Unclear (d)	Low (a)	Unclear (d)	Low (Yes)	High (Neither)	Unclear (d)	Low (Yes)	Unclear (d)
Shumard 2016[41]	Low (a)	Low (a)	Low (a)	Low (Yes)	High (Neither)	Low (a)	Low (Yes)	Low (a)
**OTHER DRUGS**								
Shigemitsu 2015[47]	Unclear (d)	Unclear (c)	Low (a)	Low (Yes)	High (Neither)	Low (a)	Low (Yes)	Unclear (d)
**UTERINE CURETTAGE**								
Salvatore 1967[58]	High (b)	High (b)	Low (a)	Low (Yes)	High (Neither)	Low (a)	Low (Yes)	Low (a)
Gocmen 1996[57]	Unclear (d)	Unclear (c)	Unclear (d)	Low (Yes)	High (Neither)	Unclear (d)	High (No)	Unclear (d)
**ORGANISATION OF CARE**								
Bibbo 2014[33]	Unclear (d)	Unclear (c)	Unclear (d)	Low (Yes)	High (Neither)	Unclear (d)	Low (Yes)	Unclear (d)

[1] (a) study controls for most important factor; (b) study controls for any additional factor

[2] (a) truly representative of the average in the community; (b) somewhat representative of the average in the community; (c) selected group of users e.g. nurses, volunteers; (d) no description of the derivation of the cohort

[3] (a) drawn from the same community as the exposed cohort; (b) drawn from a different source; (c) no description of the derivation of the non-exposed cohort

[4] (a) secure record (e.g. surgical record); (b) structured interview; (c) written self-report; (d) no description

[5] (a) independent blind assessment; (b) record linkage; (c) self-report; (d) no description

[6] (a) complete follow-up; (b) subjects lost to follow-up unlikely to introduce bias (>90% follow-up rate); (c) follow up rate <90% and no description of those lost; (d) no statement
