# Supplementary material for: Postpartum management of hypertensive disorders of pregnancy: a systematic review
Source: BMJ Open. 2017 Nov 28;7(11):e018696. doi: 10.1136/bmjopen-2017-018696 (PMC5719299; doi:10.1136/bmjopen-2017-018696)
Supplement: Author's manuscript [file bmjopen-2017-018696.draft_revisions.pdf]

BMJ Open is committed to open peer review. As part of this commitment we make the peer review history of every article we publish publicly available.

When an article is published we post the peer reviewers' comments and the authors' responses online. We also post the versions of the paper that were used during peer review. These are the versions that the peer review comments apply to.

The versions of the paper that follow are the versions that were submitted during the peer review process. They are not the versions of record or the final published versions. They should not be cited or distributed as the published version of this manuscript.

BMJ Open is an open access journal and the full, final, typeset and author-corrected version of record of the manuscript is available on our site with no access controls, subscription charges or pay-per-view fees (http://bmjopen.bmj.com).

If you have any questions on BMJ Open's open peer review process please email editorial.bmjopen@bmj.com

BMJ Open

Postpartum management of hypertensive disorders of pregnancy: a systematic review

Journal:	*BMJ Open*
Manuscript ID	bmjopen-2017-018696
Article Type:	Research
Date Submitted by the Author:	14-Jul-2017
Complete List of Authors:	Cairns, Alexandra; University of Oxford Medical Sciences Division, Nuffield Department of Primary Care Health Sciences<br>Pealing, Louise; University of Oxford Medical Sciences Division, Nuffield Department of Primary Care Health Sciences<br>Duffy, James; University of Oxford Medical Sciences Division, Nuffield Department of Primary Care Health Sciences<br>Roberts, Nia; University of Oxford, UK, Bodleian Health Care Libraries,<br>Tucker, Katherine; University of Oxford, Nuffield Department of Primary Care<br>Leeson, Paul; University of Oxford, Division of Cardiovascular Medicine<br>MacKillop, Lucy; Oxford University Hospitals NHS Trust, Nuffield Department of Obstetrics & Gynaecology<br>McManus, Richard; University of Oxford, Dept of Primary Care Health Sciences
**Primary Subject Heading**:	Obstetrics and gynaecology
Secondary Subject Heading:	Evidence based practice
Keywords:	Preeclampsia, Gestational hypertension, Postpartum, Hypertensive disorders of pregnancy, Antihypertensive medication, Systematic review

SCHOLARONE™
Manuscripts

Postpartum management of hypertensive disorders of pregnancy: a systematic review

Dr Alexandra E. Cairns MA Hons (Oxon) BMBCh MRCOG[1], Dr Louise Pealing MA Hons (Cantab) MSc MBBS MRCP MRCGP[1], Dr James M.N. Duffy MBChB MRes BSc (Hons) PG HCL[1], Nia Roberts MSc Econ[2], Dr Katherine L. Tucker PhD[1], Professor Paul Leeson PhD MBBChir BSc (Hons) FRCP[3], Dr Lucy H. Mackillop MA (Oxon) BMBCh FRCP[4], Professor Richard J. McManus PhD MA MBBS FRCGP FRCP[1]

[1] Nuffield Department of Primary Care Health Sciences, University of Oxford, Oxford, United Kingdom.
[2] Knowledge Centre, Bodleian Libraries, University of Oxford, Oxford, United Kingdom.
[3] Cardiovascular Clinical Research Facility, Division of Cardiovascular Medicine, Radcliffe Department of Medicine, University of Oxford, Oxford, United Kingdom.
[4] Nuffield Department of Obstetrics and Gynaecology, University of Oxford, Oxford, United Kingdom.

Corresponding author: Dr Alexandra E. Cairns BMBCh MA Hons (Oxon) MRCOG

alexandra.cairns@phc.ox.ac.uk

+44 (0) 1865 617960

Nuffield Department of Primary Care Health Sciences, University of Oxford, Radcliffe Primary Care, Radcliffe Observatory Quarter, Woodstock Road, Oxford, OX2 6GG, United Kingdom.

Short title: Postnatal Hypertension Management

Competing interests statement: None of the authors has any conflicts of interest to declare.

For peer review only

For peer review only - http://bmjopen.bmj.com/site/about/guidelines.xhtml

Abstract

Objectives

Hypertensive disorders of pregnancy (HDP) affect one in ten pregnancies and often persist postpartum when complications can occur. We aimed to determine the effectiveness and safety of pharmacologic interventions, other interventions, and different care models for postpartum hypertension management.

Design

A systematic review was undertaken. Nine electronic databases, including Medline, were searched from inception to 16/03/2017. After duplicate removal, 4,561 records were screened. Two authors independently selected studies, extracted study characteristics and data, and assessed methodological quality.

Setting

Randomised controlled trials, case-control studies, and cohort studies from any country and healthcare setting.

Participants

Postnatal women with HDP.

Interventions

Therapeutic intervention for management of hypertension, compared with another intervention, placebo, or no intervention.

Primary and secondary outcome measures

Outcome data were collected for maternal mortality and severe morbidity; systolic, diastolic and mean arterial blood pressure (BP) control; and safety data. Secondary outcome data collected included the length of postnatal hospital stay and laboratory values.

Results

39 studies were included (n=2,901). Results were heterogeneous in terms of intervention, comparison and outcome requiring a narrative approach. There were insufficient data to recommend any single pharmacologic intervention. 18 studies reported calcium-channel blockers, vasodilators and beta-blockers lowered BP postpartum. 12 of these reported safety data. Limited data existed regarding management in the weeks following hospital discharge. Neither loop diuretics (three studies) nor corticosteroids (one study) produced clinical benefit.

For peer review only - http://bmjopen.bmj.com/site/about/guidelines.xhtml

Uterine curettage significantly reduced BP over the first 48 hours postpartum (range 6-13mmHg) compared to standard care (eight studies), with safety data only reported by 4/8 studies.

Conclusion

There was insufficient evidence to recommend a particular BP threshold, agent, or model of care but three classes of antihypertensive appeared variably effective. Further comparative research, including robust safety data, is required. Curettage reduced BP, but without adequate reporting of harms, so cannot currently be recommended.

Strengths and limitations of this study

- All types of intervention for the management of postpartum hypertension – medical, surgical and organisation of care – were eligible for inclusion in this review.
- Randomised controlled studies plus other experimental study designs (cohort studies, case-control studies and quasi-randomised studies) were included and no limitations were imposed in terms of language or publication date, resulting in a comprehensive review.
- This review highlights significant evidence gaps, demonstrating that further comparative research is required, particularly to clarify postpartum antihypertensive selection.
- Although 39 studies were included, the majority had a high risk of bias such that the evidence provided by this review is of low quality.
- The 39 studies reported a broad range of heterogeneous outcomes, limiting meaningful comparison.

Keywords

Preeclampsia, gestational hypertension, postpartum, hypertensive disorders of pregnancy, antihypertensive medication, systematic review

Abbreviations

BP Blood pressure

For peer review only - http://bmjopen.bmj.com/site/about/guidelines.xhtml

HDP Hypertensive disorders of pregnancy

MAP Mean arterial pressure

NICE National Institute of Health and Care Excellence

RCT Randomised controlled trial

SSRI Selective serotonin reuptake inhibitor

For peer review only

For peer review only - http://bmjopen.bmj.com/site/about/guidelines.xhtml

Introduction

Hypertensive disorders of pregnancy (HDP) often persist following delivery,[1] and occasionally arise de novo postpartum.[2] In both scenarios adverse events can occur during this period. Approximately one-third of eclampsia occurs postpartum, nearly half beyond 48 hours after childbirth.[3-5] Half of the women who sustain an intracerebral haemorrhage in association with preeclampsia do so following birth.[6] Women may enter the postnatal period requiring large doses of antihypertensive medication, but the majority will be treatment-free by three to six months.[1 7] This rapidly changing blood pressure (BP) poses a challenge in terms of appropriate antihypertensive selection and dose adjustment.

The National Institute for Health and Care Excellence (NICE) recommends frequent postnatal BP monitoring for women with both preeclampsia (every one to two days for two weeks) and gestational hypertension (at least once between day three and five).[8] The guideline stipulates thresholds for the increase or commencement (≥150/100mmHg) and the reduction or cessation (consider <140/90mmHg and reduce <130/80mmHg) of antihypertensive medication after birth. However, little detail is provided about frequency or proportion of dose reduction or how to manage multiple medications.[8] The American College of Obstetricians and Gynecologists recommend that BP be monitored in hospital (or with an equivalent level of outpatient surveillance) for 72 hours after birth, and checked again seven to ten days postpartum (sooner if a woman is symptomatic).[9] In line with NICE, they propose treating BP when ≥150/100mmHg, but add this should be on two measures, four to six hours apart. They make no suggestion regarding BP thresholds for medication reduction, implying uncertainty about when to decrease or stop treatment.

A Cochrane review (search date January 2013) evaluated medical interventions for prevention and treatment of postnatal hypertension. This was limited to randomised controlled trials (RCTs) and included only nine studies.[10] Given the paucity of evidence available, we have undertaken an updated systematic review of the postpartum management of hypertension in women with HDP with a broader scope: including the full range of interventions studied, and incorporating cohort and case-control studies, alongside RCTs. Our specific questions were: [1] How should BP be monitored in women with HDP postpartum? [2] What BP thresholds should be used for antihypertensive treatment initiation, adjustment and cessation postpartum? [3] Which antihypertensive medication(s) should be used in

For peer review only - http://bmjopen.bmj.com/site/about/guidelines.xhtml

postpartum in women with HDP? [4] What are the benefits and harms of other therapeutic interventions for women with HDP postpartum?

Material and methods

A protocol, with explicitly defined objectives, study selection criteria, and approaches to assessing study quality, outcomes and statistical methods, was developed (Appendix S1). This was registered with PROSPERO: International Prospective Register of Systematic Reviews (CRD42015015527) and is available online (http://www.crd.york.ac.uk/PROSPERO/display_record.asp?ID=CRD42015015527). We followed the guidelines for meta-analyses and systematic reviews outlined by the Preferred Reporting Items for Systematic Reviews and Meta-Analyses (PRISMA) statement (Appendix S2).[11]

A systematic literature review was undertaken to capture evidence from human studies regarding postpartum hypertension management in women with HDP, without restriction by language or publication date (Appendix S1). We searched the following databases, from inception to 16/03/2017: Cochrane Database of Systematic Reviews (CDSR), Database of Abstracts of Reviews of Effects (DARE) and Cochrane Central Register of Controlled Trials (CENTRAL), Cumulative Index to Nursing and Allied Health Literature (CINAHL), Embase, Medline, PsycINFO, Science Citation Index, Science (Web of Science Core Collection), Social Science Citation Index & Conference Proceedings Citation Index. We hand-searched reference lists and contacted relevant experts for potentially relevant studies, which might have been missed by electronic searches.[12]

We included RCTs, quasi-randomised studies, case-control studies, prospective and retrospective cohort studies, assessing interventions for hypertension management postpartum in women with HDP (gestational hypertension, pre-eclampsia, chronic hypertension and super-imposed pre-eclampsia). Consistent with guidance from Cochrane, conference abstracts were included.[5]

Two reviewers (AC/LP) independently screened the titles and abstracts, and then critically reviewed the full text of selected studies to assess eligibility. Discrepancies were resolved by discussion before independent extraction of relevant data by the two reviewers. For trials with multiple intervention arms, we extracted data from eligible comparison arms. Data were

For peer review only - http://bmjopen.bmj.com/site/about/guidelines.xhtml

extracted for the primary and secondary outcomes outlined in Table 1. Due to the heterogeneous nature of these studies, a narrative synthesis was undertaken.

Two reviewers (AC/LP) independently assessed each trial's methodological quality using the Cochrane Collaboration's tool for assessing the risk of bias in RCTs,[13] and the Newcastle-Ottawa scale for case-control and cohort studies.[14] A global assessment of bias across trials was made.

Results

Our searches yielded 7,105 records and after excluding duplicates, 4,561 titles and abstracts were screened (Figure 1). 80 full-text articles were assessed: 35 articles were excluded (Appendix S3). 45 articles, representing 39 studies (32 randomised trials, two prospective cohort studies, and five retrospective cohort studies) reporting data from 2,901 postnatal participants met our inclusion criteria (Appendix S4). 9/39 (23%) were published only as conference abstracts. No further details were made available following author contact.

A range of interventions was assessed including antihypertensive medications (18 studies, n=982), loop diuretics (four studies, n=503), parenteral steroids (one study, n=157), other medications (six studies, n=188), uterine curettage (eight studies, n=837) and novel models of care (two studies, n=234). 9/39 (23%) included ≥100 participants, and only two studies included ≥200 participants.[15 16] Four were from lower-middle-income settings[15 17-19] (classified according to the United Nations[20]), and 13/39 (33%) studies had follow-up periods longer than seven days (Appendix S4). Only 5/39 (13%) and 7/39 (18%) studies, respectively, reported maternal mortality or major maternal morbidity, and whilst the majority of studies did report some measure of BP control, three did not (Tables 2a&b). 19/39 (49%) studies reported safety data (Tables 2a&b).

5/39 (13%) studies (all evaluating antihypertensive medications) involved mixed antenatal and postnatal populations[17 21-24]. Authors were contacted to request their dataset for the postnatal participants, but no data were made available. 6/39 (15%) studies included participants with chronic hypertension alongside women with de novo HDP (gestational hypertension or pre-eclampsia).[22 23 25-31] 12/39 (31%) studies included women with eclampsia – in one all participants were eclamptic (Appendix S5).[17]

30/32 (94%) included RCTs were judged to be at high overall risk of bias, by both reviewers, according to the Cochrane tool, 23/32 (72%) for multiple domains. Only 2/32 (6%) were

For peer review only - http://bmjopen.bmj.com/site/about/guidelines.xhtml
For peer review only

thought to be clearly at low risk of bias.[29-32] All included cohort studies were deemed to have a high risk of bias in at least one domain of the Newcastle-Ottawa scale (Appendix S6).

How should blood pressure be monitored postpartum in women with hypertensive disorders of pregnancy?

No studies specifically addressed the frequency or method of postnatal BP monitoring. Two evaluated the impact of postpartum care organisation (n=234), using the postnatal readmission rate as their primary outcome (Appendix S4). Neither reported maternal mortality or morbidity, safety data nor any measure of BP control (Table 2b).[26 33]

One assessed introduction of a specialised postpartum clinic (no further details were given) and demonstrated an increased postnatal readmission and triage visit rate (22% intervention group, 9% control group: difference 13%, $p<0.04$) although 86% occurred before a participant was seen in the clinic.[33] The second study evaluated specialist nurse follow-up, including home visits and telephone contact, and reported no significant difference in the postnatal readmission rate compared to standard care.[26]

What blood pressure thresholds should be used for antihypertensive treatment initiation, adjustment and cessation postpartum?

No relevant studies identified.

Which antihypertensive medication(s) should be used postpartum in women with hypertensive disorders of pregnancy?

14 randomised trials (n=645), one quasi-randomised trial (n=15), and three retrospective cohort studies (n=322) evaluated antihypertensive medications (Appendix S4). Only three studies reported maternal mortality,[29-31 34 35] and three reported maternal morbidity: no differences between groups were reported (Table 2a).[29-31 35 36] 12 studies reported safety data, in comparisons between multiple classes of antihypertensive agents (Table 2a): no clear differences were established, although one study found a greater number of minor side effects reported with oral nifedipine than with oral labetalol.[27 28]

The vast majority of included studies evaluated either acute control of severe hypertension (7/18, 39%), or BP control in the few days after delivery, whilst women remained hospital inpatients (8/18, 44%). Only three studies, two published only as conference abstracts, evaluated BP control in the weeks and months following hospital discharge.[25 27 28 37]

For peer review only - http://bmjopen.bmj.com/site/about/guidelines.xhtml

Calcium-channel blockers

Three small studies examined oral nifedipine (n=135): nifedipine resulted in a greater decrease in MAP 18-24 hours after childbirth than placebo (intervention group 93.9±1.6mmHg, control group 100.2±2.6mmHg, difference 6.3mmHg, *p*<0.05), but not at other time points to 48 hours (one RCT, n=31).[32] Nifedipine controlled severe hypertension to <160/100mmHg more quickly than labetalol (intervention group 25.1±13.6 minutes, control group 43.6±25.4 minutes: difference 18.5 minutes, *p*=0.002; one RCT, n=21).[21] A single RCT (n=83), reported no significant difference in time taken to control BP to <150/100mmHg when comparing nifedipine with methyldopa.[34]

Vasodilators

Six studies looked at the use of vasodilators (n=252). All utilised hydralazine via a range of administration routes. Bolus intravenous hydralazine controlled severe hypertension more quickly than continuous infusion (intervention group 65.23±23.38 minutes, control group 186.36±79.77 minutes: difference -121.13 minutes, *p*<0.001); one quasi-randomised study, n=15 (postnatal)).[17] Intramuscular hydralazine produced a more significant improvement in MAP at six hours than intravenous methyldopa (intervention group 104.5mmHg, control group 112mmHg: difference -7.5mmHg *p*=0.0057) but not at other time points to 24 hours (one RCT, n=26).[38,39] There was no difference in BP control when comparing oral hydralazine with oral nifedipine (one RCT, n=38), or intravenous labetalol (one RCT, n=82).[35,40]

Bolus diazoxide was significantly more effective in achieving a target BP of ≤140/90mmHg than intravenous hydralazine (intervention group 67%, control group 43%: RR 0.64, 95% CI 0.46-0.89; one RCT, n=37 (postnatal)).[23] One retrospective cohort study did not present any statistical analysis.[36]

Beta-blockers

Five studies assessed the efficacy of beta-blockers (four RCTs and one retrospective cohort study, n=305). Two RCTs compared intravenous labetalol with intravenous hydralazine/dihydralazine: one involved only six postnatal women and presented no statistical analysis of the data.[24] The other found a significantly greater mean maximal decrease in MAP with intravenous labetalol (intervention group 25.5±11.2mmHg, control group 33.3±13.2mmHg: difference -7.8mmHg, *p*=0.02; one RCT, n=32 (postnatal)).[22] Results conflicted regarding whether oral labetalol was more or less effective than oral

For peer review only - http://bmjopen.bmj.com/site/about/guidelines.xhtml
For peer review only

nifedipine: a cohort study reported that labetalol controlled BP less rapidly than nifedipine (intervention group 2.7 days, control group 1.7 days: difference 1.0 days, p=0.0031; one retrospective cohort study, n=128).[41] However, this result was not replicated by an RCT, where the time to BP control was similar in the two groups (n=50).[27 28] Neither study demonstrated a difference in the postnatal length of stay (n=178). Timolol was effective in decreasing diastolic BP on day one postnatal when compared with methyldopa (intervention group 88.7mmHg, control group 93.8mmHg: difference -5.1mmHg; p<0.05; one RCT, n=80).[42]

Other antihypertensive medications

No statistically significant difference was found between oral clonidine and oral captopril in the incidence of episodes of severe hypertension postpartum (one RCT, n=90).[29-31] Two RCTs evaluating indapamide versus methyldopa found no difference in BP control over 6-12 months postpartum (n=60).[25 37] One retrospective cohort study (n=140) compared reserpine with phenobarbital: the results suggested that reserpine might achieve faster and greater BP reduction (data extracted from graphs; no statistical analysis). No adverse events were reported in the intervention group.[43 44]

What are the benefits and harms of other therapeutic interventions for women with hypertensive disorders of pregnancy postpartum?

Loop Diuretics

Four RCTs (n=503) examined loop diuretics versus placebo or usual care in postpartum hypertension management in women with HDP. None reported maternal mortality or safety data. Only two reported major maternal morbidity, neither demonstrating a difference between groups (Table 2b).[16 19]

One RCT (n=120) reported significant improvement in the primary outcome of mean systolic and diastolic BP with oral furosemide versus placebo (magnitude of difference or time points of measurements not stated, p<0.001).[45] This was not the case in the other placebo-controlled RCT, which found no significant difference (n=19).[46] Two further RCTs (n=364) found no significant difference in BP control with oral furosemide versus usual care.[16 19] In one of these, subgroup analysis of women with severe preeclampsia (n=70) found women who received oral furosemide had a significantly lower systolic BP day 2 postpartum (intervention group 142±13mmHg, control group 153±19mmHg: difference -11mmHg, p<0.004), but not at other time points.[16] In the other trial (n=100), furosemide reduced the need for additional

For peer review only - http://bmjopen.bmj.com/site/about/guidelines.xhtml

antihypertensive treatment during the three days of therapy (intervention group 8.0%, control group 26.0% difference 18%, p=0.017), but this difference did not persist to hospital discharge.[19]

Other drugs

Five RCTs, one quasi-randomised study and one retrospective cohort study investigated the utility of different drug classes in HDP postpartum (Appendix S5). Three studies reported safety data, but only one reported maternal mortality, demonstrating no difference between groups,[47] and none reported major maternal morbidity (Table 2b).

Three small, crossover RCTs examined the use of selective serotonin receptor inhibitors (SSRIs) compared with placebo (n=55). All studies showed a significant reduction in BP with SSRIs compared to placebo (range 25.6 – 34mmHg).[48-50] These data suggest efficacy for this drug class in hypertension management but do not provide any information regarding relative effectiveness compared to standard antihypertensive drugs. Only one study reported safety data: although no statistical analysis was performed, there were a number of side effects reported in the intervention group.[49]

Two studies evaluated alternative therapies (n=117): there was no difference in BP control with L-arginine supplementation compared with placebo (one RCT, n=45).[51] One reported accelerated recovery of albuminuria with the administration of shengkangbao (Chinese herbal medicine) versus placebo (one quasi-randomised study, n=72). However, the clinical relevance of this outcome is uncertain, there was no difference between groups in the secondary outcomes of systolic BP, diastolic BP or serum creatinine and no safety data were reported.[52]

A single RCT assessed corticosteroids in the management of severe preeclampsia postpartum (n=157).[53 54] No difference was demonstrated between groups in the primary outcome of antihypertensive medication requirement, or in the secondary outcomes of mean arterial pressure (MAP) or need for critical care admission, and no safety data were reported. There were small, statistically significant differences found in some laboratory values (platelet count, lactate dehydrogenase and aspartate transaminase). However, the authors acknowledged that the absolute differences were too small to be clinically relevant.[53]

A very small retrospective cohort study suggested an improvement in MAP with the addition of carperitide (atrial natriuretic peptide) to standard therapy (n=16), and no adverse effects

For peer review only - http://bmjopen.bmj.com/site/about/guidelines.xhtml

related to the intervention were reported.[47] However, the magnitude of the difference was not published, and the study was too small to draw any firm conclusions.

Uterine curettage

Six RCTs and two prospective cohort studies (n=837) have explored the role of uterine curettage in postpartum hypertension management. Uterine curettage is a similar process to that used in the surgical management of miscarriage: the lining of the uterus is scraped after completion of the third stage of labour in order to maximise placental tissue removal. This may be under direct vision following caesarean section, or via the transcervical route following vaginal birth. The latter approach may be ultrasound-guided and necessitates some form of anaesthesia. The theory underlying this intervention is that gestational hypertension and preeclampsia are placenta-mediated, and therefore ensuring complete evacuation of the uterus following childbirth may accelerate recovery.[55 56]

Seven studies explicitly stated they included both participants who delivered vaginally and those delivered by caesarean: four reported numbers undergoing vaginal delivery (n=248) and caesarean (n=321). One made no comment about the mode of birth.[57] Only one study reported maternal mortality: no difference between groups.[15] Two reported major maternal morbidity, but neither performed any statistical analysis (Table 2b). However, both studies did suggest a reduction in the absolute number of eclamptic seizures in the curettage group compared to no intervention.[15 58] In one, however, there was a relevant difference between the study groups: 28/28 (100%) in the control group were eclamptic at enrolment, compared to 9/20 (45%) in the intervention group.[58] Four studies reported safety data, with none reporting any complications related to the intervention (Table 2b).[59-62]

All eight studies compared curettage with standard care (i.e. no additional intervention), and all suggested that uterine curettage resulted in a significantly lower BP.[15 18 57-62] One of these had two control groups: standard care, and oral nifedipine; when compared to oral nifedipine, no difference was noted with curettage.[60]

Five studies reported the magnitude of the difference in MAP between curettage and standard care: range 6-13mmHg.[15 18 59 60 62] Only two of these reported BP data beyond 24 hours postpartum: one RCT reported a significantly lower MAP at 48 hours with curettage (intervention group 104mmHg, control group 113mmHg, difference 9mmHg, p=0.0017; n=45),[60] but the other RCT demonstrated no significant difference in MAP at 48 hours (n=420).[15]

For peer review only - http://bmjopen.bmj.com/site/about/guidelines.xhtml

One study demonstrated that a greater proportion of the intervention group attained the target BP of <140/90mmHg at 24 (intervention group 9/20 (45%), control group 3/28 (11%): difference 34%, no *p*-value quoted) and 48 hours postpartum (intervention group 14/20 (70%), control group 8/28 (29%): difference 41%, no *p*-value quoted).[58] Two studies did not present the size of the difference between groups.[57 61]

Discussion

This review found evidence demonstrating that calcium-channel blockers, vasodilators and beta-blockers lower BP postpartum, but no clear answer to which was most effective and should, therefore, be preferentially prescribed. All but two studies examined the acute control of severe hypertension or short term BP control whilst women remained in hospital postpartum,[25 37] and so provide little guidance about prescription in the weeks after discharge. Moreover these both examined thiazide diuretics, not recommended in the UK for use whilst breastfeeding.[8] Complete safety data were limited across trials, as were data regarding objective clinical outcomes and two further studies examined antihypertensive agents not recommended for use postpartum in the UK (methyldopa and reserpine).[63 64] One trial evaluated captopril at a much higher daily dose than the UK recommended daily starting dose.[64]

Uterine curettage is not currently recommended, due to safety concerns regarding additional anaesthetic and operative risks, and the availability of alternative treatments to lower BP, particularly in the context of vaginal birth.[65] However, the included studies consistently demonstrated that uterine curettage improved BP control versus standard care,[15 18 57-62] with one reporting an equivalent effect to oral nifedipine.[60] Amongst the limited safety data none reported an excess complication rate (infection or uterine damage) with curettage, but given the low incidence of operative complications, the total population (n=837) was likely insufficient to adequately address potential competing risks. Furthermore, these studies did not demonstrate any impact from curettage on maternal mortality or severe morbidity and concerns exist about some studies' methodology. The evidence reviewed is insufficient to recommend incorporation of this intervention into routine clinical practice.

Four trials evaluating loop diuretics failed to provide conclusive evidence of benefit. Three produced non-significant results in their main analysis,[16 19 46] and the single conference abstract which did suggest better BP control with oral furosemide, did not publish the magnitude of the difference, rendering it difficult to assess the clinical relevance.[45] In contrast

For peer review only - http://bmjopen.bmj.com/site/about/guidelines.xhtml

to the Cochrane review, we conclude that, at present, there is no evidence to support the routine use of diuretics postpartum.[10]

We found no adequate evidence to support alternative medications or a particular care model in the management of HDP postpartum. SSRIs substantially reduced BP versus placebo,[48-50] but no published data was identified comparing their efficacy with standard antihypertensive treatment, making it difficult to draw meaningful conclusions about their clinical application. Neither study evaluating postpartum care organisation reported maternal mortality or morbidity, or any measure of BP control, with both selecting postnatal readmissions as their primary outcome. An increased postnatal readmission rate, however, may not necessarily reflect harm: it might instead suggest that a particular model of care can better detect problems in the community and admit appropriately, ultimately resulting in a lower risk to patients.

In light of the heterogeneous nature of research in this field, when designing this review, we included all interventions targeting hypertension management, but not end-organ complications, including eclampsia. Therefore, trials evaluating magnesium sulphate were outside the scope of this review. We acknowledge the relevance of this therapy in women with severe pre-eclampsia, especially in the immediate postnatal period, and a Cochrane review suggests there is no uncertainty regarding the effectiveness of this therapy.[66]

A strength of this review is that cohort studies, case-control studies and quasi-randomised studies were eligible in addition to RCTs, and no language or date restrictions were imposed, resulting in a comprehensive review that provides evidence suggesting significant research gaps, consistent with the findings from the Cochrane review (2013).[10] The Cochrane review included only nine trials (author names in bold in Appendix S4). We believe our review adds to this, as an additional 30 studies are included (19 pre-dating the Cochrane search, and 11 subsequent to it), providing a current and complete summary of all available research in the field.

The applicability of the findings and recommendations from this review are restricted by the low quality of included studies: both reviewers judged the vast majority to be at high overall risk of bias. Nearly one-quarter of the included studies were published only as conference abstracts, and therefore not subjected to peer review. Data extraction was restricted to the information provided in the abstracts (no authors provided additional data upon request). These were limiting factors in our analysis, but we nonetheless felt it was important to

For peer review only - http://bmjopen.bmj.com/site/about/guidelines.xhtml

For peer review only

include these studies for completeness, especially given the paucity of evidence that exists in this field. A further justification for their inclusion is that half of the trials reported in conference abstracts never reach full publication, and positive trials are more likely to be published than negative ones,[67] which has the potential to skew the results of a review if they are omitted.

A further limitation of this review is that the majority of identified studies did not report substantive clinical outcomes such as maternal mortality, morbidity or harms. Without these, it is difficult to define properly the potential role of proposed interventions in clinical practice. The incidence of adverse maternal and neonatal outcomes, particularly in high resource settings, is low meaning adequately powering studies for real outcomes of interest is financially demanding. Therefore researchers often employ surrogate outcomes. Additionally, the range of outcomes reported in included studies was broad and inconsistent, with BP changes in particular being measured in a variety of different ways, further limiting the comparability of trials. Increasingly, core-outcome sets are being produced, with a view to trials reporting as standard, a minimum set of outcomes that are clinically meaningful and important to patients.[68] We hope in future this would enhance our ability to synthesize results from different studies to produce high-quality evidence. There is consensus about trying to move away from surrogate outcomes, for example time to BP control, as they cannot effectively substitute for clinically important outcomes. An important and clinically meaningful end point should measure how a patient feels, functions, or survives.

The body of evidence identified was substantially smaller than that underpinning antenatal hypertension management: eighteen studies (n=982), not restricted to RCTs, evaluated antihypertensive medications postpartum. Furthermore, the size of all but a few individual studies was small. In comparison, a Cochrane review (2014) evaluating antihypertensive medication for mild to moderate hypertension in pregnancy included 49 RCTs (n=4,723).[69] Moreover, the quantity and quality of evidence supporting the management of HDP is vastly less than that available for essential hypertension outside pregnancy, where individual RCTs commonly involve several thousand participants.[70]

This review demonstrates a lack of good quality evidence for postpartum hypertension management, emphasising the need for further RCTs directly comparing different antihypertensive agents, BP thresholds for medication adjustment and different models of care, with outcome measures other than postnatal readmissions. We believe the studies

For peer review only - http://bmjopen.bmj.com/site/about/guidelines.xhtml
For peer review only

examining uterine curettage justify further research to evaluate clinically meaningful outcomes and procedural risks. It might be pragmatic to confine this to curettage at caesarean section, given concerns regarding surgical intervention after vaginal birth: an additional anaesthetic is not required; infection risk is lowered within a sterile surgical field compared to the transcervical route, and curettage under direct vision limits perforation risk. This might be beneficial in women with severe preeclampsia where BP control during pregnancy has been challenging despite multiple medications.[55]

For peer review only

For peer review only - http://bmjopen.bmj.com/site/about/guidelines.xhtml

Acknowledgements

Thanks to Dr Ly-Mee Yu, Dr Helen Cotton and Dr Victoria E Cairns for their assistance with translation.

Funding

The research was funded by the National Institute for Health Research (NIHR) Collaboration for Leadership in Applied Health Research and Care Oxford at Oxford Health NHS Foundation Trust and via a Research Professorship awarded to RMcM (NIHR-RP-02-12-015). The views expressed are those of the author(s) and not necessarily those of the NHS, the NIHR or the Department of Health.

Details of ethical approval

No ethical approval was required for this project.

Contribution to authorship

AC drafted the protocol with JD and drafted and piloted the data extraction sheet. These were reviewed by RMcM, LP, KT, LM and PL. NR and AC wrote the search strategy, and the online searches were conducted by NR. AC and LP reviewed the search results independently and carried out the data extraction. This manuscript was drafted by AC and reviewed by RMcM, JD, LP, NR, KT, LM and PL. AC will be the guarantor.

For peer review only

For peer review only - http://bmjopen.bmj.com/site/about/guidelines.xhtml

References

1. Podymow T, August P. Postpartum course of gestational hypertension and preeclampsia. *Hypertens Pregnancy* 2010;29(3):294-300. doi: 10.3109/10641950902777747

2. Goel A, Maski MR, Bajracharya S, et al. Epidemiology and Mechanisms of De Novo and Persistent Hypertension in the Postpartum Period. *Circulation* 2015;132(18):1726-33. doi: 10.1161/CIRCULATIONAHA.115.015721.

3. Chames MC, Livingston JC, Ivester TS, et al. Late postpartum eclampsia: a preventable disease? *Am J Obstet Gynecol* 2002;186(6):1174-7. doi: 10.1067/mob.2002.123824

4. Kayem G, Kurinczuk JJ, Spark P, et al. Maternal and obstetric factors associated with delayed postpartum eclampsia: a national study population. *Acta Obstet Gyn Scand* 2011;90(9):1017-23. doi: 10.1111/j.1600-0412.2011.01218.x

5. Watson DL, Sibai BM, Shaver DC, et al. Late postpartum eclampsia: an update. *South Med J* 1983;76(12):1487-9.

6. Martin JN, Jr., Thigpen BD, Moore RC, et al. Stroke and severe preeclampsia and eclampsia: a paradigm shift focusing on systolic blood pressure. *Obstet Gynecol* 2005;105(2):246-54. doi: 10.1097/01.AOG.0000151116.84113.56

7. Berks D, Steegers EA, Molas M, et al. Resolution of hypertension and proteinuria after preeclampsia. *Obstet Gynecol* 2009;114(6):1307-14. doi: 10.1097/AOG.0b013e3181c14e3e

8. National Institute for Health and Care Excellence. NICE Clinical Guideline 107: Hypertension in pregnancy: the management of hypertensive disorders during pregnancy, 2011.

9. ACOG Task Force on Hypertension in Pregnancy. Hypertension in Pregnancy: American College of Obstetricians and Gynecologists, 2013.

10. Magee L, von Dadelszen P. Prevention and treatment of postpartum hypertension. *Cochrane Database Syst Rev* 2013(4) doi: 10.1002/14651858.CD004351.pub3

11. Moher D, Shamseer L, Clarke M, et al. Preferred reporting items for systematic review and meta-analysis protocols (PRISMA-P) 2015 statement. *Syst Rev* 2015;4(1) doi: 10.1186/2046-4053-4-1

For peer review only - http://bmjopen.bmj.com/site/about/guidelines.xhtml

12. McManus RJ, Wilson S, Delaney BC, et al. Review of the usefulness of contacting other experts when conducting a literature search for systematic reviews. *BMJ* 1998;317:1562-3.

13. Higgins JPT, Altman DG, Gøtzsche PC, et al. The Cochrane Collaboration's tool for assessing risk of bias in randomised trials. *BMJ* 2011;343:d5928. doi: 10.1136/bmj.d5928

14. Wells GA, Shea B, O'Connell D, et al. The Newcastle-Ottawa Scale (NOS) for assessing the quality of nonrandomised studies in meta-analyses [Available from: http://www.ohri.ca/programs/clinical_epidemiology/oxford.htm accessed 14/07/2017 2017.

15. Ragab A, Goda H, Raghib M, et al. Does immediate postpartum curettage of the endometrium accelerate recovery from preeclampsia-eclampsia? A randomized controlled trial. *Arch Gynecol Obstet* 2013;288(5):1035-8. doi: 10.1007/s00404-013-2866-0

16. Ascarelli MH, Johnson V, McCreary H, et al. Postpartum preeclampsia management with furosemide: a randomized clinical trial. *Obstet Gynecol* 2005;105(1):29-33.

17. Begum MR, Quadir E, Begum A, et al. Management of hypertensive emergencies of pregnancy by hydralazine bolus injection vs continuous drip-a comparative study. *MedGenMed* 2002;4(4)

18. Mallapur A, Renuka B, Katageri G, et al. Role of postpartum curettage in recovery of severe preeclampsia and eclampsia patients-a randomised controlled trial. *Int J Gynaecol Obstet* 2015;131(Suppl 5):E191.

19. Veena P, Perivela L, Raghavan SS. Furosemide in postpartum management of severe preeclampsia: A randomized controlled trial. *Hypertens Pregnancy* 2017;36(1):84-9. doi: 10.1080/10641955.2016.1239735

20. United Nations Department of Economic and Social Affairs. World economic situation and prospects 2016. New York, United States of America: United Nations, 2016.

21. Vermillion ST, Scardo JA, Newman RB, et al. A randomized, double-blind trial of oral nifedipine and intravenous labetalol in hypertensive emergencies of pregnancy. *Am J Obstet Gynecol* 1999;181(4):858-61.

22. Mabie WC, Gonzalez AR, Sibai BM, et al. A comparative trial of labetalol and hydralazine in the acute management of severe hypertension complicating pregnancy. *Obstet Gynecol* 1987;70(3, Part 1):328-33.

For peer review only - http://bmjopen.bmj.com/site/about/guidelines.xhtml

23. Hennessy A, Thornton CE, Makris A, et al. A randomised comparison of hydralazine and mini-bolus diazoxide for hypertensive emergencies in pregnancy: the PIVOT trial. *Aust N Z J Obstet Gynaecol* 2007;47(4):279-85. doi: 10.1111/j.1479-828X.2007.00738.x

24. Garden A, Davey DA, Dommisse J. Intravenous labetalol and intravenous dihydralazine in severe hypertension in pregnancy. *Clin Exp Hypertens B* 1982;1(2-3):371-83.

25. Gaisin IR, Iskchakova AS, Shilina LV. Indapamide in the management of post-partum hypertension: A randomized, case-control study. *Eur Heart J* 2013;34(Suppl 1):271.

26. York R, Brown LP, Samuels P, et al. A randomized trial of early discharge and nurse specialist transitional follow-up care of high-risk childbearing women. *Nurs Res* 1997;46(5):254-61.

27. Sharma KJ, Greene N, Kilpatrick SJ. Oral labetalol compared to oral extended release nifedipine for persistent postpartum hypertension: A randomized controlled trial. *Am J Obstet Gynecol* 2016;214(Suppl 1):S27-8.

28. Sharma KJ, Greene N, Kilpatrick SJ. Oral labetalol compared to oral nifedipine for postpartum hypertension: A randomized controlled trial. *Hypertens Pregnancy* 2017;36(1):44-7. doi: 10.1080/10641955.2016.1231317

29. Noronha Neto C, Maia SSB, Katz L, et al. Clonidine versus Captopril for Severe Postpartum Hypertension: A Randomized Controlled Trial. *PLoS One* 2017;12(1) doi: 10.1371/journal.pone.0168124

30. Amorim MMR, Noronha Neto C, Maia SB, et al. Clonidine compared with captopril for severe postpartum hypertension. *Obstet Gynecol* 2015;125(5 Suppl 1):42S.

31. Katz L, Noronha Neto C, Maia S, et al. Clonidine versus captopril for severe postpartum hypertension: A randomized controlled trial. *Pregnancy Hypertens* 2015;5(Suppl 1):29-30. doi: 10.1016/j.preghy.2014.10.061

32. Barton JR, Hiett AK, Conover WB. The use of nifedipine during the postpartum period in patients with severe preeclampsia. *Am J Obstet Gynecol* 1990;162(3):788-92.

33. Bibbo C, Celi A, Thomas AM, et al. Does the addition of a specialized postpartum clinic improve the care of women with preeclampsia? *Obstet Gynecol* 2014;123(5 Suppl 1):39S.

For peer review only - http://bmjopen.bmj.com/site/about/guidelines.xhtml

34. Sayin NC, Altundag G, Varol FG. Efficacy of alpha-methyldopa and nifedipine in the treatment of postpartum hypertension. [Turkish] Postpartum hipertansiyon tedavisinde alfametildopa ve nifedipinin etkinligi. *J Turk Ger Gynecol Assoc* 2005;6(2):118-22.

35. Vigil-De Gracia P, Ruiz E, Lopez JC, et al. Management of severe hypertension in the postpartum period with intravenous hydralazine or labetalol: a randomized clinical trial. *Hypertens Pregnancy* 2007;26(2):163-71. doi: 10.1080/10641950701204430

36. Palot M, Jakob L, Decaux J. Arterial hypertensions of labor and the post-partum period. [French] Les Hypertensions Arterielles Du Travail Et Du Post-Partum. *Rev Fr Gynecol Obstet* 1979;74(3):173-6.

37. Ilshat Gaisin IR, Iskchakova AS, Shilina LV. Control of cardiovascular risk factors with ursodeoxycholic acid and indapamide in postpreeclamptic nursing mothers: Results from a randomized, case-control 1-year study. *Eur J Prev Cardiol* 2014;21(Suppl 1):S120. doi: 10.1177/2047487314534585

38. Griffis KR, Jr., Martin JN, Jr., Palmer SM, et al. Utilization of hydralazine or alpha-methyldopa for the management of early puerperal hypertension. *Am J Perinatol* 1989;6(4):437-41.

39. Martin JN, Griffis KR, Martin RW, et al. Early puerperal hypertension management: hydralazine vs methyldopa. *Clin Exp Hypertens B* 1988;8(2):431.

40. Walss Rodriguez RJ, Villarreal Ordaz F. Management of severe pre-eclampsia in the puerperium. Comparative study of sublingual nifedipine and hydralazine. *Ginecol Obstet Mex* 1991;59:207-10.

41. Shumard K, Yoon J, Huang C, et al. Peripartum anti-hypertensive choice affects time to blood pressure control in treating hypertensive disorders of pregnancy. *Am J Obstet Gynecol* 2016;214(1 Suppl):S378.

42. Fidler J, Smith V, De Swiet M. A randomized study comparing timolol and methyldopa in hospital treatment of puerperal hypertension. *Br J Obstet Gynaecol* 1982;89(12):1031-4.

43. Krebs A. Experience with serpasil and luminal in the management of pregnancy-and puerperal toxemias. *Gynaecologia* 1956;141(4):255-60.

44. Krebs A. Tests of reserpine and phenobarbital in therapy of toxicosis of pregnancy and puerperium. *Geburtshilfe Frauenheilkd* 1956;16(5):410-22.

For peer review only - http://bmjopen.bmj.com/site/about/guidelines.xhtml

45. Amorim M, Katz L, Cursino T, et al. Postpartum furosemide for accelerating recovery in women with severe preeclampsia: A randomized clinical trial. *Int J Gynaecol Obstet* 2015;131(Suppl 5):E195.

46. Matthews G, Gornall R, Saunders NJ. A randomised placebo controlled trial of loop diuretics in moderate/severe pre-eclampsia, following delivery. *J Obstet Gynaecol* 1997;17(1):30-2. doi: 10.1080/01443619750114040

47. Shigemitsu A, Akasaka J, Shigetomi H, et al. Use of carperitide for postpartum diuresis of severe preeclampsia. *Pregnancy Hypertens* 2015;5(1):145. doi: 10.1016/j.preghy.2014.10.297

48. Weiner CP, Socol ML. Serotonin (5HT) receptor blockade in puerperal pre-eclampsia. *Clin Exp Hypertens B* 1982;1(2-3):195.

49. Weiner CP, Socol ML, Vaisrub N. Control of preeclamptic hypertension by ketanserin, a new serotonin receptor antagonist. *Am J Obstet Gynecol* 1984;149(5):496-500.

50. Montenegro R, Knuppel RA, Shah D, et al. The effect of serotonergic blockade in postpartum preeclamptic patients. *Am J Obstet Gynecol* 1985;153(2):130-4.

51. Hladunewich MA, Derby GC, Lafayette RA, et al. Effect of L-arginine therapy on the glomerular injury of preeclampsia: a randomized controlled trial. *Obstet Gynecol* 2006;107(4):886-95. doi: 10.1097/01.AOG.0000207637.01824.fe

52. Liu Z, Wang X, Yan N. Treatment of albuminuria in gestational hypertension puerpera in the severe preeclampeia stage by TCM therapy for stasis-removing and diuresis. *Zhongguo Zhong Xi Yi Jie He Za Zhi* 2009;29(3):222-4.

53. Barrilleaux PS, Martin JN, Jr., Klauser CK, et al. Postpartum intravenous dexamethasone for severely preeclamptic patients without hemolysis, elevated liver enzymes, low platelets (HELLP) syndrome: a randomized trial. *Obstet Gynecol* 2005;105(4):843-8.

54. Barrilleaux PS, Martin Jr J, Klauser C, et al. Adjunctive intravenous dexamethasone in patients with severe preeclampsia not complicated by HELLP syndrome. *Am J Obstet Gynecol* 2003;189(6 Suppl):S94.

For peer review only

For peer review only - http://bmjopen.bmj.com/site/about/guidelines.xhtml

55. Caughey AB. Editorial Comment: Does Immediate Postpartum Curettage of the Endometrium Accelerate Recovery From Preeclampsia–Eclampsia? A Randomized Controlled Trial. *Obstet Gynecol Surv* 2014;69(3):121-2. doi: 10.1097/ogx.0000000000000040

56. Hunter CA, Jr., Howard WF, McCormick CO, Jr. Amelioration of the hypertension of toxemia by postpartum curettage. *Am J Obstet Gynecol* 1961;81(5):884-9.

57. Gocmen A, Yayla M, Ceylan Erden A, et al. The effect of postpartum curettage in severe preeclampsia and eclampsia in recovery (Ağır preeklampsı ve eklampsi in iyileşmesinde postpartum kuretajin etkisi). *Perinatoloji Dergisi* 1996;4(1):24.

58. Salvatore CA, Carduz E, Ciccivizzo E, et al. Postpartum curettage in severe toxemia with and without premature loosening of the placenta. *Matern Infanc (Sao Paulo)* 1967;26(3):275-86.

59. Magann EF, Martin JN, Jr., Isaacs JD, et al. Immediate postpartum curettage: accelerated recovery from severe preeclampsia. *Obstet Gynecol* 1993;81(4):502-6.

60. Magann EF, Bass JD, Chauhan SP, et al. Accelerated recovery from severe preeclampsia: uterine curettage versus nifedipine. *J Soc Gynecol Investig* 1994;1(3):210-4.

61. Gomez LM, De la Vega GA, Ludmir J, et al. Immediate postpartum curettage accelerates clinical recovery in severe preeclampsia. *Obstet Gynecol* 2005;105(4 Suppl 4):111S.

62. Alkan A, Tugrul S, Oral O, et al. Effects of postpartum uterine curettage on maternal well-being in severe preeclamptic patients. *Clin Obstet Gynecol* 2006;33(1):55-8.

63. Webster J, Koch HF. Aspects of tolerability of centrally acting antihypertensive drugs. *J Cardiovasc Pharmacol* 1996;27(Suppl 3):S49-54.

64. Joint Formulary Committee. British National Formulary (BNF). 71 ed: BMJ Publishing Group Ltd and Royal Pharmaceutical Society 2016.

65. Tan LK, de Swiet M. The management of postpartum hypertension. *Br J Obstet Gynaecol* 2002;109(7):733-6.

66. Duley L, Gulmezoglu AM, Henderson-Smart DJ, et al. Magnesium sulphate and other anticonvulsants for women with pre-eclampsia. *Cochrane Database Syst Rev* 2010(11) doi: 10.1002/14651858.CD000025.pub2

For peer review only - http://bmjopen.bmj.com/site/about/guidelines.xhtml

67. Scherer RW, Langenberg P, von Elm E. Full publication of results initially presented in abstracts. *Cochrane Database Syst Rev* 2007(2) doi: 10.1002/14651858.MR000005.pub3

68. Khan K. The CROWN Initiative: journal editors invite researchers to develop core outcomes in women's health. *Br J Obstet Gynaecol* 2014;121(10):1181-2. doi: 10.1111/1471-0528.12929

69. Abalos E, Duley L, Steyn DW. Antihypertensive drug therapy for mild to moderate hypertension during pregnancy. *Cochrane Database Syst Rev* 2014(2) doi: 10.1002/14651858.CD002252.pub3

70. National Institute for Health and Care Excellence. NICE Clinical Guideline 127: Hypertension in adults: diagnosis and management, 2011.

For peer review only

For peer review only - http://bmjopen.bmj.com/site/about/guidelines.xhtml

Tables and figures

Figure 1: PRISMA Flowchart

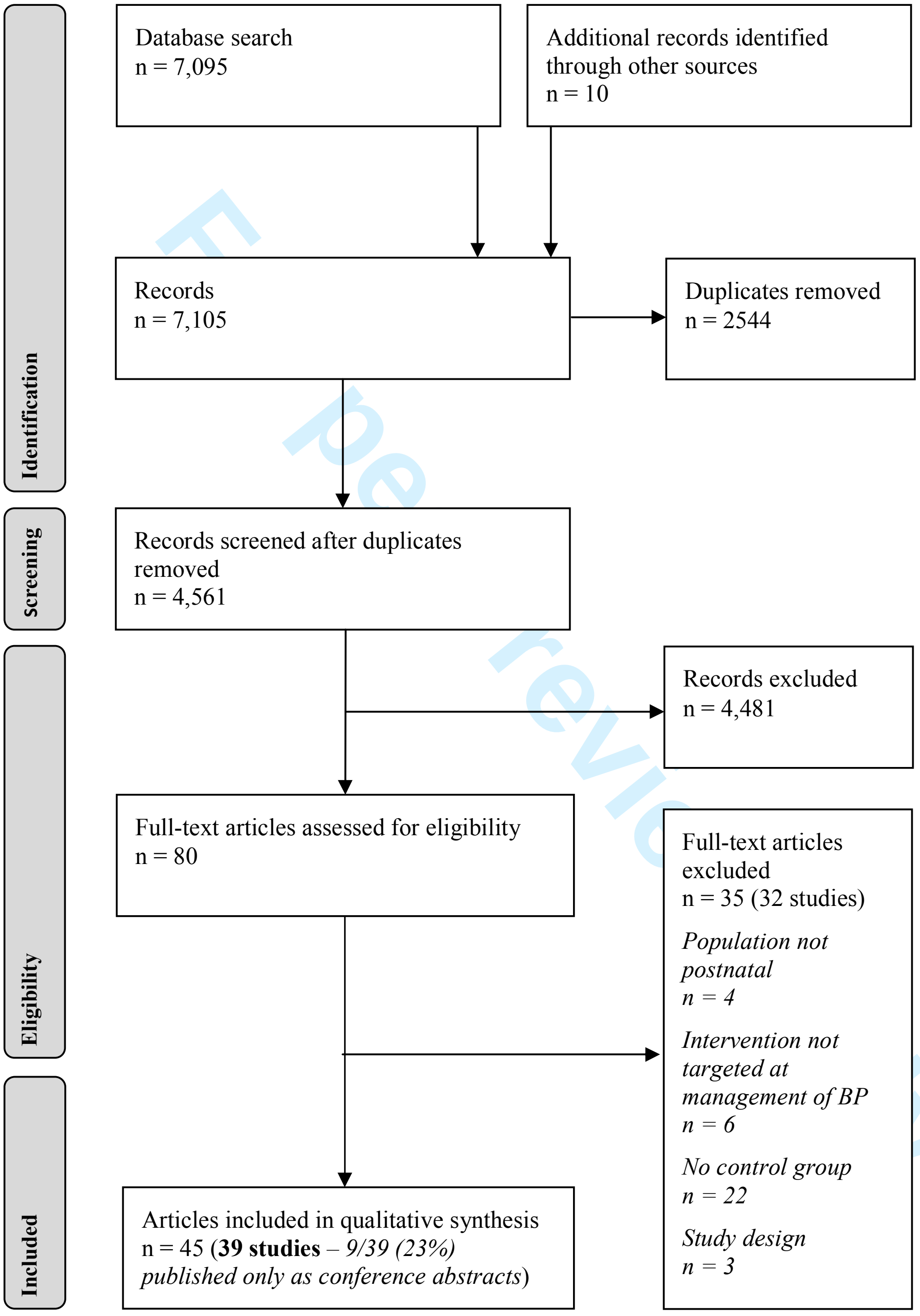

For peer review only - http://bmjopen.bmj.com/site/about/guidelines.xhtml

Table 1: Outcome measures

	Outcome measures	Timing
**Primary outcome(s)**	Maternal mortality<br>Maternal morbidity (ischaemic stroke, intracranial haemorrhage, eclamptic seizure; development of preeclampsia with severe features; postnatal complication requiring intervention)<br>Systolic blood pressure control<br>Diastolic blood pressure control<br>Mean arterial pressure control<br>Safety data (adverse events or maternal side effects)	Direct maternal deaths up to day 42 postpartum; later maternal deaths up to 1 year postpartum
**Secondary outcome(s)**	Critical care admission<br>Length of hospital stay following delivery<br>Postnatal readmission to secondary care<br>Antihypertensive medication requirement<br>Urine output<br>Laboratory values<br>Other as defined by study	

For peer review only

For peer review only - http://bmjopen.bmj.com/site/about/guidelines.xhtml

Table 2a: Primary outcome and safety data reporting in included studies (Antihypertensive medications, 18 studies)

Study ID	Intervention	Control	Primary outcome assessment					Safety data reporting	Results (for reported outcomes)
			Maternal mortality	Maternal morbidity	SBP control	DBP control	MAP control		
**CALCIUM CHANNEL BLOCKERS (3 studies)**									
Barton 1990[32]	Nifedipine (oral)	Placebo			🟡	🟡	🟢		SBP control: no significant difference.<br>DBP control: no significant difference.<br>MAP control: improved in intervention group (difference 6.3mmHg, p<0.05).
Vermillion 1999[21]	Nifedipine (oral)	Labetalol (IV bolus)			🟢	🟢		🟡	SBP control: improved in intervention group (difference in time to target BP 18.5 minutes, p=0.002).<br>DBP control: improved in intervention group (difference in time to target BP 18.5 minutes, p=0.002).<br>Safety: no significant difference. 1/25 intervention group became hypotensive.
Sayin 2005[34]	Nifedipine (oral)	Methyldopa (oral)	🟡		🟡	🟡			Maternal mortality: no significant difference.<br>SBP control: no significant difference.<br>DBP control: no significant difference.
**VASODILATORS (6 studies)**									
Palot 1979[36]	Hydralazine (IV infusion) plus furosemide (IV bolus)	Clonidine (IV) plus furosemide (IV bolus)		⚫					Maternal morbidity: no statistical analysis.
Griffis 1989[38 39]	Hydralazine (IM)	Methyldopa (IV bolus)					🟡	🟡	MAP control: no significant difference.<br>Safety: no significant difference. No side effects reported in either group.
Walss Rodriguez 1991[40]	Hydralazine (oral) plus nifedipine (oral, as required)	Nifedipine (oral, as required)			🟡	🟡			SBP control: no significant difference.<br>DBP control: no significant difference.
Begum 2002[17]	Hydralazine (IV bolus)	Hydralazine (IV infusion)				🟢		🟡	DBP control: improved in intervention group (difference in time to target DBP 121.1 minutes, p<0.001).<br>Safety: no significant difference. No side effects reported in either group.
Vigil de Gracia 2007[35]	Hydralazine (IV bolus)	Labetalol (IV bolus)	🟡	🟡	🟡	🟡		🟡	Maternal mortality: no significant difference.<br>Maternal morbidity: no significant difference.<br>SBP control: no significant difference.<br>DBP control: no significant difference.<br>Safety: no significant difference. Small numbers of side effects reported in both groups.
Hennessy 2007[23]	Diazoxide (IV bolus)	Hydralazine (IV bolus)			🟢	🟢			SBP control: improved in intervention group (difference in percentage achieving target BP 23%, p<0.01).<br>DBP control: improved in intervention group (difference in percentage achieving target BP 23%, p<0.01).
**BETA BLOCKERS (5 studies)**									
Garden 1982[24]	Labetalol (IV infusion)	Dihydralazine (IV infusion)				⚫		⚫	DBP control: no statistical analysis.<br>Safety: no statistical analysis. 1/6 intervention group developed bronchospasm. 4/6 control group developed tachycardia and 1/6 developed oliguria. 4/6 control group – drug stopped due to a precipitous fall of DBP to 40-50mmHg.

For peer review only

For peer review only - http://bmjopen.bmj.com/site/about/guidelines.xhtml

Study ID	Intervention	Control	Primary outcome assessment					Safety data reporting	Results (for reported outcomes)
			Maternal mortality	Maternal morbidity	SBP control	DBP control	MAP control		
Fidler 1982[42]	Timolol (oral)	Methyldopa (oral)			🟢	🟡		⚫	SBP control: improved in intervention group (difference 5.1mmHg, p<0.05).<br>DBP control: no significant difference.<br>Safety: no statistical analysis. 1/40 intervention group became disorientated. 1/40 control group became hypotensive and 1/40 became drowsy.
Mabie 1987[22]	Labetalol (IV bolus)	Hydralazine (IV bolus)					🔴	⚫	MAP control: improved in control group (difference 7.8mmHg (p 0.02).<br>Safety: no statistical analysis. 1/40 intervention group developed scalp tingling. 2/20 control group developed headaches.
Shumard 2016[41]	Labetalol (oral)	Nifedipine (oral)			🔴	🔴			SBP control: improved in control group (difference in time to achieve target BP 1 day, p=0.0031).<br>DBP control: improved in control group (difference in time to achieve target BP 1 day, p=0.0031).
Sharma 2017[27 28]	Labetalol (oral)	Nifedipine (oral)			🟡	🟡		🟢	SBP: no significant difference.<br>DBP: no significant difference.<br>Safety: No major side effects reported in either group. Minor side effects more commonly reported in control group (20% intervention, 48% control, p=0.04).
**THIAZIDES (2 studies)**									
Gaisin 2013[25]	Indapamide (oral)	Methyldopa (oral)			🟡	🟡		⚫	SBP control: no significant difference.<br>DBP control: no significant difference.<br>Safety: no statistical analysis, no details reported.
Gaisin 2014[37]	Indapamide (oral) plus ursodeoxycholic acid (oral)	Methyldopa (oral)			🟡	🟡		🟡	SBP control: no significant difference.<br>DBP control: no significant difference.<br>Safety: no significant difference. No adverse events reported in either group.
**INDOLE ALKALOIDS (1 study)**									
Krebs 1956[43 44]	Reserpine (oral or IM)	Phenobarbital			⚫	⚫		⚫	SBP control: no statistical analysis.<br>DBP control: no statistical analysis.<br>Safety: no statistical analysis. No adverse events reported in intervention group, no comment on control.
**CENTRALLY-ACTING ALPHA-AGONISTS (1 study)**									
Noronha Neto 2016[29-31]	Clonidine (oral)	Captopril (oral)	🟡	🟡	🟢	🟢		🟡	Maternal mortality: no significant difference.<br>Maternal morbidity: no significant difference.<br>SBP control: improved in intervention group (difference in number of episodes of high BP (1.4, p<0.08).<br>DBP: improved in intervention group (difference in number of episodes of high BP (1.4, p<0.08).<br>Safety: no significant difference. Adverse reactions 18.6% intervention, 28.8% control, p=NS.

For peer review only

For peer review only - http://bmjopen.bmj.com/site/about/guidelines.xhtml

Table 2b: Primary outcome and safety data reporting in included studies (Loop diuretics, other drugs, uterine curettage and organisation of care, 21 studies)

Study ID	Intervention	Control	Primary outcome assessment					Safety data reporting	Results (for reported outcomes)
			Maternal mortality	Maternal morbidity	SBP control	DBP control	MAP control		
**LOOP DIURETICS (4 studies)**									
Matthews 1997[46]	Furosemide (oral)	Placebo					🟡		MAP control: no significant difference.
Ascarelli 2005[16]	Furosemide (oral)	No intervention		🟡	🟡	🟡			Maternal morbidity: no significant difference.<br>SBP control: no significant difference.<br>DBP control: no significant difference.
Amorim 2015[45]	Furosemide (oral)	Placebo			🟢	🟢	🟢		SBP control: improved in intervention group (difference not stated, p<0.001).<br>DBP control: improved in intervention group (difference not stated, p<0.001).<br>MAP control: improved in intervention group (difference not stated, p<0.001).
Veena 2017[19]	Furosemide (oral) + nifedipine (oral)	Nifedipine (oral)		🟡	🟡	🟡	🟡		Maternal morbidity: no significant difference.<br>SBP control: no significant difference.<br>DBP control: no significant difference.<br>MAP control: no significant difference.
**OTHER DRUGS (7 studies)**									
**Selective 5-HT antagonists**									
Weiner 1982[48]	R41468 (intravenous infusion)	Placebo					🟢		MAP control: improved in intervention group (difference 25.6mmHg, p<0.001).
Weiner 1984[49]	Ketanserin (IV infusion)	Placebo			🟢	🟢	🟢	⚫	SBP control: improved in intervention group (difference in SBP decline 34mmHg, p<0.001).<br>DBP control: improved in intervention group (difference in DBP decline 27mmHg, p<0.001).<br>MAP control: improved in intervention group (difference not stated, p<0.001)<br>Safety: No statistical analysis. 3/20 intervention group experienced blurred vision: 1 of these was hypotensive (responded to hydration). 1/20 intervention group experienced mild euphoria.
Montenegro 1985[50]	Ketanserin (IV bolus +/- infusion)	Placebo			🟢	🟢	🟢		SBP control: improved in intervention group (absolute difference not stated, p<0.001).<br>DBP control: improved in intervention group (absolute difference not stated, p<0.001).<br>MAP control: improved in intervention group (absolute difference not stated, p<0.001).
**Alternative therapies**									
Hladunewich 2006[51]	L-arginine (oral or IV bolus)	Placebo			🟡	🟡	🟡	🟡	SBP control: no significant difference.<br>DBP control: no significant difference.<br>MAP control: no significant difference.<br>Safety: no significant difference. No adverse events reported in either group.
Liu 2009[52]	Shengkangbao (oral or IV bolus)	No intervention			🟡	🟡			SBP control: no significant difference.<br>DBP control: no significant difference.

For peer review only

For peer review only - http://bmjopen.bmj.com/site/about/guidelines.xhtml

Study ID	Intervention	Control	Primary outcome assessment					Safety data reporting	Results (for reported outcomes)
			Maternal mortality	Maternal morbidity	SBP control	DBP control	MAP control		
**Steroids**									
Barrilleaux 2005[53 54]	Dexamethasone (IV bolus)	Placebo					●		MAP control: no significant difference.
**Atrial natriuretic peptide**									
Shigemitsu 2015[47]	Carperitide (route not specified)	No intervention	●				●	●	Maternal mortality: no significant difference.<br>MAP control: no significant difference.<br>Safety: no significant difference. No adverse events reported in either group.
**UTERINE CURETTAGE (8 studies)**									
Salvatore 1967[58]	Uterine curettage	No intervention		●	●	●			Maternal morbidity: no statistical analysis.<br>SBP control: no statistical analysis.<br>DBP control: no statistical analysis.
Magann 1993[59]	Uterine curettage	No intervention					●	●	MAP control: improved in intervention group (difference at different time points to 24h postpartum 6-10mmHg, p<0.05).<br>Safety: no significant difference. No complications reported from intervention (follow-up to 7 weeks postpartum).
Magann 1994[60]	Uterine curettage	Nifedipine (oral) or no intervention					●<br>●	●	MAP control: no significant difference between intervention and oral nifedipine; improved in intervention group compared to no intervention (difference at 8-48h postpartum 9-13mmHg, p=0.0017).<br>Safety: no significant difference. No complications/side effects reported from interventions (follow-up to 7 weeks postpartum).
Gocmen 1996[57]	Uterine curettage	No intervention					●		MAP control: improved in intervention group (difference not stated, p=0.01).
Gomez 2005[61]	Uterine curettage	No intervention					●	●	MAP control: improved in intervention group (difference not stated, p<0.001).<br>Safety: no significant difference. No complications reported from intervention.
Alkan 2006[62]	Uterine curettage	No intervention					●	●	MAP control: improved in intervention group (difference 6.8mmHg, p<0.05).<br>Safety: No significant difference. No complications reported from intervention.
Ragab 2013[15]	Uterine curettage	No intervention	●	●			●		Maternal mortality: no significant difference.<br>Maternal morbidity: no statistical analysis.<br>MAP control: improved in intervention group (difference at 6h postpartum 12.3mmHg, P=0.02, difference at 24h postpartum 9.2mmHg, p=0.01)
Mallapur 2015[18]	Uterine curettage	No intervention					●		MAP control: improved in intervention group (difference at 4h postpartum 7.6mmHg, p<0.001).
**ORGANISATION OF CARE (2 studies)**									
York 1997[26]	Nurse specialist follow-up	No intervention							N/A
Bibbo 2014[33]	Specialist postpartum clinic	No intervention							N/A

For peer review only

For peer review only - http://bmjopen.bmj.com/site/about/guidelines.xhtml

Legend for Tables 2a&b

• = improved in intervention group; • = no significant difference; • = improved in control group; • = unclear

For primary outcome assessment where there was a significant difference between groups, the magnitude of the difference is reported; where any adverse events or side effects were reported this is presented

For peer review only

For peer review only - http://bmjopen.bmj.com/site/about/guidelines.xhtml

NUFFIELD DEPARTMENT OF PRIMARY CARE HEALTH SCIENCES

UNIVERSITY OF OXFORD

Appendix S1: Management of hypertensive disorders of pregnancy in the postpartum period: A systematic review protocol

Registration: PROSPERO CRD42015015527

http://www.crd.york.ac.uk/PROSPERO/display_record.asp?ID=CRD42015015527#.VL4ZI9KsWCk

Authors: Alexandra E Cairns, Louise Pealing, Nia Roberts, Richard J McManus
Corresponding author: Alexandra E Cairns, Nuffield Department of Primary Care Health Sciences, University of Oxford, New Radcliffe House, Radcliffe Observatory Quarter, Woodstock Road, Oxford, OX2 6GG
alexandra.cairns@phc.ox.ac.uk

Amendments: Protocol first published 22/12/2014 (version 1.0). Protocol amended (version 2.0 25/03/2015) to include all reporting items from the PRISMA-P 2015 checklist, and PROSPERO registration number.

Review funder: NIHR Collaborations for Leadership in Applied Health Research and Care (funding A Cairns' fellowship)

Review sponsor: University of Oxford

For peer review only

For peer review only - http://bmjopen.bmj.com/site/about/guidelines.xhtml

NUFFIELD DEPARTMENT OF PRIMARY CARE HEALTH SCIENCES
UNIVERSITY OF OXFORD

Abstract

Rationale: Hypertensive disorders of pregnancy (gestational hypertension and pre-eclampsia) are a leading cause of direct maternal death in the UK, and affect approximately 5-10% of pregnancies. Hypertensive disorders of pregnancy persist during the postpartum period, and complications can occur during this time.

Research question: How should hypertensive disorders of pregnancy be managed in the postnatal period to minimise harm to patients and optimise quality of life?

Objectives:

1. Organisation of care: how should blood pressure be monitored in women with hypertensive disorders of pregnancy in the postnatal period?
2. What blood pressure thresholds should be used for anti-hypertensive treatment initiation, adjustment and cessation in the postnatal period?
3. Which anti-hypertensive medication(s) should be used in the postnatal period?
4. What are the benefits and harms of other therapeutic interventions for women with hypertensive disorders of pregnancy in the postnatal period?

Search strategy: Medline and nine other electronic databases will be searched for articles published from inception until October 2014 using a search strategy designed to capture all the relevant literature concerning the management of hypertensive disorders of pregnancy in the postnatal period.

Study eligibility criteria:
Population: postnatal women with gestational hypertension or pre-eclampsia as defined by study
Intervention: therapeutic intervention for hypertensive disorders of pregnancy
Comparisons: another intervention, placebo or no intervention
Study design: RCT, prospective or retrospective cohort study or case-control study
Publication date: no restrictions
Language: no restrictions

Data management and extraction: Two reviewers will first review the titles of articles yielded by the search, and then the abstracts of articles of potential relevance. The full papers of potentially eligible papers will be assessed, and data extracted independently by the two reviewers using a data extraction sheet. Differences in study selection and data extraction will be resolved by discussion.

Assessment of methodological quality: This will be done using the Cochrane Collaboration's tool for assessing risk of bias in randomised trials, and for the assessment of bias in cohort and case-control studies we will use the Newcastle-Ottawa quality assessment scales.

Systematic review registration: This systematic review is registered with PROSPERO (International prospective register of systematic reviews).

For peer review only

For peer review only - http://bmjopen.bmj.com/site/about/guidelines.xhtml

NUFFIELD DEPARTMENT OF PRIMARY CARE HEALTH SCIENCES

Rationale

Definitions

The National Institute for Health and Clinical Excellence (NICE) defines gestational hypertension as new-onset raised blood pressure (≥ 140/90mmHg) beyond 20 weeks gestation. NICE defines pre-eclampsia as new-onset raised blood pressure (≥ 140/90mmHg) together with new-onset significant proteinuria (≥ 300mg/24hr), beyond 20 weeks gestation (1).

The International Society for the Study of Hypertension in Pregnancy (ISSHP) defines pre-eclampsia as new-onset raised blood pressure (as defined by NICE) in association with one of new-onset significant proteinuria (as defined by NICE), maternal organ dysfunction or uteroplacental insufficiency (2).

Epidemiology

Hypertensive disorders of pregnancy remain the second commonest direct cause of maternal death in the USA (3). Until recently this has also been the case in the UK (CMACE 2006-8)(4), but the most recent Confidential Enquiry into maternal deaths showed that for the triennium 2009-11, pre-eclampsia and eclampsia was the fourth commonest cause of direct death (behind thrombosis, genital tract sepsis and haemorrhage), with a rate of 0.42 deaths per 100,000 maternities (5).

A recent population-based retrospective study in the United States found the rate of pre-eclampsia to be 3.4%. This study showed a slight, but significant increase, in the rates of both mild, and to a greater extent, severe pre-eclampsia over the period studied (1980-2010) (6).

Reviews of the literature, and national guidelines, quote rates of gestational hypertension between 6% (7) and 15% (8). A retrospective study using data from the National Hospital Discharge Survey in the United States (1987-2004) demonstrated an incidence of 30.6 cases of gestational hypertension per 1000 deliveries in 2003-2004 (3.1%) (9). In a well-designed large randomised controlled trial assessing preventative strategies for hypertensive disorders of pregnancy in low risk, nulliparous women the incidence of gestational hypertension across both groups was 6% (10).

Physiology of blood pressure in pregnancy and postpartum

As a result of a significant decrease in systemic vascular resistance (as early as 5 weeks gestation) (11) there is a decrease in arterial pressures from early in the first trimester. Arterial pressures reach a nadir in the second trimester, and then begin to rise in the third trimester, before reaching near-preconception levels in the postnatal period (12).

For peer review only - http://bmjopen.bmj.com/site/about/guidelines.xhtml

NUFFIELD DEPARTMENT OF PRIMARY CARE HEALTH SCIENCES
UNIVERSITY OF OXFORD

Figure 1: Serial blood pressures before, during and after pregnancy (reproduced from the data of Mahendru et al. 2014) (12)

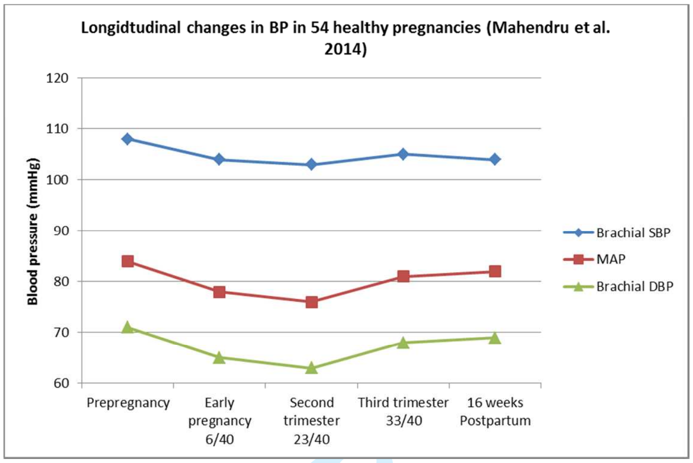

In gestational hypertension and pre-eclampsia the normal pregnancy-induced vasodilatation is reversed. In untreated women with pre-eclampsia significant increases in systemic vascular resistance are seen and result in elevation of blood pressure (13).

Hypertensive disorders of pregnancy in the postpartum period

There has been considerable focus on blood pressure control during pregnancy, especially with respect to pregnancy outcome. However, it is recognised that hypertensive disorders of pregnancy do persist during the postpartum period, and that complications can occur during this time. A small retrospective observational study published in 1987 looked at 67 women with moderate-severe pre-eclampsia: there was often an initial decrease in blood pressure after delivery, but this was followed by a rise to hypertensive levels in many women. In 50% of cases the blood pressure was 150/100mmHg or higher on day 5 after birth. The authors recommended continuing blood pressure monitoring and treatment in the postpartum period for women with a diagnosis of pre-eclampsia (14).

Most women with hypertensive disorders of pregnancy will be treatment-free by 3 months postpartum. In women whose blood pressure normalised after delivery the mean time to normalisation in a retrospective cohort study of 62 women was 5.4 weeks (15). This rapidly changing blood pressure, with shifting medication requirement, poses an additional challenge in terms of how best to manage this down-titration.

Approximately one third of eclamptic seizures occur postpartum, and studies suggest that over half of these seizures occur more than 48 hours after birth. Chames et al. (2002) highlight the importance of education of women and clinicians regarding prodromal symptoms of eclampsia in the postnatal

For peer review only - http://bmjopen.bmj.com/site/about/guidelines.xhtml

period (16). A case series published in 2005 of patients who sustained a stroke in association with severe pre-eclampsia or eclampsia, showed that more than half (57%) of these strokes occurred in the postpartum period (17).

Current guidelines

NICE guidelines highlight that very few clinical studies have addressed the management of blood pressure postpartum, and in practice clinical care is typically to continue antepartum antihypertensive medication and monitor blood pressure in the community with a focus on prevention of over-treatment.

NICE recommend frequency of monitoring in the postnatal period for both pre-eclampsia and gestational hypertension. The guidelines also stipulate thresholds for considering increasing or starting anti-hypertensive medication during this period (150/100 mmHg), and for reduction or stopping anti-hypertensive medication (consider at < 140/90 mmHg, and reduce at < 130/80 mmHg) (1).

Research question

How should hypertensive disorders of pregnancy be managed in the postnatal period to minimise harm to patients and optimise quality of life?

Objectives

The aim is to establish what evidence exists to guide the optimal approach to management of gestational hypertension and pre-eclampsia in the postnatal period. We want to address the specific sub-questions:

1. Organisation of care: how should blood pressure be monitored in women with hypertensive disorders of pregnancy in the postnatal period?
2. What blood pressure thresholds should be used for anti-hypertensive treatment initiation, adjustment and cessation in the postnatal period?
3. Which anti-hypertensive medication(s) should be used in the postnatal period?
4. What are the benefits and harms of other therapeutic interventions for women with hypertensive disorders of pregnancy in the postnatal period?

Information sources and search strategy

The systematic review of 'management of hypertensive disorders of pregnancy in the postpartum period' will be conducted in line with the PRISMA statement (18). Completion of a systematic review

For peer review only - http://bmjopen.bmj.com/site/about/guidelines.xhtml

is an iterative process, and it may be that modifications to the original review protocol are required during its conduct.

A search strategy designed to capture all the relevant literature concerning the management of hypertensive disorders of pregnancy in the postnatal period will be developed by an experienced trial search co-ordinator. Potentially relevant studies will be identified following screening of title and abstract of studies captured by the search and full text assessed for suitability.

Resources to be searched from inception to October 2014:

- Medline (Appendix 3) and 9 other electronic databases
- Trial registers (ClinicalTrials.gov; Current Controlled Trials; WHO; PROSPERO)
- Meta Search Engines
- Hand searches of reference lists
- Citation searching on Scopus and Web of Science
- Related articles search on PubMed
- Contact with authors and professional bodies / organisations: Experts in this field will be contacted for their recommendations of potentially relevant citations (19)

Study eligibility criteria

INCLUSION CRITERIA

Population: postnatal women with hypertensive disorders of pregnancy (gestational hypertension or pre-eclampsia).

Intervention: therapeutic intervention for management of hypertensive disorders of pregnancy

Comparisons: other intervention, placebo or no intervention

Study design: randomised controlled trial, cohort study (prospective and retrospective) or case-control study; human studies only

Publication Date: no restrictions

Language: no restrictions

EXCLUSION CRITERIA

Exclude report / study if **any** exclusion criteria fulfilled:

Population: antenatal or intrapartum women with hypertensive disorders of pregnancy; end-organ complications of pre-eclampsia (eclampsia, renal failure, HELLP syndrome)

For peer review only - http://bmjopen.bmj.com/site/about/guidelines.xhtml

Intervention: treatment of HELLP syndrome (haemolysis, elevated liver enzymes and low platelets); prevention or management of eclampsia; prevention of postpartum hypertension; choice of anaesthetic or sedative in pre-eclampsia; observational studies

Comparisons: no control group

Study design: guidelines, reviews, expert opinions, letters, commentaries, audits, case series and case reports excluded; animal studies

Data extraction

Two reviewers (AC and LP) will screen the titles and abstracts of articles yielded by the search against the eligibility criteria. Discrepancies will be resolved by consensus before determining the list of full papers for review. The reports will be screened independently by the two reviewers, and discrepancies will be resolved by discussion before deciding which papers to include in the review.

Data from included studies will be extracted independently by the two reviewers using a piloted and standardised data extraction sheet. Differences in data extraction will be resolved by discussion.

In the event that there is more than one report published about a single study: the reports will be reviewed separately but the data from that study grouped in our analysis, and the primary reference will be used.

In the event that data is missing from a report (for example the sole publication is a conference abstract) we will contact the authors directly to request further detail.

The study characteristics (study size, population, setting, study design, methodology, intervention, controls if applicable, outcome measures, and follow up period) will be recorded and reported.

Data synthesis

The data extracted will be aggregate.

Due to the heterogeneous nature of the outcomes reported in these studies a narrative synthesis is planned.

For trials where the population study is peripartum (i.e. a mixture of antepartum, intrapartum and postpartum) we will extract the data for the postpartum women and analyse this. If this is not feasible from the reported data then we will contact the study authors to request the data for this subgroup.

Outcomes

The results of all clinically relevant outcomes in hypertensive disorders of pregnancy that would be important to clinicians and patients will be extracted and reported.

The main outcomes we are interested in are listed in table 1 below:

For peer review only - http://bmjopen.bmj.com/site/about/guidelines.xhtml

NUFFIELD DEPARTMENT OF PRIMARY CARE HEALTH SCIENCES

Table 1

	Outcome measures	Timing
**Primary outcome(s)**	Maternal mortality<br>Major maternal morbidity (ischaemic stroke, intracranial haemorrhage, eclamptic seizure)<br>Systolic blood pressure control<br>Diastolic blood pressure control<br>Mean arterial pressure control	Direct maternal deaths upto day 42 postpartum; later maternal deaths upto 1 year postpartum
**Secondary outcome(s)**	Critical care admission<br>Postnatal readmission to secondary care<br>Length of hospital stay following delivery<br>Anti-hypertensive medication requirement<br>Maternal side effects of intervention<br>Development of pre-eclampsia with severe features<br>Postnatal complication requiring intervention<br>Urine output<br>Laboratory values	

Assessment of methodological quality

We will assess the risk of bias in each study. For randomised trials this will be done using the Cochrane Collaboration's tool for assessing risk of bias in randomised trials (Appendix 1, Table 2) (20). For each study the key domains will be identified, and then an overall assessment of bias within each trial made, according to the guidance published by the Cochrane Collaboration (Appendix 1, Table 2).

For the assessment of bias in cohort and case-control studies we will use the Newcastle-Ottawa quality assessment scales (Appendix 2, Tables 4 and 5) (21).

We will make a global assessment of bias across trials, based on the guidance from the Cochrane Collaboration (Appendix 1, Table 3):

- EITHER Most information is from trials at low risk of bias;
- OR most information is from trials at low or unclear risk of bias;
- OR the proportion of information from trials at high risk of bias is sufficient to affect the interpretation of results

For peer review only - http://bmjopen.bmj.com/site/about/guidelines.xhtml

NUFFIELD DEPARTMENT OF PRIMARY CARE HEALTH SCIENCES

Discussion

A Cochrane Review (2013) addresses the question of 'prevention and treatment of postpartum hypertension'. This only includes randomised controlled trials (9 in total), and does not address the issue of monitoring blood pressure during this period (22). Given the paucity of evidence cited in this area we believe there is a place for a review looking at all available evidence for the optimal approach to management of hypertensive disorders of pregnancy in the postpartum period.

Conflicts of interest

Neither AC nor LP have any conflicts of interest.

For peer review only

For peer review only - http://bmjopen.bmj.com/site/about/guidelines.xhtml

NUFFIELD DEPARTMENT OF PRIMARY CARE HEALTH SCIENCES

UNIVERSITY OF OXFORD

References

1. National Institute for Health and Care Excellence. NICE Clinical Guideline 107: Hypertension in pregnancy: the management of hypertensive disorders during pregnancy. 2011.

2. Tranquilli AL, Dekker G, Magee L, Roberts J, Sibai BM, Steyn W, et al. The classification, diagnosis and management of the hypertensive disorders of pregnancy: A revised statement from the ISSHP. Pregnancy Hypertension: An International Journal of Women's Cardiovascular Health. 2014;4(2):97-104.

3. American College of Obstetricians and Gynecologists. Diagnosis and management of preeclampsia and eclampsia. ACOG Practice Bulletin. ACOG Practice Bulletin No. 33: 2002.

4. Cantwell R, Clutton-Brock T, Cooper G, Dawson A, Drife J, Garrod D, et al. Saving Mothers' Lives: Reviewing maternal deaths to make motherhood safer: 2006-2008. The Eighth Report of the Confidential Enquiries into Maternal Deaths in the United Kingdom. BJOG : an international journal of obstetrics and gynaecology. 2011;118 Suppl 1:1-203.

5. Knight M, Kenyon S, Brocklehurst P, Neilson J, Shakespeare J, Kurinczuk J. Saving Lives, Improving Mothers' Care (MBRRACE-UK). 2014.

6. Ananth CV, Keyes KM, Wapner RJ. Pre-eclampsia rates in the United States, 1980-2010: age-period-cohort analysis. Bmj. 2013;347:f6564.

7. Yoder SR, Thornburg LL, Bisognano JD. Hypertension in pregnancy and women of childbearing age. The American journal of medicine. 2009;122(10):890-5.

8. National Institute for Health and Care Excellence. NICE Clinical Guideline 107: Hypertension in pregnancy: Costing report. Implementing NICE guidance. 2010.

9. Wallis AB, Saftlas AF, Hsia J, Atrash HK. Secular trends in the rates of preeclampsia, eclampsia, and gestational hypertension, United States, 1987-2004. American journal of hypertension. 2008;21(5):521-6.

10. Sibai BM, Caritis SN, Thom E, Klebanoff M, McNellis D, Rocco L, et al. Prevention of preeclampsia with low-dose aspirin in healthy, nulliparous pregnant women. The National Institute of Child Health and Human Development Network of Maternal-Fetal Medicine Units. The New England journal of medicine. 1993;329(17):1213-8.

11. Chapman AB, Abraham WT, Zamudio S, Coffin C, Merouani A, Young D, et al. Temporal relationships between hormonal and hemodynamic changes in early human pregnancy. Kidney international. 1998;54(6):2056-63.

12. Mahendru AA, Everett TR, Wilkinson IB, Lees CC, McEniery CM. A longitudinal study of maternal cardiovascular function from preconception to the postpartum period. Journal of hypertension. 2014;32(4):849-56.

13. Visser W, Wallenburg HC. Central hemodynamic observations in untreated preeclamptic patients. Hypertension. 1991;17(6 Pt 2):1072-7.

14. Walters BN, Walters T. Hypertension in the puerperium. Lancet. 1987;2(8554):330.

15. Podymow T, August P. Postpartum course of gestational hypertension and preeclampsia. Hypertension in pregnancy. 2010;29(3):294-300.

16. Chames MC, Livingston JC, Ivester TS, Barton JR, Sibai BM. Late postpartum eclampsia: a preventable disease? American journal of obstetrics and gynecology. 2002;186(6):1174-7.

17. Martin JN, Jr., Thigpen BD, Moore RC, Rose CH, Cushman J, May W. Stroke and severe preeclampsia and eclampsia: a paradigm shift focusing on systolic blood pressure. Obstetrics and gynecology. 2005;105(2):246-54.

18. Moher D, Liberati A, Tetzlaff J, Altman DG, Group P. Preferred reporting items for systematic reviews and meta-analyses: the PRISMA statement. PLoS medicine. 2009;6(7):e1000097.

19. McManus RJ, Wilson S, Delaney BC, Fitzmaurice DA, Hyde CJ, Tobias RS, et al. Review of the usefulness of contacting other experts when conducting a literature search for systematic reviews. Bmj. 1998;317(7172):1562-3.

For peer review only - http://bmjopen.bmj.com/site/about/guidelines.xhtml

NUFFIELD DEPARTMENT OF PRIMARY CARE HEALTH SCIENCES

20. Higgins JPT, Altman DG, Gøtzsche PC, Jüni P, Moher D, Oxman AD, et al. The Cochrane Collaboration's tool for assessing risk of bias in randomised trials2011 2011-10-18 10:55:48.
21. Wells GA, Shea B, O'Connell D, J. P, V. W, M. L, et al. The Newcastle-Ottawa Scale (NOS) for assessing the quality of nonrandomised studies in meta-analyses.
22. Magee L, von Dadelszen P. Prevention and treatment of postpartum hypertension. The Cochrane database of systematic reviews. 2013;4:CD004351.

For peer review only

For peer review only - http://bmjopen.bmj.com/site/about/guidelines.xhtml

NUFFIELD DEPARTMENT OF PRIMARY CARE HEALTH SCIENCES

UNIVERSITY OF OXFORD

Appendix 1

Table 2: Cochrane Collaboration's tool for assessing risk of bias (*adapted from Higgins and Altman*)(20)

**Bias domain**	**Source of bias**	**Support for judgment**	**Review authors' judgment** (assess as low, unclear or high risk of bias)
**Selection bias**	Random sequence generation	Describe the method used to generate the allocation sequence in sufficient detail to allow an assessment of whether it should produce comparable groups	Selection bias (biased allocation to interventions) due to inadequate generation of a randomised sequence
	Allocation concealment	Describe the method used to conceal the allocation sequence in sufficient detail to determine whether intervention allocations could have been foreseen before or during enrolment	Selection bias (biased allocation to interventions) due to inadequate concealment of allocations before assignment
**Performance bias**	Blinding of participants and personnel*	Describe all measures used, if any, to blind trial participants and researchers from knowledge of which intervention a participant received. Provide any information relating to whether the intended blinding was effective	Performance bias due to knowledge of the allocated interventions by participants and personnel during the study
**Detection bias**	Blinding of outcome assessment*	Describe all measures used, if any, to blind outcome assessment from knowledge of which intervention a participant received. Provide any information relating to whether the intended blinding was effective	Detection bias due to knowledge of the allocated interventions by outcome assessment
**Attrition bias**	Incomplete outcome data*	Describe the completeness of outcome data for each main outcome, including attrition and exclusions from the analysis. State whether attrition and exclusions were reported, the numbers in each intervention group (compared with total randomised participants), reasons for attrition or exclusions where reported, and any re-inclusions in analyses for the review	Attrition bias due to amount, nature, or handling of incomplete outcome data
**Reporting bias**	Selective reporting	State how selective outcome reporting was examined and what	Reporting bias due to selective

For peer review only - http://bmjopen.bmj.com/site/about/guidelines.xhtml

		was found	outcome reporting
**Other bias**	Anything else, ideally Pre-specified	State any important concerns about bias not covered in the other domains in the tool	Bias due to problems not covered elsewhere
*Assessments should be made for each main outcome or class of outcomes.			

Table 3: Approach to formulating summary assessments of risk of bias for each important outcome (across domains) within and across trials (*adapted from Higgins and Altman*)(20)

Risk of bias	Interpretation	Within a trial	Across trials
**Low risk of bias**	Bias, if present, is unlikely to alter the results seriously	Low risk of bias for all key domains	Most information is from trials at low risk of bias
**Unclear risk of bias**	A risk of bias that raises some doubt about the results	Low or unclear risk of bias for all key domains	Most information is from trials at low or unclear risk of bias
**High risk of bias**	Bias may alter the results seriously	High risk of bias for one or more key domains	The proportion of information from trials at high risk of bias is sufficient to affect the interpretation of results

For peer review only

For peer review only - http://bmjopen.bmj.com/site/about/guidelines.xhtml

NUFFIELD DEPARTMENT OF PRIMARY CARE HEALTH SCIENCES
UNIVERSITY OF OXFORD

Appendix 2

Table 4: Newcastle-Ottawa quality assessment scale case control studies(21)

A study can be awarded a maximum of one star for each numbered item within the Selection and Exposure categories. A maximum of two stars can be given for Comparability.

**Selection**	Is the case definition adequate?	a) Yes, with independent validation ✵<br>b) Yes, e.g. record linkage or based on self-reports<br>c) No description
	Representativeness of the cases	a) Consecutive or obviously representative series of cases ✵<br>b) Potential for selection biases not stated
	Selection of controls	a) Community controls ✵<br>b) Hospital controls<br>c) No description
	Definition of controls	a) No history of disease (endpoint) ✵<br>b) No description of source
**Comparability**	Comparability of cases and controls on the basis of the design or analysis	a) Study controls for <<_>> (select the post important factor) ✵<br>b) Study controls for any additional factor ✵
**Exposure**	Ascertainment of exposure	a) Secure records (e.g. surgical records) ✵<br>b) Structured interview where blind to case/control status ✵<br>c) Interview not blinded to case/control status<br>d) Written self-report or medical record only<br>e) No description
	Same method of ascertainment for cases and controls	a) Yes ✵<br>b) No
	Non-response rate	a) Same rate for both groups ✵<br>b) Non-respondents described<br>c) Rate different and no designation

Table 5: Newcastle-Ottawa quality assessment scale cohort studies(21)

A study can be awarded a maximum of one star for each numbered item within the Selection and Outcome categories. A maximum of two stars can be given for Comparability.

**Selection**	Representativeness of the exposed cohort	a) Truly representative of the average <<_>> (describe) in the community ✵<br>b) Somewhat representative of the average <<_>> (describe) in the community ⍰<br>c) Selected group of users e.g. nurses, volunteers<br>d) No description of the derivation of the cohort
	Selection of the non-exposed cohort	a) Drawn from the same community as the exposed cohort ✵<br>b) Drawn from a different source<br>c) No description of the derivation of the non-exposed cohort
	Ascertainment of exposure	a) Secure record (e.g. surgical records) ✵<br>b) Structured interview ✵<br>c) Written self-report<br>d) No description

For peer review only - http://bmjopen.bmj.com/site/about/guidelines.xhtml

	Demonstration that the outcome of interest was not present at start of study	a) Yes ✵<br>b) No
**Comparability**	Comparability of cases and controls on the basis of the design or analysis	a) Study controls for <<_>> (select the post important factor) ✵<br>b) Study controls for any additional factor ✵
**Outcome**	Assessment of outcome	a) Independent blind assessment ✵<br>b) Record linkage ✵<br>c) Self-report<br>d) No description
	Was follow-up long enough for outcomes to occur	a) Yes (select an adequate follow up period for outcome of interest) ✵<br>b) No
	Adequacy of follow-up of cohorts	a) Complete follow-up – all subjects accounted for ✵<br>b) Subjects lost to follow-up unlikely to introduce bias: >_ _ % (select an adequate %) follow-up rate, or description provided of those lost) ✵<br>c) Follow-up rate < _ _ % (select an adequate %) and no description of those lost<br>d) No statement

For peer review only - http://bmjopen.bmj.com/site/about/guidelines.xhtml

NUFFIELD DEPARTMENT OF PRIMARY CARE HEALTH SCIENCES

UNIVERSITY OF OXFORD

Appendix 3: Medline search strategy

# ▼	Searches	Results
1	Pregnancy/ and Hypertension/	9226
2	exp Hypertension, Pregnancy-Induced/	29022
3	((pregnan* or gestation* or maternal or prenatal or pre-natal or antenatal or ante-natal or antepart* or ante-part* or obstetric*) and (hypertens* or blood pressure or bp or dbp or sbp or diastolic or systolic)).ti.	6787
4	((pregnan* or gestation* or maternal or prenatal or pre-natal or antenatal or ante-natal or antepart* or ante-part* or obstetric*) adj3 (hypertens* or blood pressure or bp or dbp or sbp or diastolic or systolic)).ti,ab.	12434
5	(eclamp* or preeclamp* or pre-eclamp* or hellp).ti,ab.	25194
6	1 or 2 or 3 or 4 or 5	46611
7	Postnatal Care/	4044
8	Aftercare/	6684
9	Postpartum Period/ and Maternal Health Services/	126
10	exp Puerperal Disorders/ and Maternal Health Services/	196
11	Postpartum period/ and (exp Antihypertensive agents/ or exp calcium channel blockers/ or exp Angiotensin-Converting Enzyme Inhibitors/ or exp Adrenergic beta-Antagonists/ or exp Diuretics/)	187
12	exp Puerperal disorders/ and (exp Antihypertensive agents/ or exp calcium channel blockers/ or exp Angiotensin-Converting Enzyme Inhibitors/ or exp Adrenergic beta-Antagonists/ or exp Diuretics/)	237
13	Postpartum period/ and exp Curettage/	30
14	exp Puerperal disorders/ and exp Curettage/	118
15	Postpartum period/ and hypertension/dt, th	33
16	exp Puerperal disorders/ and hypertension/dt, th	54
17	exp Puerperal disorders/dt, th	6408
18	((postnatal or post-natal or postpart* or post-part* or puerper*) adj5 (care or healthcare or service* or program* or scheme* or intervention*)).ti,ab.	4407
19	((postnatal or post-natal or postpart* or post-part* or puerper*) adj5 (clinic? or unit? or visit* or referral? or appointment?)).ti,ab.	1491
20	((postnatal or post-natal or postpart* or post-part* or puerper*) adj5 (manage* or treat* or therap* or medication? or recovery)).ti,ab.	7287
21	((postnatal or post-natal or postpart* or post-part* or puerper*) adj5 (antihypertens* or anti-hypertens* or calcium channel block* or beta block* or b block* or ace inhibitor* or angiotensin converting enzyme inhibitor* or diuretic*)).ti,ab.	41
22	((postnatal or post-natal or postpart* or post-part* or puerper*) adj5 (evaluat* or assess* or screen* or diagnos* or monitor* or follow up or supervis*)).ti,ab.	7562
23	((postnatal or post-natal or postpart* or post-part* or puerper*) adj5 curet*).ti,ab.	82
24	(postnatal or post-natal or postpart* or post-part* or puerper*).ti.	41491
25	7 or 8 or 9 or 10 or 11 or 12 or 13 or 14 or 15 or 16 or 17 or 18 or 19 or 20 or 21 or 22 or 23 or 24	64775

For peer review only - http://bmjopen.bmj.com/site/about/guidelines.xhtml

NUFFIELD DEPARTMENT OF PRIMARY CARE HEALTH SCIENCES

26	6 and 25	1896
27	((postnatal or post-natal or postpart* or post-part* or puerper*) and (hypertens* or blood pressure)).ti.	270
28	26 or 27	1990
29	exp animals/ not humans.sh.	4079856
30	(rat or rats or rodent? or mice or mouse or cow or cows or cattle or calf or calves or ewe? or sheep or goat or ruminant? or pig or pigs or minipig? or chicken? or horse or horses or murine or bovine or ovine or porcine or animal?).ti.	1682619
31	29 or 30	4373527
32	28 not 31	1881

For peer review only

For peer review only - http://bmjopen.bmj.com/site/about/guidelines.xhtml

Appendix S2: PRISMA 2009 Checklist

Section/topic	#	Checklist item	Reported on page #
**TITLE**			
Title	1	Identify the report as a systematic review, meta-analysis, or both.	1
**ABSTRACT**			
Structured summary	2	Provide a structured summary including, as applicable: background; objectives; data sources; study eligibility criteria, participants, and interventions; study appraisal and synthesis methods; results; limitations; conclusions and implications of key findings; systematic review registration number.	2
**INTRODUCTION**			
Rationale	3	Describe the rationale for the review in the context of what is already known.	5
Objectives	4	Provide an explicit statement of questions being addressed with reference to participants, interventions, comparisons, outcomes, and study design (PICOS).	5-6
**METHODS**			
Protocol and registration	5	Indicate if a review protocol exists, if and where it can be accessed (e.g., Web address), and, if available, provide registration information including registration number.	6
Eligibility criteria	6	Specify study characteristics (e.g., PICOS, length of follow-up) and report characteristics (e.g., years considered, language, publication status) used as criteria for eligibility, giving rationale.	6
Information sources	7	Describe all information sources (e.g., databases with dates of coverage, contact with study authors to identify additional studies) in the search and date last searched.	6
Search	8	Present full electronic search strategy for at least one database, including any limits used, such that it could be repeated.	Appendix S1
Study selection	9	State the process for selecting studies (i.e., screening, eligibility, included in systematic review, and, if applicable, included in the meta-analysis).	6
Data collection process	10	Describe method of data extraction from reports (e.g., piloted forms, independently, in duplicate) and any processes for obtaining and confirming data from investigators.	6-7
Data items	11	List and define all variables for which data were sought (e.g., PICOS, funding sources) and any assumptions and simplifications made.	6-7; Table 1
Risk of bias in individual studies	12	Describe methods used for assessing risk of bias of individual studies (including specification of whether this was done at the study or outcome level), and how this information is to be used in any data synthesis.	7
Summary measures	13	State the principal summary measures (e.g., risk ratio, difference in means).	6-7 (narrative)
Synthesis of results	14	Describe the methods of handling data and combining results of studies, if done, including measures of consistency (e.g., I^2) for each meta-analysis.	N/A

For peer review only

For peer review only - http://bmjopen.bmj.com/site/about/guidelines.xhtml

Appendix S2: PRISMA 2009 Checklist

Section/topic	#	Checklist item	Reported on page #
Risk of bias across studies	15	Specify any assessment of risk of bias that may affect the cumulative evidence (e.g., publication bias, selective reporting within studies).	7
Additional analyses	16	Describe methods of additional analyses (e.g., sensitivity or subgroup analyses, meta-regression), if done, indicating which were pre-specified.	N/A
**RESULTS**			
Study selection	17	Give numbers of studies screened, assessed for eligibility, and included in the review, with reasons for exclusions at each stage, ideally with a flow diagram.	7; Figure 1
Study characteristics	18	For each study, present characteristics for which data were extracted (e.g., study size, PICOS, follow-up period) and provide the citations.	7; Appendix S4
Risk of bias within studies	19	Present data on risk of bias of each study and, if available, any outcome level assessment (see item 12).	7-8; Appendix S6
Results of individual studies	20	For all outcomes considered (benefits or harms), present, for each study: (a) simple summary data for each intervention group (b) effect estimates and confidence intervals, ideally with a forest plot.	8-13; Tables 2a+b; Appendix S5
Synthesis of results	21	Present results of each meta-analysis done, including confidence intervals and measures of consistency.	N/A
Risk of bias across studies	22	Present results of any assessment of risk of bias across studies (see Item 15).	7-8; Appendix S6
Additional analysis	23	Give results of additional analyses, if done (e.g., sensitivity or subgroup analyses, meta-regression [see Item 16]).	N/A
**DISCUSSION**			
Summary of evidence	24	Summarize the main findings including the strength of evidence for each main outcome; consider their relevance to key groups (e.g., healthcare providers, users, and policy makers).	13-16
Limitations	25	Discuss limitations at study and outcome level (e.g., risk of bias), and at review-level (e.g., incomplete retrieval of identified research, reporting bias).	14-15
Conclusions	26	Provide a general interpretation of the results in the context of other evidence, and implications for future research.	15-16
**FUNDING**			
Funding	27	Describe sources of funding for the systematic review and other support (e.g., supply of data); role of funders for the systematic review.	17

For peer review only - http://bmjopen.bmj.com/site/about/guidelines.xhtml

For peer review only

Appendix S2: PRISMA 2009 Checklist

From: Moher D, Liberati A, Tetzlaff J, Altman DG, The PRISMA Group (2009). Preferred Reporting Items for Systematic Reviews and Meta-Analyses: The PRISMA Statement. PLoS Med 6(7): e1000097. doi:10.1371/journal.pmed1000097

For more information, visit: www.prisma-statement.org.

For peer review only

For peer review only - http://bmjopen.bmj.com/site/about/guidelines.xhtml

Appendix S3: Primary reasons for article exclusion (n = 35)

	Population not postnatal	Intervention not targeted at management of BP	No control group	Study design
**n**	4	6	22	3
**Study IDs**	Berks 2015<br>Gerard 1983<br>Scardo 1999<br>Wacker 2006	Chandrasekaran 2015<br>Ehrenberg 2004<br>Ehrenberg 2006<br>Ossada 2016<br>Wasden 2012<br>Younger-Lewis 2016	Al Waili 2004<br>Alicino 1962<br>Barton 1991<br>Belfort 1988<br>Belfort 1992<br>Bittle 2014<br>Bosio 2003<br>Correa 1982<br>Dulitzky 1987<br>Hirshberg 2016<br>Hirshberg 2017<br>Hunter 1961<br>Onishi 2015<br>Robinson 1964<br>Rodriguez 2012<br>Saghir<br>Smith 2005<br>Sukerman-Voldman 1985<br>Taslimi 1991<br>Tkacheva 2006<br>Wacker 1994<br>Walters 1984	Editor, Emergency Medicine 1990<br>Cursino 2015<br>Gallegos 1961

For peer review only

APPENDIX S4: Main characteristics of included studies (n=39)

Study ID	Methods		Participants				Intervention		Outcomes	
	Study design	*Duration*	*n*[*]	*Age (yr)*[†]	*Setting*	*Country*	*Intervention*	*Control(s)*	*Primary*	*Secondary*
**ANTIHYPERTENSIVE MEDICATIONS (18 studies)**										
**Calcium channel blockers**										
**Barton 1990**[32]	RCT	Enrolled immediately after birth<br>Follow-up (F/U) 48h	31	24.0<br>26.3	Tertiary referral hospital	USA	Nifedipine 10mg oral (PO) 4-hourly for 48 hours	Placebo	Mean arterial pressure (MAP)	Systolic blood pressure (SBP)<br>Diastolic blood pressure (DBP)<br>Maternal heart rate<br>Antihypertensive medication requirement<br>Urine output<br>Laboratory values (urine protein, creatinine clearance, haematocrit (HCT), platelets (plt), alanine aminotransferase (ALT), blood urea nitrogen (BUN), creatinine, serum electrolytes, uric acid (UA), urine specific gravity)
Vermillion 1999[21]	RCT	Enrolled within 24h of birth<br>F/U 3 – 24h	21	27.2±7.3<br>27.0±6.4	Tertiary referral hospital	USA	Nifedipine 10mg stat PO then 20mg every 20 minutes until BP <160/110 or max 5 doses + intravenous (IV) placebo	Labetalol 20mg, then 40mg, then 80mg IV every 20 minutes until BP <160/110mmHg or max 5 doses (300mg) + oral placebo	SBP + DBP	SBP (failure to achieve target <160mmHg)<br>DBP (failure to achieve target <110mmHg)<br>Maternal side effects<br>Antihypertensive medication requirement<br>Urine output
**Sayin 2005**[34]	RCT	Enrolled 24h after birth<br>F/U 72h after BP controlled	83	17-41	Tertiary referral hospital	Turkey[‡]	Nifedipine 10mg PO QDS until BP <150/100mmHg for 48h	Methyldopa 250mg PO TDS	SBP + DBP	Maternal mortality<br>Antihypertensive medication requirement<br>Hypertensive retinopathy
**Vasodilators**										
Palot 1979[36]	Retrospective cohort study	Not specified	54	24.5 (17-37)	Not specified	France[†]	Hydralazine 5mg IV stat then 1% IV infusion, furosemide 20mg IV stat and 30% hypertonic glucose	(1) Clonidine IV and furosemide 20mg IV stat<br>OR<br>(2) Non-systematic treatment	Maternal morbidity (development of pre-eclampsia with severe features)	BP (time to resolution of hypertension)
**Griffis 1989**[38] [39]	RCT	F/U 24h	26	Not specified	Tertiary referral hospital	USA	Hydralazine 20mg IM QDS for 24h	Methyldopa 250mg IV QDS x 24h	MAP	Maternal side effects<br>Antihypertensive medication requirement<br>Urine output (time to diuresis)
**Walss Rodriguez 1991**[40]	RCT	Not specified	38	16-40	Not specified	Mexico[†]	Hydralazine 40mg PO QDS, duration not specified + if DBP > 110 PRN nifedipine 10mg SL every 30 minutes, to maximum of 3 doses	Nifedipine 10mg sub-lingual (SL) every 30 minutes if DBP >=110mmHg	SBP	DBP<br>Antihypertensive medication requirement
Begum 2002[17]	Quasi-randomised trial	Not specified	15	24.09±4.93<br>22.72±5.08	Tertiary referral hospital	Bangladesh	Hydralazine 5mg then 2mg IV bolus every 15 minutes until DBP 90-95mmHg	Hydralazine 20mg/200ml normal saline IV infusion; 10 drops per min, increased by 5 drops at 15 min intervals; until DBP 90-95mmHg	DBP	Maternal side effects<br>Antihypertensive medication requirement<br>Maternal heart rate
**Vigil-De Gracia 2007**[35]	RCT	Enrolled day 2-3 after birth<br>F/U not specified	82	29.9±5.9<br>31.3±5.5	Tertiary referral hospital	Panama	Hydralazine 5mg IV every 20 minutes until BP <160/110 or max 5 doses	Labetalol 20mg, then 40mg, then 80mg IV every 20 minutes until BP <160/110mmHg or max 5 doses (300mg)	SBP + DBP	Maternal mortality<br>Maternal morbidity (development of pre-eclampsia with severe features)<br>Maternal side effects<br>Antihypertensive medication requirement<br>Maternal heart rate
Hennessy 2007[23]	RCT	F/U 3h	37	21-43 (mean 33)	Tertiary referral hospital	Australia	Diazoxide 15mg IV every 3 minutes, maximum dose 300mg	Hydralazine 5mg IV every 20 minutes, maximum 15mg	SBP + DBP	SBP (10mmHg above target after 1 hour)<br>DBP (10mmHg above target after 1 hour)<br>Maternal side effects (including hypotension)<br>Time taken to administer drug
**Beta blockers**										
**Fidler 1982**[42]	RCT	Enrolled 4 days after birth<br>F/U 9 days	80	29.7±1.0<br>27.8±0.9	Tertiary referral hospital	UK	Timolol 5mg PO TDS for 9 days	Methyldopa 250mg PO TDS for 9 days	DBP	SBP<br>DBP (time to achieve control, proportion achieving control)<br>Maternal side effects
Garden 1982[24]	RCT	Enrolled immediately after birth<br>F/U 45-64h	6	25-44<br>20-28	Tertiary referral hospital	South Africa	Labetalol 200mg/200ml 5% dextrose, 20mg/h IV infusion, doubled every 30 minutes until DBP <100mmHg or maximum dose 160mg/h	Dihydralazine 100mg/200ml 5% dextrose, 10mg/h IV infusion, doubled every 30 minutes until DBP <100mmHg or maximum dose 80mg/h	DBP	Maternal side effects
Mabie 1987[22]	RCT	Enrolled 1-96 hours after birth<br>F/U 3h	41	23.7±6.9<br>22.9±7.0	Tertiary referral hospital	USA	Labetalol 20mg IV every 10 minutes then escalating until DBP <100mmHg or maximum cumulative dose reached (300mg)	Hydralazine 5mg IV every 10 minutes until DBP <100mmHg	MAP	MAP (time to maximal decrease)<br>DBP (achieving target <100mmHg)<br>Maternal side effects<br>Antihypertensive medication requirement<br>Maternal heart rate

[*] n = postnatal population (antenatal excluded)
[†] If given separately, intervention group followed by control group
[‡] Non-English language manuscript

For peer review only

Study ID	Methods		Participants				Intervention		Outcomes	
	Study design	*Duration*	*n**	*Age (yr)*†	*Setting*	*Country*	*Intervention*	*Control(s)*	*Primary*	*Secondary*
Shumard 2016[41]	Retrospective cohort study	F/U not specified (but >24h)	128	Not specified	Not specified	USA	Labetalol PO (variable dose and frequency)	Nifedipine PO (variable dose and frequency)	Length of hospital stay after birth	SBP<br>DBP<br>Antihypertensive medication requirement
Sharma 2017[27] [28]	RCT	4-6 weeks (BP outcomes)<br>6 months (duration antihypertensive medication)	50	34.0±7.4<br>33.3±6.4	Tertiary referral hospital	USA	Labetalol 200mg PO BD	Nifedipine XL 30mg PO OD	SBP + DBP	Maternal side-effects<br>Length of hospital stay after birth<br>Antihypertensive medication requirement
**Thiazides**										
Gaisin 2013[25]	RCT	6 months	30	23-29	Not specified (hospital)	Russia	Indapamide 1.5mg PO OD, duration unclear	Adjusted dose methyldopa	SBP + DBP	Safety data<br>Laboratory values (lipid and glucose metabolism)<br>Adherence to treatment<br>Weight reduction<br>Decrease in albuminuria<br>Decrease in LV mass index<br>Endothelial function<br>Milk production
Gaisin 2014[37]	RCT	1 year	30	24-28	Not specified (hospital)	Russia	Indapamide 1.5mg PO OD with ursodeoxycholic acid 250mg PO TDS, duration unclear	Adjusted dose methyldopa	SBP + DBP	Maternal side effects<br>Laboratory values (atherogenic lipid profile, glucose metabolism. renal function)<br>Offspring adverse events<br>Weight reduction<br>Decrease in microalbuminuria<br>Decrease in LV mass index<br>Endothelial function
**Indole alkaloids**										
Krebs A 1956[43] [44]	Retrospective cohort study	F/U not specified (but >24h)	140	Not specified	Not specified	Switzerland†	Reserpine 0.25mg PO or IM TDS or QDS for 7 days	Phenobarbital	SBP + DBP	SBP + DBP (non-responders)<br>Maternal side effects<br>Resolution of albuminuria<br>Resolution of oedema
**Centrally-acting alpha-agonists**										
Noronha Neto 2016[29-31]	RCT	Enrolled immediately after birth<br>F/U 4 days	90	28.9±6.7<br>28.8±6.7	Tertiary referral hospital	Brazil	Clonidine 0.1mg PO repeated every 20 minutes to maximum 6 doses	Captopril 25mg PO repeated every 20 minutes to maximum 6 doses	SBP + DBP	SBP (% reduction)<br>SBP + DBP (daily mean)<br>Maternal side effects<br>Antihypertensive medication requirement
**LOOP DIURETICS (4 studies)**										
**Matthews 1997[46]**	RCT	Enrolled 12-24h after birth<br>F/U 6 weeks	19	Not specified	Tertiary referral hospital	UK	Furosemide 40mg PO OD for 7 days	Placebo	MAP	Length of hospital stay after birth<br>Antihypertensive medication requirement<br>Urine output<br>Laboratory values (hypokalemia)
**Ascarelli 2005[16]**	RCT	Enrolled 2-24h after birth<br>F/U 6 weeks	264	22.8±6.1<br>22.9±6.0	Tertiary referral hospital	USA	Furosemide 20mg PO OD + potassium 20mEq PO OD for 5 days	No intervention	SBP	Maternal morbidity (postnatal complication requiring intervention)<br>DBP<br>Length of hospital stay after birth<br>Antihypertensive medication requirement<br>Maternal weight<br>Maternal HR<br>Duration of magnesium sulphate
Amorim 2015[45]	RCT	Enrolled immediately after birth<br>F/U 5 days	120	Not specified	Tertiary referral hospital	Brazil	Furosemide 40mg PO OD, duration not specified	Placebo	SBP + DBP	MAP<br>SBP (daily episodes >=180mmHg)<br>DBP (daily episodes >=110mmHg)<br>Length of hospital stay after birth<br>Antihypertensive medication requirement<br>Urine output<br>Maternal heart rate
Veena 2017[19]	RCT	Enrolled <24h after birth<br>F/U until hospital discharge	100	24.34±4.31<br>24.02±4.27	Tertiary referral hospital	India	Furosemide 20mg PO OD + nifedipine 10mg PO TDS for 3 days	Nifedipine 10mg PO TDS for 3 days	SBP + DBP	MAP<br>Maternal morbidity (postnatal complication requiring intervention)<br>Length of hospital stay after birth<br>Antihypertensive medication requirement<br>Urine output
**OTHER DRUGS (7 studies)**										
**Selective 5-HT antagonists**										
Weiner 1982[48]	RCT (crossover)	F/U not specified	5	Not specified	Tertiary referral	USA	R41468 IV (dose not specified) bolus then infusion for 90 minutes	Placebo	MAP	MAP (rate at which hypertension returned post infusion)<br>Urine output (infusion related diuresis)

For peer review only

Study ID	Methods		Participants				Intervention		Outcomes	
	Study design	*Duration*	*n*[*]	*Age (yr)*[†]	*Setting*	*Country*	*Intervention*	*Control(s)*	*Primary*	*Secondary*
Weiner 1984[49]	RCT<br>(crossover)	Enrolled immediately after birth<br>F/U 3.5h	20	28±6.4	Tertiary referral	USA	Ketanserin 10mg IV bolus then 4mg/hr IV infusion. Repeat bolus after 5 minutes if no response.	Placebo	SBP + DBP	Maternal side effects<br>DBP (achieving target <95mmHg)<br>MAP<br>Antihypertensive medication requirement<br>Response rate
Montenegro 1985[50]	RCT<br>(crossover)	Enrolled immediately after birth<br>F/U not specified	30	21.5 (13-31)	Tertiary referral hospital	USA	Ketanserin 10mg IV bolus, repeated if no response. If no response to second bolus IV infusion 4mg/hr (increments of 2mg/hr every 10 minutes to max 12mg/hr).	Placebo	MAP	Maternal side effects
**Alternative therapies**										
**Hladunewich 2006[51]**	RCT	Enrolled immediately after birth<br>F/U 10 days	45	29±6<br>28±7	Tertiary referral hospital	USA	L-arginine 3.5g PO QDS or 10g IV TDS for 3-9 days	Placebo	MAP	Maternal side effects<br>SBP<br>DBP<br>Antihypertensive medication requirement<br>Laboratory values (glomerlular filtration rate (GFR) (inulin clearance), Albumin/creatinine (A/C) ratio, vasoactive hormones (NO and cGMP), liver function tests (LFTs), plt)<br>Renal plasma flow (para-amino hippurate clearance), renal blood flow = renal plasma flow / (1-HCT), renovascular resistance = MAP / renal blood flow
Liu 2009[52]	Quasi-randomised trial	Enrolled day 2 after birth<br>F/U 3 weeks	72	26.6±3.7<br>25.7±3.9	District general hospital	China[†]	Shengkangbao 10g PO or IV BD for 3 weeks	No intervention	Percentage of cases with positive albuminuria	SBP<br>DBP<br>Laboratory values (24h urinary albumin, plasma total protein, plasma albumin, urinary albumin negative inversion rate, renal function)
**Steroids**										
Barrilleaux 2005[53 54]	RCT	Enrolled immediately after birth<br>F/U 4.5 days	157<br>(175)	24.5±6.8<br>23.9±6.4	Tertiary referral hospital	USA	Dexamethasone 10mg x 2, then 5mg x 2 IV BD for 48 hours	Placebo	Antihypertensive medication requirement	MAP<br>Critical care admission<br>Length of hospital stay after birth<br>Urine output<br>Laboratory values (plt, lactate dehydrogenase (LDH), aspartate aminotransferase (AST))<br>Stay in recovery >24h
**Atrial natriuretic peptide**										
Shigemitsu 2015[47]	Retrospective cohort study	F/U not specified	16	Not specified	Tertiary referral hospital	Japan	Carperitide (no further details)	Standard care	MAP	Maternal mortality<br>Maternal side effects<br>Need for dialysis<br>Time to diuresis
**UTERINE CURETTAGE (8 studies)**										
Salvatore 1967[58]	Prospective cohort study	Enrolled immediately after birth<br>F/U 10 days	48	16-45	Tertiary referral hospital	Brazil[††]	Uterine curettage	No intervention	SBP + DBP	Maternal morbidity (development of pre-eclampsia with severe features – seizures)
Magann 1993[59]	RCT	Enrolled immediately after birth<br>F/U 24h (telephone at 7 weeks)	32	22.9±5.6<br>23.4±6.6	Tertiary referral hospital	USA	Uterine curettage	No intervention	MAP	Maternal side effects<br>Length of hospital stay after birth<br>Antihypertensive medication requirement<br>Urine output<br>Laboratory values (HCT, plt, AST, LDH)
Magann 1994[60]	RCT	Enrolled immediately after birth<br>F/U 48h (telephone at 7 weeks)	45	22.3±6.4<br>22.8±6.6<br>22.8±6.1	Tertiary referral hospital	USA	Uterine curettage	(1) Nifedipine PO<br>OR<br>(2) Usual care	MAP	Maternal side effects<br>Urine output<br>Laboratory values (HCT, plt, AST, LDH)
Gocmen 1996[57]	Prospective cohort study	Enrolled immediately after birth<br>F/U 24h	50	Not specified	Tertiary referral hospital	Turkey[†]	Uterine curettage	No intervention	MAP	Urine output<br>Laboratory values (plt)
Gomez 2005[61]	RCT	Enrolled immediately after birth<br>F/U not specified	86	Not specified	Tertiary referral hospital	Peru	Uterine curettage	No intervention	MAP	Maternal side effects<br>Length of hospital stay after birth<br>Antihypertensive medication requirement<br>Urine output

For peer review only

Study ID	Methods		Participants				Intervention		Outcomes	
	Study design	*Duration*	*n*[*]	*Age (yr)*[†]	*Setting*	*Country*	*Intervention*	*Control(s)*	*Primary*	*Secondary*
Alkan 2006[62]	RCT	Enrolled immediately after birth<br>F/U 24h	56	22.8±3.4<br>24.6±7.5	Tertiary referral hospital	Turkey	Uterine curettage	No intervention	MAP	Maternal side effects<br>Urine output<br>Laboratory values (plt, LDH, AST, ALT)
Ragab 2013[15]	RCT	Enrolled immediately after birth<br>F/U 96h	420	Not specified	Tertiary referral hospital	Egypt	Uterine curettage	No intervention	MAP	Maternal mortality<br>Maternal morbidity (development of pre-eclampsia with severe features)<br>MAP (time to MAP <=105mmHg)<br>Urine output<br>Laboratory values (creatinine, plt, UA)
Mallapur 2015[18]	RCT	Enrolled immediately after birth<br>F/U 7 days	100	Not specified	Tertiary referral hospital	India	Uterine curettage	No intervention	MAP	Length of hospital stay after birth<br>Urine output<br>Laboratory values (plt, renal and liver function)
**ORGANISATION OF CARE (2 studies)**										
York 1997[26]	RCT	Enrolled immediately after birth<br>F/U 8 weeks	96[§]	28±7<br>27±7	Tertiary referral	USA	Contact with nurse specialist; early discharge if criteria met; 2 scheduled home visits and 10 telephone calls (twice weekly for 2 weeks, then weekly for 6 weeks) during 8-week F/U	Standard care	Postnatal readmission to secondary care	Functional status<br>Patient satisfaction with care<br>Neonatal rehospitalisation / acute neonatal care<br>Cost
Bibbo 2014[33]	Retrospective cohort study	F/U not specified (but >7 days)	138	Not specified	Tertiary referral hospital	USA	Specialised postpartum clinic	Usual care	Postnatal readmission to secondary care and triage visits	Primary care provider F/U

[§] Mixture of hypertension and diabetes – unable to separate

For peer review only

APPENDIX S5: Summary of main results for included studies (n=39)

ANTIHYPERTENSIVE MEDICATIONS (18 studies)

Calcium channel blockers

Study ID: Barton 1990[32]

Population: Postnatal women with severe pre-eclampsia
Setting: Tertiary referral centres, USA
Intervention: Nifedipine 10mg PO 4-hourly for 48 hours
Comparison: Placebo

Primary outcome	Treatment effect	Number of participants	Quality of the evidence	Comments
MAP (18-24 hours after birth)	Intervention group 93.9±1.6mmHg, control group 100.2±2.6mmHg. Difference 6.3mmHg (p<0.05).	31 (16 intervention, 15 control). Follow-up complete for all participants.	Double-blind RCT. Overall low risk of bias.	

Study ID: Vermillion 1999[21]

Population: Antenatal and postnatal women with severe pre-eclampsia or super-imposed pre-eclampsia
Setting: Tertiary referral centres (USA)
Intervention: Nifedipine 10mg stat PO then 20mg every 20 minutes until BP <160/110mmHg or max 5 doses (90mg) + IV placebo
Comparison: Labetalol 20mg, then 40mg, then 80mg IV every 20 minutes until BP <160/110mmHg or max 5 doses (300mg) + PO placebo

Primary outcome	Treatment effect	Number of participants	Quality of the evidence	Comments
SBP + DBP (time to target <160/100mmHg)	Intervention group 25.1±13.6 minutes, control group 43.6±25.4 minutes. Difference 18.5 minutes (p=0.002).	50 (21 postnatal – 10 intervention, 11 control). Follow-up complete for all participants.	Double-blind RCT. Overall high risk of bias (other bias).	Small number of postnatal women (42%) (n<30). Unable to obtain data for postnatal subgroup.

Study ID: Sayin 2005[34]

Population: Postnatal women with pre-eclampsia, severe pre-eclampsia, superimposed pre-eclampsia or eclampsia
Setting: Tertiary referral centres (Turkey)
Intervention: Nifedipine 10mg PO 6-hourly until BP <150/100mmHg for 48 hours
Comparison: Methyldopa 250mg PO 8-hourly

Primary outcome	Treatment effect	Number of participants	Quality of the evidence	Comments
SBP + DBP (time to target <150/100mmHg)	Intervention group 6.7±2.5 days; control group 8.6±5.5 days. Difference 1.9 days (NS).	83 (42 intervention, 41 control). Follow-up complete for all participants.	Open-label RCT. Overall high risk of bias (multiple domains).	

Vasodilators

Study ID: Palot 1979[36]

Population: Postnatal women with 'arterial hypertensions of labour and the postpartum period'
Setting: Not specified (France)
Intervention: Hydralazine 5mg IV stat then 1% IV infusion, furosemide 20mg IV stat and 30% hypertonic glucose
Comparison: Clonidine IV and furosemide 20mg IV stat

Primary outcome	Treatment effect	Number of participants	Quality of the evidence	Comments
Maternal morbidity (development of pre-eclampsia with severe features)	Intervention group: no women developed eclampsia. Control group: 2 women developed eclampsia, No statistical analysis.	54 (11 intervention, 24 control, 19 non-systematic treatment). Completeness of follow-up not specified.	Retrospective cohort study. Overall high risk of bias (comparability).	No statistical analysis.

Study ID: Griffis 1989[38 39]

Population: Postnatal women with pre-eclampsia
Setting: Tertiary referral centres (USA)
Intervention: Hydralazine 20mg IM 6-hourly for 24h
Comparison: Methyldopa 250mg IV 6-hourly for 24h

Primary outcome	Treatment effect	Number of participants	Quality of the evidence	Comments
MAP (mean at 6 and 12 hours)	6 hours: intervention group 104.5mmHg, control group 112mmHg. Difference 7.5mmHg (p=0.0057).<br>12 hours: intervention group 100mmHg, control group108mmHg. Difference 8mmHg (NS).	26 (12 intervention, 14 control). Follow-up complete for all participants.	Open-label RCT. Overall high risk of bias (multiple domains).	Small sample size (n<30).

Study ID: Walss Rodriguez 1991[40]

Population: Postnatal women with severe pre-eclampsia
Setting: Not specified (Mexico)
Intervention: Hydralazine 40mg PO 6-hourly, duration not specified + if DBP > 110mmHg PRN nifedipine 10mg sublingual every 30 minutes, to maximum of 3 doses (30mg)
Comparison: Nifedipine 10mg sublingual every 30 minutes if DBP ≥ 110mmHg

Primary outcome	Treatment effect	Number of participants	Quality of the evidence	Comments
SBP (mean)	Intervention group 143.6mmHg, control group138.0mmHg. Difference 5.6mmHg (NS).	38 (18 intervention, 20 control). Completeness of follow-up not specified.	Open-label RCT. Overall high risk of bias (multiple domains).	

Study ID: Begum 2002[17]

Population: Antenatal and postnatal women with eclampsia
Setting: Tertiary referral centres (Bangladesh)
Intervention: Hydralazine 5mg then 2mg IV bolus every 15 minutes until DBP 90-95mmHg
Comparison: Hydralazine 20mg /200ml normal saline IV infusion; 10 drops per min, increased by 5 drops at 15 min intervals; until DBP 90-95mmHg

For peer review only

Primary outcome	Treatment effect	Number of participants	Quality of the evidence	Comments
DBP (time to target 90-95mmHg)	Intervention group 65.23±23.38 minutes, control group 186.36±79.77 minutes. Difference 121.13 minutes (p<0.001).	77 (15 postnatal – 9 intervention, 6 control). Completeness of follow-up not specified.	Open-label quasi-randomised trial. Overall high risk of bias (multiple domains).	Small number of postnatal women (19%) (n<30). Unable to obtain data for postnatal subgroup.

Study ID: Vigil de Gracia 2007[35]

Population: Postnatal women with severe gestational hypertension, severe pre-eclampsia or super-imposed pre-eclampsia
Setting: Tertiary referral centres (Panama)
Intervention: Hydralazine 5mg IV every 20 minutes until BP <160/110mmHg or maximum 5 doses
Comparison: Labetalol 20mg, then 40mg, then 80mg IV every 20 minutes until BP <160/110mmHg or maximum 5 doses (300mg)

Primary outcome	Treatment effect	Number of participants	Quality of the evidence	Comments
SBP + DBP (persistent hypertension >=160/110mmHg after 5 doses of medication)	Intervention group 0/42, control group 1/40 (NS).	82 (42 intervention, 40 control). Follow-up complete for all participants.	Open-label RCT. Overall high risk of bias (multiple domains).	

Study ID: Hennessy 2007[23]

Population: Antenatal and postnatal women with pre-eclampsia, superimposed pre-eclampsia or essential hypertension
Setting: Tertiary referral (Australia)
Intervention: Diazoxide 15mg IV every 3 minutes, until target BP (140/90mmHg) reached or maximum cumulative dose 300mg
Comparison: Hydralazine 5mg IV every 20 minutes, until target BP (140/90mmHg) reached or maximum cumulative dose 15mg

Primary outcome	Treatment effect	Number of participants	Quality of the evidence	Comments
SBP + DBP (proportion achieving target BP <=140/90mmHg)	Intervention group 67%, control group 43% (p<0.01). RR 0.637 (95% CI 0.456-0.89) for not reaching target BP with intervention.	124 total (37 postnatal – 11 intervention, 16 control). Follow-up complete for all participants.	Open-label RCT. Overall high risk of bias (multiple domains).	Small proportion of postnatal women (30%). Unable to obtain data for postnatal subgroup.

Beta-blockers

Study ID: Garden 1982[24]

Population: Antenatal and postnatal women with severe pre-eclampsia or eclampsia
Setting: Tertiary referral (South Africa)
Intervention: Labetalol 200mg/200ml 5% dextrose, 20mg/h IV infusion, doubled every 30 minutes until DBP <100mmHg or maximum dose 160mg/hour
Comparison: Dihydralazine 100mg/200ml 5% dextrose, 10mg/h IV infusion, doubled every 30 minutes until DBP <100mmHg or maximum dose 80mg/hour

Primary outcome	Treatment effect	Number of participants	Quality of the evidence	Comments
DBP (proportion achieving target DBP 90-100mHg within 2 hours)	Intervention group 5/6, control group 2/6. No statistical analysis.	12 total (6 postnatal – 3 intervention, 3 control). Follow-up complete for all participants.	RCT (blinding not specified). Overall high risk of bias (other bias).	Very small sample size (n<15). Unable to obtain data for postnatal subgroup.

Study ID: Fidler 1982[42]

Population: Postnatal women with gestational hypertension
Setting: Tertiary referral (UK)
Intervention: Timolol 5mg PO 8-hourly for 9 days
Comparison: Methyldopa 250mg PO 8-hourly for 9 days

Primary outcome	Treatment effect	Number of participants	Quality of the evidence	Comments
DBP (day 1)	Intervention group 88.7mmHg, control group 93.8mmHg. Difference 5.1mmHg (p<0.05).	80 (40 intervention, 40 control). Follow-up complete in 79/80	RCT (blinding not specified). Overall high risk of bias (multiple domains).	

Study ID: Mabie 1987[22]

Population: Antenatal and postnatal women with pre-eclampsia, superimposed pre-eclampsia, eclampsia or essential hypertension
Setting: Tertiary referral (USA)
Intervention: Labetalol 20mg IV every 10 minutes then escalating until DBP < 100mmHg or maximum cumulative dose reached (300mg)
Comparison: Hydralazine 5mg IV every 10 minutes until DBP < 100mmHg

Primary outcome	Treatment effect	Number of participants	Quality of the evidence	Comments
MAP (mean maximal decrease)	Intervention group 25.5±11.2mmHg, 33.3±13.2mmHg control group. Difference 7.8mmHg (p=0.02).	60 (41 postnatal – 27 intervention, 14 control). Follow-up complete for all participants.	Open-label RCT. Overall high risk of bias (multiple domains).	

Study ID: Shumard 2016[41]

Population: Postnatal women with gestational hypertension or pre-eclampsia
Setting: Not specified (USA)
Intervention: Labetalol PO (variable dose and frequency)
Comparison: Nifedipine PO (variable dose and frequency)

Primary outcome	Treatment effect	Number of participants	Quality of the evidence	Comments
Length of hospital stay after delivery	Intervention group 3.5 days, control group 3.6 days. Difference 0.1 days (NS).	128 (42 intervention, 86 control). Follow-up complete for all participants.	Retrospective cohort study. Overall high risk of bias (comparability).	Conference abstract only. Authors did not provide further data.

Study ID: Sharma 2017[27 28]

Population: Postnatal women with gestational hypertension or pre-eclampsia
Setting: Tertiary referral (USA)
Intervention: Labetalol 200mg PO 12-hourly, increased to 800mg PO 12-hourly as needed
Comparison: Nifedipine XL 30mg PO once daily, increased to 90mg PO once daily as needed

Primary outcome	Treatment effect	Number of participants	Quality of the evidence	Comments

For peer review only

Primary outcome	Treatment effect	Number of participants	Quality of the evidence	Comments
SBP + DBP (time to sustained BP control: absence of severe hypertension for >=12 hours)	Intervention group 37.6 hours, control group 38.2 hours. Difference 0.6 hours (NS).	50 (25 intervention, 25 control).<br>Follow-up complete for all participants.	Open-label RCT.<br>Overall high risk of bias (multiple domains).	
**Thiazides**				
Study ID: Gaisin 2013[25]				
**Population:** Postnatal women with pre-eclampsia, super-imposed pre-eclampsia or essential hypertension<br>**Setting:** Not specified (Russia)<br>**Intervention:** Indapamide 1.5mg PO OD, duration unclear<br>**Comparison:** Adjusted dose methyldopa				
**Primary outcome**	**Treatment effect**	**Number of participants**	**Quality of the evidence**	**Comments**
Systolic and diastolic BP	Intervention group 113±6/74±4mmHg, control group 116±5/75±4mmHg (NS).	30 (15 intervention, 15 control).<br>Completeness of follow-up not specified.	Open-label RCT.<br>Overall high risk of bias (multiple domains).	Conference abstract only. Authors did not provide further data.
Study ID: Gaisin 2014[37]				
**Population:** Postnatal women with pre-eclampsia<br>**Setting:** Not specified (Russia)<br>**Intervention:** Indapamide 1.5mg PO once daily + ursodeoxycholic acid 250mg PO three times daily, duration unclear<br>**Comparison:** Adjusted dose methyldopa				
**Primary outcome**	**Treatment effect**	**Number of participants**	**Quality of the evidence**	**Comments**
SBP + DBP	Intervention group 122±6/75±4 mmHg, control group 126±6/78±5mmHg (NS).	30 (allocation not described).<br>Completeness of follow-up not specified.	Open-label RCT.<br>Overall high risk of bias (multiple domains).	Conference abstract only. Authors did not provide further data. Number of participants in each group not stated.
**Indole alkaloids**				
Study ID: Krebs 1956[43 44]				
**Population:** Postnatal women with gestational hypertension, pre-eclampsia, severe pre-eclampsia or eclampsia<br>**Setting:** Not specified (Germany)<br>**Intervention:** Reserpine 0.25mg PO or intramuscular 6-8 hourly for 7 days<br>**Comparison:** Phenobarbital				
**Primary outcome**	**Treatment effect**	**Number of participants**	**Quality of the evidence**	**Comments**
SBP + DBP (maximal reduction)	Intervention halved time to maximal BP reduction (no further details reported).<br>No statistical analysis.	140 (70 intervention, 70 control).<br>Completeness of follow-up not specified.	Retrospective cohort study.<br>Overall high risk of bias (selection and outcome assessment).	No statistical analysis.
**Centrally-acting alpha agonists**				
Study ID: Noronha Neto 2016[29-31]				
**Population:** Postnatal women with severe hypertensive disorders of pregnancy<br>**Setting:** Tertiary referral (Brazil)<br>**Intervention:** Clonidine 0.1mg PO repeated every 20 minutes to max 6 doses<br>**Comparison:** Captopril 25mg PO repeated every 20 minutes to max 6 doses				
**Primary outcome**	**Treatment effect**	**Number of participants**	**Quality of the evidence**	**Comments**
SBP + DBP (episodes SBP >=180mmHg and/or DBP >=110mmHg)	Intervention group 2.1±2.1 episodes, control group 3.5±4.7 episodes (NS).	90 (45 intervention, 45 control).<br>Follow-up complete in 88/90.	Double-blind RCT.<br>Overall low risk of bias.	
**DIURETICS (4 studies)**				
Study ID: Matthews 1997[46]				
**Population:** Postnatal women with severe pre-eclampsia or eclampsia<br>**Setting:** Tertiary referral centres (UK)<br>**Intervention:** Furosemide 40mg PO once daily for 7 days<br>**Comparison:** Placebo				
**Primary outcome**	**Treatment effect**	**Number of participants**	**Quality of the evidence**	**Comments**
MAP (decrease)	Intervention group -10.6mmHg, control group -9.75mmHg (NS).	19 (10 intervention, 9 control).<br>Follow-up complete in 18/19.	Double-blind RCT.<br>Overall high risk of bias (other bias).	Small sample size (n<30).
Study ID: Ascarelli 2005[16]				
**Population:** Postnatal women with pre-eclampsia, severe pre-eclampsia or superimposed pre-eclampsia<br>**Setting:** Tertiary referral centres (USA)<br>**Intervention:** Furosemide 20mg PO once daily + potassium 20mEq PO once daily for 5 days<br>**Comparison:** No intervention				
**Primary outcome**	**Treatment effect**	**Number of participants**	**Quality of the evidence**	**Comments**
SBP	No significant difference between groups (details not reported).<br>Severe pre-eclampsia (n=70) day 2 SBP intervention group 142±13mmHg, control group 153±19mmHg. Difference 11mmHg (p<0.004).	264 (132 intervention, 132 control).<br>Completeness of follow-up not specified.	Open-label RCT.<br>Overall high risk of bias (multiple domains).	
Study ID: Amorim 2015[45]				

For peer review only

Population: Postnatal women with severe pre-eclampsia
Setting: Tertiary referral (Brazil)
Intervention: Furosemide 40mg PO once daily for maximum 5 days
Comparison: Placebo

Primary outcome	Treatment effect	Number of participants	Quality of the evidence	Comments
SBP + DBP	Intervention group had significantly improved SBP + DBP. Magnitude of difference not reported (p<0.001).	120 (allocation not described). Follow-up complete in 118/120.	Double-blind RCT. Overall high risk of bias (reporting bias).	Conference abstract only. Authors did not provide further data. Number of participants in each group not stated.

Study ID: Veena 2017[19]

Population: Postnatal women with severe pre-eclampsia
Setting: Tertiary referral centre (India)
Intervention: Furosemide 10mg PO once daily plus nifedipine 10mg PO three times daily for 3 days
Comparison: Nifedipine 10mg PO three times daily for 3 days

Primary outcome	Treatment effect	Number of participants	Quality of the evidence	Comments
SBP + DBP	No significant difference between groups (absolute values and differences not reported, p=0.457 for SBP and p=0.642 for DBP).	100 (50 intervention, 50 control). Follow-up complete in 98/100 (49 intervention, 49 control).	Open-label RCT. Overall high risk of bias (multiple domains).	

OTHER DRUGS (7 studies)

Selective 5-HT antagonists

Study ID: Weiner 1982[48]

Population: Postnatal women with severe pre-eclampsia
Setting: Tertiary referral (USA)
Intervention: R41468 IV (dose not specified) bolus then infusion for 90 minutes
Comparison: Placebo

Primary outcome	Treatment effect	Number of participants	Quality of the evidence	Comments
MAP (mean maximal decline)	Intervention group 31.6mmHg, control group 6.0mmHg. Difference 25.6mmHg (p<0.001).	5 (crossover). Follow-up complete in all participants.	Double blind RCT (crossover). Overall high risk of bias (other bias).	Conference abstract only. Authors did not provide further data. Very small sample size (n<15).

Study ID: Weiner 1984[49]

Population: Postnatal women with pre-eclampsia and super-imposed pre-eclampsia
Setting: Tertiary referral (USA)
Intervention: Ketanserin 10mg IV bolus then 4mg/hr IV infusion. Repeat bolus after 5 minutes if no response.
Comparison: Placebo

Primary outcome	Treatment effect	Number of participants	Quality of the evidence	Comments
SBP + DBP (mean maximal decline)	Intervention group 41/34mmHg, control group 7/7mmHg. Difference 34/27mmHg (p<0.001).	20 (crossover). Follow-up complete in all participants.	Double blind RCT (crossover). Overall high risk of bias (other bias).	Small sample size (n<30).

Study ID: Montenegro 1985[50]

Population: Postnatal women with pre-eclampsia
Setting: Tertiary referral (USA)
Intervention: Ketanserin 10mg IV bolus, repeated if no response. If no response to second bolus IV infusion 4mg/hr (increments of 2mg/hr every 10 minutes to max 12mg/hr).
Comparison: Placebo

Primary outcome	Treatment effect	Number of participants	Quality of the evidence	Comments
MAP	Intervention group had significantly improved MAP, over 30 minutes after drug administered. F = 9.66 (p <0.01)	30 (crossover). Follow-up complete in 23/30.	Double blind RCT (crossover). Overall high risk of bias (multiple domains).	

Alternative therapies

Study ID: Hladunewich 2006[51]

Population: Postnatal women with pre-eclampsia
Setting: Tertiary referral (USA)
Intervention: L-arginine 3.5g PO four times daily OR L-arginine 10g IV three times daily (if unable to take PO) for 3-9 days postpartum
Comparison: Placebo

Primary outcome	Treatment effect	Number of participants	Quality of the evidence	Comments
MAP	Intervention group day 3 102±12 mmHg and day 10 98±14 mmHg; control group day 3 103±12mmHg and day 10 96±11 mmHg. Difference day 3 1mmHg, day 10 2mmHg (NS).	45 (22 intervention, 23 control). Follow-up complete in 39/45.	Double blind RCT. Overall high risk of bias (multiple domains).	

Study ID: Liu 2009[52]

Population: Postnatal women with severe pre-eclampsia
Setting: District general (China)
Intervention: Shengkangbao 10g PO or IV twice daily for 3 weeks
Comparison: No intervention

Primary outcome	Treatment effect	Number of participants	Quality of the evidence	Comments

For peer review only

Percentage of cases with positive albuminuria	At 3 weeks intervention group 0.7+/-0.8% positive albuminuria, control group 1.5+/-0.9%. Difference 0.8% (p<0.01).	77 (allocation not described). Follow-up complete in 72 (38 intervention, 32 control)	Open-label quasi-randomised study. Overall high risk of bias (multiple domains).	Clinical significance of primary outcome unclear.
**Steroids**				
Study ID: Barrilleaux 2005[53 54]				
**Population:** Postnatal women with severe pre-eclampsia and eclampsia<br>**Setting:** Tertiary referral (USA)<br>**Intervention:** Dexamethasone 10mg x2, then 5mg x 2 IV 12-hourly for 48 hours<br>**Comparison:** Placebo (IV saline)				
**Primary outcome**	**Treatment effect**	**Number of participants**	**Quality of the evidence**	**Comments**
Anti-hypertensive medication requirement	Intervention group 38/77, control group 31/80 required antihypertensive treatment in the first 48h PN (NS).	157 (77 intervention, 80 control). Follow-up complete in 155/157.	Double blind RCT. Overall high risk of bias (reporting bias).	
**Atrial natriuretic peptide**				
Study ID: Shigemitsu 2015[47]				
**Population:** Postnatal women with severe pre-eclampsia, HELLP syndrome or placental abruption<br>**Setting:** Tertiary referral (Japan)<br>**Intervention:** Carperitide (no further details supplied)<br>**Comparison:** No intervention				
**Primary outcome**	**Treatment effect**	**Number of participants**	**Quality of the evidence**	**Comments**
MAP	Intervention group had significantly improved MAP at 48 hours. Magnitude of difference not reported, no p value presented.	16 (6 intervention, 10 control) Follow-up complete for all participants.	Retrospective cohort study. Overall high risk of bias (comparability).	Conference abstract only. Authors did not provide further data. Small sample size (n<30)
**UTERINE CURETTAGE (8 studies)**				
Study ID: Salvatore 1967[58]				
**Population:** Postnatal women with severe pre-eclampsia or eclampsia<br>**Setting:** Tertiary referral (Brazil)<br>**Intervention:** Uterine curettage<br>**Comparison:** No intervention				
**Primary outcome**	**Treatment effect**	**Number of participants**	**Quality of the evidence**	**Comments**
SBP + DBP (proportion achieving target <140/90mmHg)	24 hours: intervention group 45%, control group 11%. No statistical analysis. 48 hour: intervention group 70%, control group 29%. No statistical analysis.	48 (20 intervention, 28 control) Follow-up complete for all participants.	Prospective cohort study. Overall high risk of bias (comparability).	Significant differences in study group populations (9/20 intervention group eclamptic at enrolment, 28/28 control group).
Study ID: Magann 1993[59]				
**Population:** Postnatal women with severe pre-eclampsia<br>**Setting:** Tertiary referral (USA)<br>**Intervention:** Uterine curettage<br>**Comparison:** No intervention				
**Primary outcome**	**Treatment effect**	**Number of participants**	**Quality of the evidence**	**Comments**
MAP	Intervention group had significantly improved MAP to 24 hours after birth. Difference 6-10mmHg (most significant at 16 hours p<0.0002).	32 (16 intervention, 16 control). Completeness of follow-up not specified.	Open-label RCT. Overall high risk of bias (multiple domains).	
Study ID: Magann 1994[60]				
**Population:** Postnatal women with severe pre-eclampsia<br>**Setting:** Tertiary referral (USA)<br>**Intervention:** Uterine curettage<br>**Comparison:** Oral nifedipine OR no intervention				
**Primary outcome**	**Treatment effect**	**Number of participants**	**Quality of the evidence**	**Comments**
MAP	Intervention group had significantly improved MAP 8-48 hours after birth. Difference 9-13mmHg (p=0.0017). No difference between curettage and nifedipine.	45 (15 intervention, 15 each control group) Completeness of follow-up not specified.	Open-label RCT. Overall high risk of bias (multiple domains).	
Study ID: Gocmen 1996[57]				
**Population:** Postnatal women with pre-eclampsia<br>**Setting:** Tertiary referral (Turkey)<br>**Intervention:** Uterine curettage<br>**Comparison:** No intervention				
**Primary outcome**	**Treatment effect**	**Number of participants**	**Quality of the evidence**	**Comments**
MAP	Intervention group had significantly improved MAP to 24 hours after birth. Magnitude of difference not reported (p=0.01).	50 (30 intervention, 20 control) Completeness of follow-up not specified.	Prospective cohort study. Overall high risk of bias (comparability and outcome assessment).	Conference abstract only. Authors did not provide further data.
Study ID: Gomez 2005[61]				

Population: Postnatal women with severe pre-eclampsia
Setting: Tertiary referral (Peru)
Intervention: Uterine curettage
Comparison: No intervention

Primary outcome	Treatment effect	Number of participants	Quality of the evidence	Comments
MAP	Intervention group had significantly improved MAP. Time point not specified. Magnitude of difference not reported (p<0.001).	86 (27 intervention, 59 control) Completeness of follow-up not specified.	Open-label RCT. Overall high risk of bias (multiple domains).	Conference abstract only. Authors did not provide further data.

Study ID: Alkan 2006[62]

Population: Postnatal women with severe pre-eclampsia
Setting: Tertiary referral (Turkey)
Intervention: Uterine curettage
Comparison: No intervention

Primary outcome	Treatment effect	Number of participants	Quality of the evidence	Comments
MAP	24 hours: Intervention group 103.4±7.8 mmHg, control group 110.2±4.8. Difference 6.8mmHg (p<0.05).	56 (31 intervention, 25 control) Follow-up complete for all participants.	Open-label RCT. Overall high risk of bias (multiple domains).	

Study ID: Ragab 2013[15]

Population: Postnatal women with severe pre-eclampsia or eclampsia
Setting: Tertiary referral (Egypt)
Intervention: Uterine curettage
Comparison: No intervention

Primary outcome	Treatment effect	Number of participants	Quality of the evidence	Comments
MAP	6 hours: Intervention group 140.1±6.12 mmHg, control group 152.4±3.7 mmHg. Difference 12.3mmHg (p=0.02).<br>24 hours: Intervention group 101.4±7.14 mmHg, control group 110.6±2.22 mmHg. Difference 9.2mmHg (p=0.01).	420 (220 intervention, 200 control) Follow-up complete for all participants.	Open-label RCT. Overall high risk of bias (multiple domains).	

Study ID: Mallapur 2015[18]

Population: Postnatal women with severe pre-eclampsia or eclampsia
Setting: Tertiary referral (India)
Intervention: Uterine curettage
Comparison: No intervention

Primary outcome	Treatment effect	Number of participants	Quality of the evidence	Comments
MAP	From 4 hours after birth: Intervention group 116±4.4 mmHg, control group 123.6±6.1 mmHg. Difference 7.6mmHg (p<0.001).	100 (50 intervention, 50 control) Completeness of follow-up not specified.	Open-label RCT. Overall high risk of bias (multiple domains).	Conference abstract only. Authors did not provide further data.

ORGANISATION OF CARE (2 studies)

Study ID: York 1997[26]

Population: Postnatal women with pre-eclampsia or essential hypertension, or diabetes
Setting: Tertiary referral (USA)
Intervention: Nurse specialist follow-up
Comparison: No intervention

Primary outcome	Treatment effect	Number of participants	Quality of the evidence	Comments
Postnatal readmission to secondary care	No significant difference between groups.	96 (44 intervention, 52 control) Completeness of follow-up not specified.	Open-label RCT. Overall high risk of bias (multiple domains).	Population mixed diabetes and/or hypertension – unable to separate.

Study ID: Bibbo 2014[33]

Population: Postnatal women with pre-eclampsia
Setting: Tertiary referral (USA)
Intervention: Specialised postpartum clinic
Comparison: No intervention

Primary outcome	Treatment effect	Number of participants	Quality of the evidence	Comments
Postnatal readmission to secondary care and triage visits	Intervention group 21.7%; control group 8.7% (p<0.039)	138 (69 intervention, 69 control) Completeness of follow-up not specified.	Retrospective cohort study. Overall high risk of bias (comparability).	Conference abstract only. Authors did not provide further data.

For peer review only

Appendix S6: Risk of bias in included studies (n=38)

Appendix S6a: Risk of bias in included RCTs and quasi-randomised studies (n=31)

Study ID	Random sequence generation	Allocation concealment	Blinding of participants and personnel	Blinding of outcome assessment	Incomplete outcome data	Selective reporting	Other bias
**ANTIHYPERTENSIVE MEDICATIONS**							
Fidler 1982[42]	Unclear	Unclear	Unclear	Unclear	High	High	Low
Garden 1982[24]	Unclear	Unclear	Unclear	Unclear	Low	Unclear	High
Mabie 1987[22]	Unclear	Unclear	High	High	Low	Low	High
Griffis 1989[38 39]	Unclear	Low	High	High	High	High	High
Barton 1990[32]	Low	Low	Low	Low	Low	Low	Low
Walss Rodriguez 1991[40]	Low	Low	High	High	Unclear	Unclear	Low
Vermillion 1999[21]	Low	Low	Low	Low	Low	Low	High
Begum 2002[17]	High	High	High	High	Unclear	Unclear	High
Sayin 2005[34]	Unclear	Unclear	High	High	Low	Unclear	High
Hennessy 2007[23]	Unclear	Low	High	High	Low	Low	High
Vigil-de-Gracia 2007[35]	Low	Low	High	High	Low	Low	Low
Gaisin 2013[25]	Unclear	Unclear	High	High	Unclear	High	High
Gaisin 2014[37]	Unclear	Unclear	High	High	Unclear	Unclear	High
Noronha Neto 2016[29-31]	Low	Low	Low	Unclear	Low	Low	Low
Sharma 2017[27 28]	Low	Low	High	High	Unclear	Low	Low
**DIURETICS**							
Matthews 1997[46]	Unclear	Low	Low	Low	Low	Unclear	High
Ascarelli 2005[16]	Unclear	Low	High	High	Unclear	High	Low
Amorim 2015[45]	Low	Low	Low	Low	Low	High	Low
Veena 2017[19]	Low	Low	High	High	Unclear	Unclear	Unclear
**OTHER DRUGS**							
Weiner 1982[48]	Unclear	Unclear	Low	Low	Unclear	Unclear	High
Weiner 1984[49]	Low	Unclear	Low	Low	Low	Unclear	High
Montenegro 1985[50]	Unclear	Unclear	Low	Low	High	High	High
Barrilleaux 2005[53 54]	Low	Low	Low	Low	Low	High	High
Hladunewich 2006[51]	Low	Low	Low	Low	High	High	High
Liu 2009[52]	High	High	High	High	High	Unclear	High
**UTERINE CURETTAGE**							
Magann 1993[59]	Low	Low	High	High	Unclear	Unclear	Low
Magann 1994[60]	Low	Unclear	High	High	Unclear	Unclear	Low
Gomez 2005[61]	Unclear	Unclear	High	High	Unclear	High	Low
Alkan 2006[62]	Unclear	Unclear	High	High	Low	High	High
Ragab 2013[15]	Low	Low	High	High	Low	Low	Low
Mallapur 2015[18]	Low	Unclear	High	High	Unclear	Unclear	High
**ORGANISATION OF CARE**							
York 1997[26]	Unclear	Low	High	High	Unclear	Unclear	High

For peer review only

Appendix S6b: Risk of bias in included cohort studies (n=7)

Study ID	Selection				Comparability[1]	Outcome		
	Representative-ness[2]	Selection of non-exposed[3]	Ascertainment of exposure[4]	Outcome of interest not present at start		Assessment[5]	F/U long enough	Adequacy of F/U[6]
**ANTIHYPERTENSIVE MEDICATIONS**								
Krebs 1956[43 44]	Low (a)	Low (a)	Unclear (d)	Low (Yes)	Low (a)	High (b)	Low (Yes)	Unclear (d)
Palot 1979[36]	Unclear (d)	Low (a)	Unclear (d)	Low (Yes)	High (Neither)	Unclear (d)	Low (Yes)	Unclear (d)
Shumard 2016[41]	Low (a)	Low (a)	Low (a)	Low (Yes)	High (Neither)	Low (a)	Low (Yes)	Low (a)
**OTHER DRUGS**								
Shigemitsu 2015[47]	Unclear (d)	Unclear (c)	Low (a)	Low (Yes)	High (Neither)	Low (a)	Low (Yes)	Unclear (d)
**UTERINE CURETTAGE**								
Salvatore 1967[58]	High (b)	High (b)	Low (a)	Low (Yes)	High (Neither)	Low (a)	Low (Yes)	Low (a)
Gocmen 1996[57]	Unclear (d)	Unclear (c)	Unclear (d)	Low (Yes)	High (Neither)	Unclear (d)	High (No)	Unclear (d)
**ORGANISATION OF CARE**								
Bibbo 2014[33]	Unclear (d)	Unclear (c)	Unclear (d)	Low (Yes)	High (Neither)	Unclear (d)	Low (Yes)	Unclear (d)

For peer review only

[1] (a) study controls for most important factor; (b) study controls for any additional factor
[2] (a) truly representative of the average in the community; (b) somewhat representative of the average in the community; (c) selected group of users e.g. nurses, volunteers; (d) no description of the derivation of the cohort
[3] (a) drawn from the same community as the exposed cohort; (b) drawn from a different source; (c) no description of the derivation of the non-exposed cohort
[4] (a) secure record (e.g. surgical record); (b) structured interview; (c) written self-report; (d) no description
[5] (a) independent blind assessment; (b) record linkage; (c) self-report; (d) no description
[6] (a) complete follow-up; (b) subjects lost to follow-up unlikely to introduce bias (>90% follow-up rate); (c) follow up rate <90% and no description of those lost; (d) no statement

BMJ Open

Postpartum management of hypertensive disorders of pregnancy: a systematic review

Journal:	*BMJ Open*
Manuscript ID	bmjopen-2017-018696.R1
Article Type:	Research
Date Submitted by the Author:	02-Oct-2017
Complete List of Authors:	Cairns, Alexandra; University of Oxford Medical Sciences Division, Nuffield Department of Primary Care Health Sciences<br>Pealing, Louise; University of Oxford Medical Sciences Division, Nuffield Department of Primary Care Health Sciences<br>Duffy, James; University of Oxford Medical Sciences Division, Nuffield Department of Primary Care Health Sciences<br>Roberts, Nia; University of Oxford, UK, Bodleian Health Care Libraries,<br>Tucker, Katherine; University of Oxford, Nuffield Department of Primary Care<br>Leeson, Paul; University of Oxford, Division of Cardiovascular Medicine<br>MacKillop, Lucy; Oxford University Hospitals NHS Trust, Nuffield Department of Obstetrics & Gynaecology<br>McManus, Richard; University of Oxford, Dept of Primary Care Health Sciences
**Primary Subject Heading**:	Obstetrics and gynaecology
Secondary Subject Heading:	Evidence based practice
Keywords:	Preeclampsia, Gestational hypertension, Postpartum, Hypertensive disorders of pregnancy, Antihypertensive medication, Systematic review

SCHOLARONE™
Manuscripts

Postpartum management of hypertensive disorders of pregnancy: a systematic review

Dr Alexandra E. Cairns MA Hons (Oxon) BMBCh MRCOG[1], Dr Louise Pealing MA Hons (Cantab) MSc MBBS MRCP MRCGP[1], Dr James M.N. Duffy MBChB MRes BSc (Hons) PG HCL[1], Nia Roberts MSc Econ[2], Dr Katherine L. Tucker PhD[1], Professor Paul Leeson PhD MBBChir BSc (Hons) FRCP[3], Dr Lucy H. Mackillop MA (Oxon) BMBCh FRCP[4], Professor Richard J. McManus PhD MA MBBS FRCGP FRCP[1]

[1] Nuffield Department of Primary Care Health Sciences, University of Oxford, Oxford, United Kingdom.
[2] Knowledge Centre, Bodleian Libraries, University of Oxford, Oxford, United Kingdom.
[3] Cardiovascular Clinical Research Facility, Division of Cardiovascular Medicine, Radcliffe Department of Medicine, University of Oxford, Oxford, United Kingdom.
[4] Nuffield Department of Obstetrics and Gynaecology, University of Oxford, Oxford, United Kingdom.

Corresponding author: Dr Alexandra E. Cairns BMBCh MA Hons (Oxon) MRCOG

alexandra.cairns@phc.ox.ac.uk

+44 (0) 1865 617960

Nuffield Department of Primary Care Health Sciences, University of Oxford, Radcliffe Primary Care, Radcliffe Observatory Quarter, Woodstock Road, Oxford, OX2 6GG, United Kingdom.

Short title: Postnatal Hypertension Management

Competing interests statement: None of the authors has any conflicts of interest to declare.

For peer review only - http://bmjopen.bmj.com/site/about/guidelines.xhtml

Abstract

Objectives

Hypertensive disorders of pregnancy (HDP) affect one in ten pregnancies and often persist postpartum when complications can occur. We aimed to determine the effectiveness and safety of pharmacologic interventions, other interventions, and different care models for postpartum hypertension management.

Design

A systematic review was undertaken. Nine electronic databases, including Medline, were searched from inception to 16/03/2017. After duplicate removal, 4,561 records were screened. Two authors independently selected studies, extracted study characteristics and data, and assessed methodological quality.

Setting

Randomised controlled trials, case-control studies, and cohort studies from any country and healthcare setting.

Participants

Postnatal women with HDP.

Interventions

Therapeutic intervention for management of hypertension, compared with another intervention, placebo, or no intervention.

Primary and secondary outcome measures

Outcome data were collected for maternal mortality and severe morbidity; systolic, diastolic and mean arterial blood pressure (BP) control; and safety data. Secondary outcome data collected included the length of postnatal hospital stay and laboratory values.

Results

39 studies were included (n=2,901). Results were heterogeneous in terms of intervention, comparison and outcome requiring a narrative approach. There were insufficient data to recommend any single pharmacologic intervention. 18 studies reported calcium-channel blockers, vasodilators and beta-blockers lowered BP postpartum. 12 of these reported safety data. Limited data existed regarding management in the weeks following hospital discharge. Neither loop diuretics (three studies) nor corticosteroids (one study) produced clinical benefit.

For peer review only - http://bmjopen.bmj.com/site/about/guidelines.xhtml

Uterine curettage significantly reduced BP over the first 48 hours postpartum (range 6-13mmHg) compared to standard care (eight studies), with safety data only reported by 4/8 studies.

Conclusion

There was insufficient evidence to recommend a particular BP threshold, agent, or model of care but three classes of antihypertensive appeared variably effective. Further comparative research, including robust safety data, is required. Curettage reduced BP, but without adequate reporting of harms, so cannot currently be recommended.

Strengths and limitations of this study

- All types of intervention for the management of postpartum hypertension – medical, surgical and organisation of care – were eligible for inclusion in this review.
- Randomised controlled studies plus other experimental study designs (cohort studies, case-control studies and quasi-randomised studies) were included and no limitations were imposed in terms of language or publication date, resulting in a comprehensive review.
- This review highlights significant evidence gaps, demonstrating that further comparative research is required, particularly to clarify postpartum antihypertensive selection.
- Although 39 studies were included, the majority had a high risk of bias such that the evidence provided by this review is of low quality.
- The 39 studies reported a broad range of heterogeneous outcomes, limiting meaningful comparison.

Keywords

Preeclampsia, gestational hypertension, postpartum, hypertensive disorders of pregnancy, antihypertensive medication, systematic review

Abbreviations

BP Blood pressure

For peer review only

For peer review only - http://bmjopen.bmj.com/site/about/guidelines.xhtml

HDP Hypertensive disorders of pregnancy

MAP Mean arterial pressure

NICE National Institute of Health and Care Excellence

RCT Randomised controlled trial

SSRI Selective serotonin reuptake inhibitor

For peer review only

For peer review only - http://bmjopen.bmj.com/site/about/guidelines.xhtml

Introduction

Hypertensive disorders of pregnancy (HDP) often persist following delivery,[1] and occasionally arise de novo postpartum.[2] In both scenarios adverse events can occur during this period. Approximately one-third of eclampsia occurs postpartum, nearly half beyond 48 hours after childbirth.[3-5] Half of the women who sustain an intracerebral haemorrhage in association with preeclampsia do so following birth.[6] Women may enter the postnatal period requiring large doses of antihypertensive medication, but the majority will be treatment-free by three to six months.[1 7] This rapidly changing blood pressure (BP) poses a challenge in terms of appropriate antihypertensive selection and dose adjustment.

The National Institute for Health and Care Excellence (NICE) recommends frequent postnatal BP monitoring for women with both preeclampsia (every one to two days for two weeks) and gestational hypertension (at least once between day three and five).[8] The guideline stipulates thresholds for the increase or commencement (≥150/100mmHg) and the reduction or cessation (consider <140/90mmHg and reduce <130/80mmHg) of antihypertensive medication after birth. However, little detail is provided about frequency or proportion of dose reduction or how to manage multiple medications.[8] The American College of Obstetricians and Gynecologists recommend that BP be monitored in hospital (or with an equivalent level of outpatient surveillance) for 72 hours after birth, and checked again seven to ten days postpartum (sooner if a woman is symptomatic).[9] In line with NICE, they propose treating BP when ≥150/100mmHg, but add this should be on two measures, four to six hours apart. They make no suggestion regarding BP thresholds for medication reduction, implying uncertainty about when to decrease or stop treatment.

A Cochrane review (search date January 2013) evaluated medical interventions for prevention and treatment of postnatal hypertension. This was limited to randomised controlled trials (RCTs) and included only nine studies.[10] Given the paucity of evidence available, due to Cochrane's restriction to randomised trials alone, we have undertaken an updated systematic review of the postpartum management of hypertension in women with HDP with a broader scope: including the full range of interventions studied, and incorporating cohort and case-control studies, alongside RCTs. Our specific questions were: [1] How should BP be monitored in women with HDP postpartum? [2] What BP thresholds should be used for antihypertensive treatment initiation, adjustment and cessation postpartum? [3] Which antihypertensive medication(s) should be used in postpartum in

For peer review only - http://bmjopen.bmj.com/site/about/guidelines.xhtml

women with HDP? [4] What are the benefits and harms of other therapeutic interventions for women with HDP postpartum?

Material and methods

A protocol, with explicitly defined objectives, study selection criteria, and approaches to assessing study quality, outcomes and statistical methods, was developed (Appendix S1). This was registered with PROSPERO: International Prospective Register of Systematic Reviews (CRD42015015527) and is available online (http://www.crd.york.ac.uk/PROSPERO/display_record.asp?ID=CRD42015015527). We followed the guidelines for meta-analyses and systematic reviews outlined by the Preferred Reporting Items for Systematic Reviews and Meta-Analyses (PRISMA) statement (Appendix S2).[11]

A systematic literature review was undertaken to capture evidence from human studies regarding postpartum hypertension management in women with HDP, without restriction by language or publication date (Appendix S1). We searched the following databases, from inception to 16/03/2017: Cochrane Database of Systematic Reviews (CDSR), Database of Abstracts of Reviews of Effects (DARE) and Cochrane Central Register of Controlled Trials (CENTRAL), Cumulative Index to Nursing and Allied Health Literature (CINAHL), Embase, Medline, PsycINFO, Science Citation Index, Science (Web of Science Core Collection), Social Science Citation Index & Conference Proceedings Citation Index. We hand-searched reference lists and contacted relevant experts for potentially relevant studies, which might have been missed by electronic searches.[12]

We included RCTs, quasi-randomised studies, case-control studies, prospective and retrospective cohort studies, assessing interventions for hypertension management postpartum in women with HDP (gestational hypertension, pre-eclampsia, chronic hypertension and super-imposed pre-eclampsia) arising both during pregnancy and de novo in the postnatal period. Consistent with guidance from Cochrane, conference abstracts were included.[5]

Two reviewers (AC/LP) independently screened the titles and abstracts, and then critically reviewed the full text of selected studies to assess eligibility. Discrepancies were resolved by discussion before independent extraction of relevant data by the two reviewers. For trials with multiple intervention arms, we extracted data from eligible comparison arms. Data were

For peer review only - http://bmjopen.bmj.com/site/about/guidelines.xhtml
For peer review only

extracted for the primary and secondary outcomes outlined in Table 1. Due to the heterogeneous nature of these studies, a narrative synthesis was undertaken.

Two reviewers (AC/LP) independently assessed each trial's methodological quality using the Cochrane Collaboration's tool for assessing the risk of bias in RCTs,[13] and the Newcastle-Ottawa scale for case-control and cohort studies.[14] A global assessment of bias across trials was made.

Results

Our searches yielded 7,105 records and after excluding duplicates, 4,561 titles and abstracts were screened (Figure 1). 80 full-text articles were assessed: 35 articles were excluded (Appendix S3). 45 articles, representing 39 studies (32 randomised trials, two prospective cohort studies, and five retrospective cohort studies) reporting data from 2,901 postnatal participants met our inclusion criteria (Appendix S4). 9/39 (23%) were published only as conference abstracts. No further details were made available following author contact.

A range of interventions was assessed including antihypertensive medications (18 studies, n=982), loop diuretics (four studies, n=503), parenteral steroids (one study, n=157), other medications (six studies, n=188), uterine curettage (eight studies, n=837) and novel models of care (two studies, n=234). 9/39 (23%) included ≥100 participants, and only two studies included ≥200 participants.[15 16] Four were from lower-middle-income settings[15 17-19] (classified according to the United Nations[20]), and 13/39 (33%) studies had follow-up periods longer than seven days (Appendix S4). Only 5/39 (13%) and 7/39 (18%) studies, respectively, reported maternal mortality or major maternal morbidity, and whilst the majority of studies did report some measure of BP control, three did not (Tables 2a&b). 19/39 (49%) studies reported safety data (Tables 2a&b).

5/39 (13%) studies (all evaluating antihypertensive medications) involved mixed antenatal and postnatal populations[17 21-24]. Authors were contacted to request their dataset for the postnatal participants, but no data were made available. 6/39 (15%) studies included participants with chronic hypertension alongside women with de novo HDP (gestational hypertension or pre-eclampsia).[22 23 25-31] 12/39 (31%) studies included women with eclampsia – in one all participants were eclamptic (Appendix S5).[17]

30/32 (94%) included RCTs were judged to be at high overall risk of bias, by both reviewers, according to the Cochrane tool, 23/32 (72%) for multiple domains. Only 2/32 (6%) were

For peer review only - http://bmjopen.bmj.com/site/about/guidelines.xhtml

thought to be clearly at low risk of bias.[29-32] All included cohort studies were deemed to have a high risk of bias in at least one domain of the Newcastle-Ottawa scale (Appendix S6).

How should blood pressure be monitored postpartum in women with hypertensive disorders of pregnancy?

No studies specifically addressed the frequency or method of postnatal BP monitoring. Two evaluated the impact of postpartum care organisation (n=234), using the postnatal readmission rate as their primary outcome (Appendix S4). Neither reported maternal mortality or morbidity, safety data nor any measure of BP control (Table 2b).[26 33]

One assessed introduction of a specialised postpartum clinic (no further details were given) and demonstrated an increased postnatal readmission and triage visit rate (22% intervention group, 9% control group: difference 13%, $p<0.04$) although 86% occurred before a participant was seen in the clinic.[33] The second study evaluated specialist nurse follow-up, including home visits and telephone contact, and reported no significant difference in the postnatal readmission rate compared to standard care.[26]

What blood pressure thresholds should be used for antihypertensive treatment initiation, adjustment and cessation postpartum?

No relevant studies identified.

Which antihypertensive medication(s) should be used postpartum in women with hypertensive disorders of pregnancy?

14 randomised trials (n=645), one quasi-randomised trial (n=15), and three retrospective cohort studies (n=322) evaluated antihypertensive medications (Appendix S4). Only three studies reported maternal mortality,[29-31 34 35] and three reported maternal morbidity: no differences between groups were reported (Table 2a).[29-31 35 36] 12 studies reported safety data, in comparisons between multiple classes of antihypertensive agents (Table 2a): no clear differences were established, although one study found a greater number of minor side effects reported with oral nifedipine than with oral labetalol.[27 28]

The vast majority of included studies evaluated either acute control of severe hypertension (7/18, 39%), or BP control in the few days after delivery, whilst women remained hospital inpatients (8/18, 44%). Only three studies, two published only as conference abstracts, evaluated BP control in the weeks and months following hospital discharge.[25 27 28 37]

For peer review only - http://bmjopen.bmj.com/site/about/guidelines.xhtml

Calcium-channel blockers

Three small studies examined oral nifedipine (n=135): nifedipine resulted in a greater decrease in MAP 18-24 hours after childbirth than placebo (intervention group 93.9±1.6mmHg, control group 100.2±2.6mmHg, difference 6.3mmHg, *p*<0.05), but not at other time points to 48 hours (one RCT, n=31).[32] Nifedipine controlled severe hypertension to <160/100mmHg more quickly than labetalol (intervention group 25.1±13.6 minutes, control group 43.6±25.4 minutes: difference 18.5 minutes, *p*=0.002; one RCT, n=21).[21] A single RCT (n=83), reported no significant difference in time taken to control BP to <150/100mmHg when comparing nifedipine with methyldopa.[34]

Vasodilators

Six studies looked at the use of vasodilators (n=252). All utilised hydralazine via a range of administration routes. Bolus intravenous hydralazine controlled severe hypertension more quickly than continuous infusion (intervention group 65.23±23.38 minutes, control group 186.36±79.77 minutes: difference -121.13 minutes, *p*<0.001); one quasi-randomised study, n=15 (postnatal)).[17] Intramuscular hydralazine produced a more significant improvement in MAP at six hours than intravenous methyldopa (intervention group 104.5mmHg, control group 112mmHg: difference -7.5mmHg *p*=0.0057) but not at other time points to 24 hours (one RCT, n=26).[38 39] There was no difference in BP control when comparing oral hydralazine with oral nifedipine (one RCT, n=38), or intravenous labetalol (one RCT, n=82).[35 40]

Bolus diazoxide was significantly more effective in achieving a target BP of ≤140/90mmHg than intravenous hydralazine (intervention group 67%, control group 43%: RR 0.64, 95% CI 0.46-0.89; one RCT, n=37 (postnatal)).[23] One retrospective cohort study did not present any statistical analysis.[36]

Beta-blockers

Five studies assessed the efficacy of beta-blockers (four RCTs and one retrospective cohort study, n=305). Two RCTs compared intravenous labetalol with intravenous hydralazine/dihydralazine: one involved only six postnatal women and presented no statistical analysis of the data.[24] The other found a significantly greater mean maximal decrease in MAP with intravenous labetalol (intervention group 25.5±11.2mmHg, control group 33.3±13.2mmHg: difference -7.8mmHg, *p*=0.02; one RCT, n=32 (postnatal)).[22] Results conflicted regarding whether oral labetalol was more or less effective than oral

For peer review only - http://bmjopen.bmj.com/site/about/guidelines.xhtml

nifedipine: a cohort study reported that labetalol controlled BP less rapidly than nifedipine (intervention group 2.7 days, control group 1.7 days: difference 1.0 days, p=0.0031; one retrospective cohort study, n=128).[41] However, this result was not replicated by an RCT, where the time to BP control was similar in the two groups (n=50).[27 28] Neither study demonstrated a difference in the postnatal length of stay (n=178). Timolol was effective in decreasing diastolic BP on day one postnatal when compared with methyldopa (intervention group 88.7mmHg, control group 93.8mmHg: difference -5.1mmHg; p<0.05; one RCT, n=80).[42]

Other antihypertensive medications

No statistically significant difference was found between oral clonidine and oral captopril in the incidence of episodes of severe hypertension postpartum (one RCT, n=90).[29-31] Two RCTs evaluating indapamide versus methyldopa found no difference in BP control over 6-12 months postpartum (n=60).[25 37] One retrospective cohort study (n=140) compared reserpine with phenobarbital: the results suggested that reserpine might achieve faster and greater BP reduction (data extracted from graphs; no statistical analysis). No adverse events were reported in the intervention group.[43 44]

What are the benefits and harms of other therapeutic interventions for women with hypertensive disorders of pregnancy postpartum?

Loop Diuretics

Four RCTs (n=503) examined loop diuretics versus placebo or usual care in postpartum hypertension management in women with HDP. None reported maternal mortality or safety data. Only two reported major maternal morbidity, neither demonstrating a difference between groups (Table 2b).[16 19]

One RCT (n=120) reported significant improvement in the primary outcome of mean systolic and diastolic BP with oral furosemide versus placebo (magnitude of difference or time points of measurements not stated, p<0.001).[45] This was not the case in the other placebo-controlled RCT, which found no significant difference (n=19).[46] Two further RCTs (n=364) found no significant difference in BP control with oral furosemide versus usual care.[16 19] In one of these, subgroup analysis of women with severe preeclampsia (n=70) found women who received oral furosemide had a significantly lower systolic BP day 2 postpartum (intervention group 142±13mmHg, control group 153±19mmHg: difference -11mmHg, p<0.004), but not at other time points.[16] In the other trial (n=100), furosemide reduced the need for additional

For peer review only - http://bmjopen.bmj.com/site/about/guidelines.xhtml
For peer review only

antihypertensive treatment during the three days of therapy (intervention group 8.0%, control group 26.0% difference 18%, p=0.017), but this difference did not persist to hospital discharge.[19]

Other drugs

Five RCTs, one quasi-randomised study and one retrospective cohort study investigated the utility of different drug classes in HDP postpartum (Appendix S5). Three studies reported safety data, but only one reported maternal mortality, demonstrating no difference between groups,[47] and none reported major maternal morbidity (Table 2b).

Three small, crossover RCTs examined the use of selective serotonin receptor inhibitors (SSRIs) compared with placebo (n=55). All studies showed a significant reduction in BP with SSRIs compared to placebo (range 25.6 – 34mmHg).[48-50] These data suggest efficacy for this drug class in hypertension management but do not provide any information regarding relative effectiveness compared to standard antihypertensive drugs. Only one study reported safety data: although no statistical analysis was performed, there were a number of side effects reported in the intervention group.[49]

Two studies evaluated alternative therapies (n=117): there was no difference in BP control with L-arginine supplementation compared with placebo (one RCT, n=45).[51] One reported accelerated recovery of albuminuria with the administration of shengkangbao (Chinese herbal medicine) versus placebo (one quasi-randomised study, n=72). However, the clinical relevance of this outcome is uncertain, there was no difference between groups in the secondary outcomes of systolic BP, diastolic BP or serum creatinine and no safety data were reported.[52]

A single RCT assessed corticosteroids in the management of severe preeclampsia postpartum (n=157).[53 54] No difference was demonstrated between groups in the primary outcome of antihypertensive medication requirement, or in the secondary outcomes of mean arterial pressure (MAP) or need for critical care admission, and no safety data were reported. There were small, statistically significant differences found in some laboratory values (platelet count, lactate dehydrogenase and aspartate transaminase). However, the authors acknowledged that the absolute differences were too small to be clinically relevant.[53]

A very small retrospective cohort study suggested an improvement in MAP with the addition of carperitide (atrial natriuretic peptide) to standard therapy (n=16), and no adverse effects

For peer review only - http://bmjopen.bmj.com/site/about/guidelines.xhtml

related to the intervention were reported.[47] However, the magnitude of the difference was not published, and the study was too small to draw any firm conclusions.

Uterine curettage

Six RCTs and two prospective cohort studies (n=837) have explored the role of uterine curettage in postpartum hypertension management. Uterine curettage is a similar process to that used in the surgical management of miscarriage: the lining of the uterus is scraped after completion of the third stage of labour in order to maximise placental tissue removal. This may be under direct vision following caesarean section, or via the transcervical route following vaginal birth. The latter approach may be ultrasound-guided and necessitates some form of anaesthesia. The theory underlying this intervention is that gestational hypertension and preeclampsia are placenta-mediated, and therefore ensuring complete evacuation of the uterus following childbirth may accelerate recovery.[55 56]

Seven studies explicitly stated they included both participants who delivered vaginally and those delivered by caesarean: four reported numbers undergoing vaginal delivery (n=248) and caesarean (n=321). One made no comment about the mode of birth.[57] Only one study reported maternal mortality: no difference between groups.[15] Two reported major maternal morbidity, but neither performed any statistical analysis (Table 2b). However, both studies did suggest a reduction in the absolute number of eclamptic seizures in the curettage group compared to no intervention.[15 58] In one, however, there was a relevant difference between the study groups: 28/28 (100%) in the control group were eclamptic at enrolment, compared to 9/20 (45%) in the intervention group.[58] Four studies reported safety data, with none reporting any complications related to the intervention (Table 2b).[59-62]

All eight studies compared curettage with standard care (i.e. no additional intervention), and all suggested that uterine curettage resulted in a significantly lower BP.[15 18 57-62] One of these had two control groups: standard care, and oral nifedipine; when compared to oral nifedipine, no difference was noted with curettage.[60]

Five studies reported the magnitude of the difference in MAP between curettage and standard care: range 6-13mmHg.[15 18 59 60 62] Only two of these reported BP data beyond 24 hours postpartum: one RCT reported a significantly lower MAP at 48 hours with curettage (intervention group 104mmHg, control group 113mmHg, difference 9mmHg, p=0.0017; n=45),[60] but the other RCT demonstrated no significant difference in MAP at 48 hours (n=420).[15]

For peer review only - http://bmjopen.bmj.com/site/about/guidelines.xhtml

One study demonstrated that a greater proportion of the intervention group attained the target BP of <140/90mmHg at 24 (intervention group 9/20 (45%), control group 3/28 (11%): difference 34%, no *p*-value quoted) and 48 hours postpartum (intervention group 14/20 (70%), control group 8/28 (29%): difference 41%, no *p*-value quoted).[58] Two studies did not present the size of the difference between groups.[57 61]

Discussion

This review found evidence demonstrating that calcium-channel blockers, vasodilators and beta-blockers lower BP postpartum, but no clear answer to which was most effective and should, therefore, be preferentially prescribed. All but two studies examined the acute control of severe hypertension or short term BP control whilst women remained in hospital postpartum,[25 37] and so provide little guidance about prescription in the weeks after discharge. Moreover these both examined thiazide diuretics, not recommended in the UK for use whilst breastfeeding.[8] Complete safety data were limited across trials, as were data regarding objective clinical outcomes and two further studies examined antihypertensive agents not recommended for use postpartum in the UK (methyldopa and reserpine).[63 64] One trial evaluated captopril at a much higher daily dose than the UK recommended daily starting dose.[64]

Uterine curettage is not currently recommended, due to safety concerns regarding additional anaesthetic and operative risks, and the availability of alternative treatments to lower BP, particularly in the context of vaginal birth.[65] However, the included studies consistently demonstrated that uterine curettage improved BP control versus standard care,[15 18 57-62] with one reporting an equivalent effect to oral nifedipine.[60] Amongst the limited safety data none reported an excess complication rate (infection or uterine damage) with curettage, but given the low incidence of operative complications, the total population (n=837) was likely insufficient to adequately address potential competing risks. Furthermore, these studies did not demonstrate any impact from curettage on maternal mortality or severe morbidity and concerns exist about some studies' methodology. The evidence reviewed is insufficient to recommend incorporation of this intervention into routine clinical practice.

Four trials evaluating loop diuretics failed to provide conclusive evidence of benefit. Three produced non-significant results in their main analysis,[16 19 46] and the single conference abstract which did suggest better BP control with oral furosemide, did not publish the magnitude of the difference, rendering it difficult to assess the clinical relevance.[45] In contrast

For peer review only - http://bmjopen.bmj.com/site/about/guidelines.xhtml

to the Cochrane review, we conclude that, at present, there is no evidence to support the routine use of diuretics postpartum.[10]

We found no adequate evidence to support alternative medications or a particular care model in the management of HDP postpartum. SSRIs substantially reduced BP versus placebo,[48-50] but no published data was identified comparing their efficacy with standard antihypertensive treatment, making it difficult to draw meaningful conclusions about their clinical application. Neither study evaluating postpartum care organisation reported maternal mortality or morbidity, or any measure of BP control, with both selecting postnatal readmissions as their primary outcome. An increased postnatal readmission rate, however, may not necessarily reflect harm: it might instead suggest that a particular model of care can better detect problems in the community and admit appropriately, ultimately resulting in a lower risk to patients.

In light of the heterogeneous nature of research in this field, when designing this review, we included all interventions targeting hypertension management, but not end-organ complications, including eclampsia. Therefore, trials evaluating magnesium sulphate were outside the scope of this review. We acknowledge the relevance of this therapy in women with severe pre-eclampsia, especially in the immediate postnatal period, and a Cochrane review suggests there is no uncertainty regarding the effectiveness of this therapy.[66]

A strength of this review is that cohort studies, case-control studies and quasi-randomised studies were eligible in addition to RCTs, and no language or date restrictions were imposed, resulting in a comprehensive review that provides evidence suggesting significant research gaps, consistent with the findings from the Cochrane review (2013).[10] The applicability of the findings and recommendations from this review are restricted by the low quality of included studies: both reviewers judged the vast majority to be at high overall risk of bias. Nearly one-quarter of the included studies were published only as conference abstracts, and therefore not subjected to peer review. Data extraction was restricted to the information provided in the abstracts (no authors provided additional data upon request). These were limiting factors in our analysis, but we nonetheless felt it was important to include these studies for completeness, especially given the paucity of evidence that exists in this field. A further justification for their inclusion is that half of the trials reported in conference abstracts never reach full publication, and positive trials are more likely to be published than negative ones,[67] which has the potential to skew the results of a review if they are omitted.

For peer review only - http://bmjopen.bmj.com/site/about/guidelines.xhtml
For peer review only

A further limitation of this review is that the majority of identified studies did not report substantive clinical outcomes such as maternal mortality, morbidity or harms. Without these, it is difficult to define properly the potential role of proposed interventions in clinical practice. The incidence of adverse maternal and neonatal outcomes, particularly in high resource settings, is low meaning adequately powering studies for real outcomes of interest is financially demanding. Therefore researchers often employ surrogate outcomes. Additionally, the range of outcomes reported in included studies was broad and inconsistent, with BP changes in particular being measured in a variety of different ways, further limiting the comparability of trials. Increasingly, core-outcome sets are being produced, with a view to trials reporting as standard, a minimum set of outcomes that are clinically meaningful and important to patients.[68] We hope in future this would enhance our ability to synthesize results from different studies to produce high-quality evidence. There is consensus about trying to move away from surrogate outcomes, for example time to BP control, as they cannot effectively substitute for clinically important outcomes. An important and clinically meaningful end point should measure how a patient feels, functions, or survives.

The Cochrane review included only nine randomised trials (author names in bold in Appendix S4). We believe our review adds to this, as an additional 30 studies are included (19 pre-dating the Cochrane search, and 11 subsequent to it), providing a current and complete summary of all available research in the field. The contrast between the scales of the two reviews highlights a lack of high quality evidence, despite a reasonably high number of research studies being conducted to answer the question about how hypertension should be managed postpartum in women with HDP. In future, studies need to be more robust and better designed to address the research questions adequately. Furthermore, in spite of these extensions, the body of evidence identified was substantially smaller than that underpinning antenatal hypertension management: eighteen studies (n=982), not restricted to RCTs, evaluated antihypertensive medications postpartum. Furthermore, the size of all but a few individual studies was small. In comparison, a Cochrane review (2014) evaluating antihypertensive medication for mild to moderate hypertension in pregnancy included 49 RCTs (n=4,723).[69] Moreover, the quantity and quality of evidence supporting the management of HDP is vastly less than that available for essential hypertension outside pregnancy, where individual RCTs commonly involve several thousand participants.[70]

This review demonstrates a lack of good quality evidence for postpartum hypertension management, emphasising the need for further RCTs directly comparing different

For peer review only - http://bmjopen.bmj.com/site/about/guidelines.xhtml

434 antihypertensive agents, BP thresholds for medication adjustment and different models of care, with outcome measures other than postnatal readmissions. We believe the studies examining uterine curettage justify further research to evaluate clinically meaningful outcomes and procedural risks. It might be pragmatic to confine this to curettage at caesarean section, given concerns regarding surgical intervention after vaginal birth: an additional anaesthetic is not required; infection risk is lowered within a sterile surgical field compared to the transcervical route, and curettage under direct vision limits perforation risk. This might be beneficial in women with severe preeclampsia where BP control during pregnancy has been challenging despite multiple medications.[55]

For peer review only

For peer review only - http://bmjopen.bmj.com/site/about/guidelines.xhtml

Acknowledgements

Thanks to Dr Ly-Mee Yu, Dr Helen Cotton and Dr Victoria E Cairns for their assistance with translation.

Funding

The research was funded by the National Institute for Health Research (NIHR) Collaboration for Leadership in Applied Health Research and Care Oxford at Oxford Health NHS Foundation Trust and via a Research Professorship awarded to RMcM (NIHR-RP-02-12-015). The views expressed are those of the author(s) and not necessarily those of the NHS, the NIHR or the Department of Health.

Details of ethical approval

No ethical approval was required for this project.

Contribution to authorship

AC drafted the protocol with JD and drafted and piloted the data extraction sheet. These were reviewed by RMcM, LP, KT, LM and PL. NR and AC wrote the search strategy, and the online searches were conducted by NR. AC and LP reviewed the search results independently and carried out the data extraction. This manuscript was drafted by AC and reviewed by RMcM, JD, LP, NR, KT, LM and PL. AC will be the guarantor.

Data sharing statement

The data utilised in this systematic review originate from the research articles cited in this manuscript. These were collected from the published manuscripts. No additional unpublished data are available.

For peer review only

For peer review only - http://bmjopen.bmj.com/site/about/guidelines.xhtml

References

1. Podymow T, August P. Postpartum course of gestational hypertension and preeclampsia. *Hypertens Pregnancy* 2010;29(3):294-300. doi: 10.3109/10641950902777747

2. Goel A, Maski MR, Bajracharya S, et al. Epidemiology and Mechanisms of De Novo and Persistent Hypertension in the Postpartum Period. *Circulation* 2015;132(18):1726-33. doi: 10.1161/CIRCULATIONAHA.115.015721.

3. Chames MC, Livingston JC, Ivester TS, et al. Late postpartum eclampsia: a preventable disease? *Am J Obstet Gynecol* 2002;186(6):1174-7. doi: 10.1067/mob.2002.123824

4. Kayem G, Kurinczuk JJ, Spark P, et al. Maternal and obstetric factors associated with delayed postpartum eclampsia: a national study population. *Acta Obstet Gyn Scand* 2011;90(9):1017-23. doi: 10.1111/j.1600-0412.2011.01218.x

5. Watson DL, Sibai BM, Shaver DC, et al. Late postpartum eclampsia: an update. *South Med J* 1983;76(12):1487-9.

6. Martin JN, Jr., Thigpen BD, Moore RC, et al. Stroke and severe preeclampsia and eclampsia: a paradigm shift focusing on systolic blood pressure. *Obstet Gynecol* 2005;105(2):246-54. doi: 10.1097/01.AOG.0000151116.84113.56

7. Berks D, Steegers EA, Molas M, et al. Resolution of hypertension and proteinuria after preeclampsia. *Obstet Gynecol* 2009;114(6):1307-14. doi: 10.1097/AOG.0b013e3181c14e3e

8. National Institute for Health and Care Excellence. NICE Clinical Guideline 107: Hypertension in pregnancy: the management of hypertensive disorders during pregnancy, 2011.

9. ACOG Task Force on Hypertension in Pregnancy. Hypertension in Pregnancy: American College of Obstetricians and Gynecologists, 2013.

10. Magee L, von Dadelszen P. Prevention and treatment of postpartum hypertension. *Cochrane Database Syst Rev* 2013(4) doi: 10.1002/14651858.CD004351.pub3

11. Moher D, Shamseer L, Clarke M, et al. Preferred reporting items for systematic review and meta-analysis protocols (PRISMA-P) 2015 statement. *Syst Rev* 2015;4(1) doi: 10.1186/2046-4053-4-1

For peer review only - http://bmjopen.bmj.com/site/about/guidelines.xhtml

For peer review only

12. McManus RJ, Wilson S, Delaney BC, et al. Review of the usefulness of contacting other experts when conducting a literature search for systematic reviews. *BMJ* 1998;317:1562-3.

13. Higgins JPT, Altman DG, Gøtzsche PC, et al. The Cochrane Collaboration's tool for assessing risk of bias in randomised trials. *BMJ* 2011;343:d5928. doi: 10.1136/bmj.d5928

14. Wells GA, Shea B, O'Connell D, et al. The Newcastle-Ottawa Scale (NOS) for assessing the quality of nonrandomised studies in meta-analyses [Available from: http://www.ohri.ca/programs/clinical_epidemiology/oxford.htm accessed 14/07/2017 2017.

15. Ragab A, Goda H, Raghib M, et al. Does immediate postpartum curettage of the endometrium accelerate recovery from preeclampsia-eclampsia? A randomized controlled trial. *Arch Gynecol Obstet* 2013;288(5):1035-8. doi: 10.1007/s00404-013-2866-0

16. Ascarelli MH, Johnson V, McCreary H, et al. Postpartum preeclampsia management with furosemide: a randomized clinical trial. *Obstet Gynecol* 2005;105(1):29-33.

17. Begum MR, Quadir E, Begum A, et al. Management of hypertensive emergencies of pregnancy by hydralazine bolus injection vs continuous drip-a comparative study. *MedGenMed* 2002;4(4)

18. Mallapur A, Renuka B, Katageri G, et al. Role of postpartum curettage in recovery of severe preeclampsia and eclampsia patients-a randomised controlled trial. *Int J Gynaecol Obstet* 2015;131(Suppl 5):E191.

19. Veena P, Perivela L, Raghavan SS. Furosemide in postpartum management of severe preeclampsia: A randomized controlled trial. *Hypertens Pregnancy* 2017;36(1):84-9. doi: 10.1080/10641955.2016.1239735

20. United Nations Department of Economic and Social Affairs. World economic situation and prospects 2016. New York, United States of America: United Nations, 2016.

21. Vermillion ST, Scardo JA, Newman RB, et al. A randomized, double-blind trial of oral nifedipine and intravenous labetalol in hypertensive emergencies of pregnancy. *Am J Obstet Gynecol* 1999;181(4):858-61.

22. Mabie WC, Gonzalez AR, Sibai BM, et al. A comparative trial of labetalol and hydralazine in the acute management of severe hypertension complicating pregnancy. *Obstet Gynecol* 1987;70(3, Part 1):328-33.

For peer review only - http://bmjopen.bmj.com/site/about/guidelines.xhtml

23. Hennessy A, Thornton CE, Makris A, et al. A randomised comparison of hydralazine and mini-bolus diazoxide for hypertensive emergencies in pregnancy: the PIVOT trial. *Aust N Z J Obstet Gynaecol* 2007;47(4):279-85. doi: 10.1111/j.1479-828X.2007.00738.x

24. Garden A, Davey DA, Dommisse J. Intravenous labetalol and intravenous dihydralazine in severe hypertension in pregnancy. *Clin Exp Hypertens B* 1982;1(2-3):371-83.

25. Gaisin IR, Iskchakova AS, Shilina LV. Indapamide in the management of post-partum hypertension: A randomized, case-control study. *Eur Heart J* 2013;34(Suppl 1):271.

26. York R, Brown LP, Samuels P, et al. A randomized trial of early discharge and nurse specialist transitional follow-up care of high-risk childbearing women. *Nurs Res* 1997;46(5):254-61.

27. Sharma KJ, Greene N, Kilpatrick SJ. Oral labetalol compared to oral extended release nifedipine for persistent postpartum hypertension: A randomized controlled trial. *Am J Obstet Gynecol* 2016;214(Suppl 1):S27-8.

28. Sharma KJ, Greene N, Kilpatrick SJ. Oral labetalol compared to oral nifedipine for postpartum hypertension: A randomized controlled trial. *Hypertens Pregnancy* 2017;36(1):44-7. doi: 10.1080/10641955.2016.1231317

29. Noronha Neto C, Maia SSB, Katz L, et al. Clonidine versus Captopril for Severe Postpartum Hypertension: A Randomized Controlled Trial. *PLoS One* 2017;12(1) doi: 10.1371/journal.pone.0168124

30. Amorim MMR, Noronha Neto C, Maia SB, et al. Clonidine compared with captopril for severe postpartum hypertension. *Obstet Gynecol* 2015;125(5 Suppl 1):42S.

31. Katz L, Noronha Neto C, Maia S, et al. Clonidine versus captopril for severe postpartum hypertension: A randomized controlled trial. *Pregnancy Hypertens* 2015;5(Suppl 1):29-30. doi: 10.1016/j.preghy.2014.10.061

32. Barton JR, Hiett AK, Conover WB. The use of nifedipine during the postpartum period in patients with severe preeclampsia. *Am J Obstet Gynecol* 1990;162(3):788-92.

33. Bibbo C, Celi A, Thomas AM, et al. Does the addition of a specialized postpartum clinic improve the care of women with preeclampsia? *Obstet Gynecol* 2014;123(5 Suppl 1):39S.

For peer review only - http://bmjopen.bmj.com/site/about/guidelines.xhtml

34. Sayin NC, Altundag G, Varol FG. Efficacy of alpha-methyldopa and nifedipine in the treatment of postpartum hypertension. [Turkish] Postpartum hipertansiyon tedavisinde alfametildopa ve nifedipinin etkinligi. *J Turk Ger Gynecol Assoc* 2005;6(2):118-22.

35. Vigil-De Gracia P, Ruiz E, Lopez JC, et al. Management of severe hypertension in the postpartum period with intravenous hydralazine or labetalol: a randomized clinical trial. *Hypertens Pregnancy* 2007;26(2):163-71. doi: 10.1080/10641950701204430

36. Palot M, Jakob L, Decaux J. Arterial hypertensions of labor and the post-partum period. [French] Les Hypertensions Arterielles Du Travail Et Du Post-Partum. *Rev Fr Gynecol Obstet* 1979;74(3):173-6.

37. Ilshat Gaisin IR, Iskchakova AS, Shilina LV. Control of cardiovascular risk factors with ursodeoxycholic acid and indapamide in postpreeclamptic nursing mothers: Results from a randomized, case-control 1-year study. *Eur J Prev Cardiol* 2014;21(Suppl 1):S120. doi: 10.1177/2047487314534585

38. Griffis KR, Jr., Martin JN, Jr., Palmer SM, et al. Utilization of hydralazine or alpha-methyldopa for the management of early puerperal hypertension. *Am J Perinatol* 1989;6(4):437-41.

39. Martin JN, Griffis KR, Martin RW, et al. Early puerperal hypertension management: hydralazine vs methyldopa. *Clin Exp Hypertens B* 1988;8(2):431.

40. Walss Rodriguez RJ, Villarreal Ordaz F. Management of severe pre-eclampsia in the puerperium. Comparative study of sublingual nifedipine and hydralazine. *Ginecol Obstet Mex* 1991;59:207-10.

41. Shumard K, Yoon J, Huang C, et al. Peripartum anti-hypertensive choice affects time to blood pressure control in treating hypertensive disorders of pregnancy. *Am J Obstet Gynecol* 2016;214(1 Suppl):S378.

42. Fidler J, Smith V, De Swiet M. A randomized study comparing timolol and methyldopa in hospital treatment of puerperal hypertension. *Br J Obstet Gynaecol* 1982;89(12):1031-4.

43. Krebs A. Experience with serpasil and luminal in the management of pregnancy-and puerperal toxemias. *Gynaecologia* 1956;141(4):255-60.

44. Krebs A. Tests of reserpine and phenobarbital in therapy of toxicosis of pregnancy and puerperium. *Geburtshilfe Frauenheilkd* 1956;16(5):410-22.

For peer review only - http://bmjopen.bmj.com/site/about/guidelines.xhtml
For peer review only

45. Amorim M, Katz L, Cursino T, et al. Postpartum furosemide for accelerating recovery in women with severe preeclampsia: A randomized clinical trial. *Int J Gynaecol Obstet* 2015;131(Suppl 5):E195.

46. Matthews G, Gornall R, Saunders NJ. A randomised placebo controlled trial of loop diuretics in moderate/severe pre-eclampsia, following delivery. *J Obstet Gynaecol* 1997;17(1):30-2. doi: 10.1080/01443619750114040

47. Shigemitsu A, Akasaka J, Shigetomi H, et al. Use of carperitide for postpartum diuresis of severe preeclampsia. *Pregnancy Hypertens* 2015;5(1):145. doi: 10.1016/j.preghy.2014.10.297

48. Weiner CP, Socol ML. Serotonin (5HT) receptor blockade in puerperal pre-eclampsia. *Clin Exp Hypertens B* 1982;1(2-3):195.

49. Weiner CP, Socol ML, Vaisrub N. Control of preeclamptic hypertension by ketanserin, a new serotonin receptor antagonist. *Am J Obstet Gynecol* 1984;149(5):496-500.

50. Montenegro R, Knuppel RA, Shah D, et al. The effect of serotonergic blockade in postpartum preeclamptic patients. *Am J Obstet Gynecol* 1985;153(2):130-4.

51. Hladunewich MA, Derby GC, Lafayette RA, et al. Effect of L-arginine therapy on the glomerular injury of preeclampsia: a randomized controlled trial. *Obstet Gynecol* 2006;107(4):886-95. doi: 10.1097/01.AOG.0000207637.01824.fe

52. Liu Z, Wang X, Yan N. Treatment of albuminuria in gestational hypertension puerpera in the severe preeclampeia stage by TCM therapy for stasis-removing and diuresis. *Zhongguo Zhong Xi Yi Jie He Za Zhi* 2009;29(3):222-4.

53. Barrilleaux PS, Martin JN, Jr., Klauser CK, et al. Postpartum intravenous dexamethasone for severely preeclamptic patients without hemolysis, elevated liver enzymes, low platelets (HELLP) syndrome: a randomized trial. *Obstet Gynecol* 2005;105(4):843-8.

54. Barrilleaux PS, Martin Jr J, Klauser C, et al. Adjunctive intravenous dexamethasone in patients with severe preeclampsia not complicated by HELLP syndrome. *Am J Obstet Gynecol* 2003;189(6 Suppl):S94.

For peer review only - http://bmjopen.bmj.com/site/about/guidelines.xhtml

For peer review only

55. Caughey AB. Editorial Comment: Does Immediate Postpartum Curettage of the Endometrium Accelerate Recovery From Preeclampsia–Eclampsia? A Randomized Controlled Trial. *Obstet Gynecol Surv* 2014;69(3):121-2. doi: 10.1097/ogx.0000000000000040

56. Hunter CA, Jr., Howard WF, McCormick CO, Jr. Amelioration of the hypertension of toxemia by postpartum curettage. *Am J Obstet Gynecol* 1961;81(5):884-9.

57. Gocmen A, Yayla M, Ceylan Erden A, et al. The effect of postpartum curettage in severe preeclampsia and eclampsia in recovery (Ağır preeklampsı ve eklampsi in iyileşmesinde postpartum kuretajin etkisi). *Perinatoloji Dergisi* 1996;4(1):24.

58. Salvatore CA, Carduz E, Ciccivizzo E, et al. Postpartum curettage in severe toxemia with and without premature loosening of the placenta. *Matern Infanc (Sao Paulo)* 1967;26(3):275-86.

59. Magann EF, Martin JN, Jr., Isaacs JD, et al. Immediate postpartum curettage: accelerated recovery from severe preeclampsia. *Obstet Gynecol* 1993;81(4):502-6.

60. Magann EF, Bass JD, Chauhan SP, et al. Accelerated recovery from severe preeclampsia: uterine curettage versus nifedipine. *J Soc Gynecol Investig* 1994;1(3):210-4.

61. Gomez LM, De la Vega GA, Ludmir J, et al. Immediate postpartum curettage accelerates clinical recovery in severe preeclampsia. *Obstet Gynecol* 2005;105(4 Suppl 4):111S.

62. Alkan A, Tugrul S, Oral O, et al. Effects of postpartum uterine curettage on maternal well-being in severe preeclamptic patients. *Clin Obstet Gynecol* 2006;33(1):55-8.

63. Webster J, Koch HF. Aspects of tolerability of centrally acting antihypertensive drugs. *J Cardiovasc Pharmacol* 1996;27(Suppl 3):S49-54.

64. Joint Formulary Committee. British National Formulary (BNF). 71 ed: BMJ Publishing Group Ltd and Royal Pharmaceutical Society 2016.

65. Tan LK, de Swiet M. The management of postpartum hypertension. *Br J Obstet Gynaecol* 2002;109(7):733-6.

66. Duley L, Gulmezoglu AM, Henderson-Smart DJ, et al. Magnesium sulphate and other anticonvulsants for women with pre-eclampsia. *Cochrane Database Syst Rev* 2010(11) doi: 10.1002/14651858.CD000025.pub2

For peer review only - http://bmjopen.bmj.com/site/about/guidelines.xhtml

67. Scherer RW, Langenberg P, von Elm E. Full publication of results initially presented in abstracts. *Cochrane Database Syst Rev* 2007(2) doi: 10.1002/14651858.MR000005.pub3

68. Khan K. The CROWN Initiative: journal editors invite researchers to develop core outcomes in women's health. *Br J Obstet Gynaecol* 2014;121(10):1181-2. doi: 10.1111/1471-0528.12929

69. Abalos E, Duley L, Steyn DW. Antihypertensive drug therapy for mild to moderate hypertension during pregnancy. *Cochrane Database Syst Rev* 2014(2) doi: 10.1002/14651858.CD002252.pub3

70. National Institute for Health and Care Excellence. NICE Clinical Guideline 127: Hypertension in adults: diagnosis and management, 2011.

For peer review only

For peer review only - http://bmjopen.bmj.com/site/about/guidelines.xhtml

Tables and figures

Figure 1: PRISMA Flowchart

See separate document.

For peer review only

For peer review only - http://bmjopen.bmj.com/site/about/guidelines.xhtml

Table 1: Outcome measures

	Outcome measures	Timing
**Primary outcome(s)**	Maternal mortality<br>Maternal morbidity (ischaemic stroke, intracranial haemorrhage, eclamptic seizure; development of preeclampsia with severe features; postnatal complication requiring intervention)<br>Systolic blood pressure control<br>Diastolic blood pressure control<br>Mean arterial pressure control<br>Safety data (adverse events or maternal side effects)	Direct maternal deaths up to day 42 postpartum; later maternal deaths up to 1 year postpartum
**Secondary outcome(s)**	Critical care admission<br>Length of hospital stay following delivery<br>Postnatal readmission to secondary care<br>Antihypertensive medication requirement<br>Urine output<br>Laboratory values<br>Other as defined by study	

For peer review only

For peer review only - http://bmjopen.bmj.com/site/about/guidelines.xhtml

Table 2a: Primary outcome and safety data reporting in included studies (Antihypertensive medications, 18 studies)

Study ID	Intervention	Control	Primary outcome assessment: Maternal mortality	Maternal morbidity	SBP control	DBP control	MAP control	Safety data reporting	Results (for reported outcomes)
**CALCIUM CHANNEL BLOCKERS (3 studies)**									
Barton 1990[32]	Nifedipine (oral)	Placebo			● (yellow)	● (yellow)	● (green)		SBP control: no significant difference.<br>DBP control: no significant difference.<br>MAP control: improved in intervention group (difference 6.3mmHg, p<0.05).
Vermillion 1999[21]	Nifedipine (oral)	Labetalol (IV bolus)			● (green)	● (green)		● (yellow)	SBP control: improved in intervention group (difference in time to target BP 18.5 minutes, p=0.002).<br>DBP control: improved in intervention group (difference in time to target BP 18.5 minutes, p=0.002).<br>Safety: no significant difference. 1/25 intervention group became hypotensive.
Sayin 2005[34]	Nifedipine (oral)	Methyldopa (oral)	● (yellow)		● (yellow)	● (yellow)			Maternal mortality: no significant difference.<br>SBP control: no significant difference.<br>DBP control: no significant difference.
**VASODILATORS (6 studies)**									
Palot 1979[36]	Hydralazine (IV infusion) plus furosemide (IV bolus)	Clonidine (IV) plus furosemide (IV bolus)		● (black)					Maternal morbidity: no statistical analysis.
Griffis 1989[38 39]	Hydralazine (IM)	Methyldopa (IV bolus)					● (yellow)	● (yellow)	MAP control: no significant difference.<br>Safety: no significant difference. No side effects reported in either group.
Walss Rodriguez 1991[40]	Hydralazine (oral) plus nifedipine (oral, as required)	Nifedipine (oral, as required)			● (yellow)	● (yellow)			SBP control: no significant difference.<br>DBP control: no significant difference.
Begum 2002[17]	Hydralazine (IV bolus)	Hydralazine (IV infusion)				● (green)		● (yellow)	DBP control: improved in intervention group (difference in time to target DBP 121.1 minutes, p<0.001).<br>Safety: no significant difference. No side effects reported in either group.
Vigil de Gracia 2007[35]	Hydralazine (IV bolus)	Labetalol (IV bolus)	● (yellow)	● (yellow)	● (yellow)	● (yellow)		● (yellow)	Maternal mortality: no significant difference.<br>Maternal morbidity: no significant difference.<br>SBP control: no significant difference.<br>DBP control: no significant difference.<br>Safety: no significant difference. Small numbers of side effects reported in both groups.
Hennessy 2007[23]	Diazoxide (IV bolus)	Hydralazine (IV bolus)			● (green)	● (green)			SBP control: improved in intervention group (difference in percentage achieving target BP 23%, p<0.01).<br>DBP control: improved in intervention group (difference in percentage achieving target BP 23%, p<0.01).
**BETA BLOCKERS (5 studies)**									
Garden 1982[24]	Labetalol (IV infusion)	Dihydralazine (IV infusion)				● (black)		● (black)	DBP control: no statistical analysis.<br>Safety: no statistical analysis. 1/6 intervention group developed bronchospasm. 4/6 control group developed tachycardia and 1/6 developed oliguria. 4/6 control group – drug stopped due to a precipitous fall of DBP to 40-50mmHg.

For peer review only - http://bmjopen.bmj.com/site/about/guidelines.xhtml

Study ID	Intervention	Control	Primary outcome assessment: Maternal mortality	Maternal morbidity	SBP control	DBP control	MAP control	Safety data reporting	Results (for reported outcomes)
Fidler 1982[42]	Timolol (oral)	Methyldopa (oral)			🟢	🟡		⚫	SBP control: improved in intervention group (difference 5.1mmHg, p<0.05).<br>DBP control: no significant difference.<br>Safety: no statistical analysis. 1/40 intervention group became disorientated. 1/40 control group became hypotensive and 1/40 became drowsy.
Mabie 1987[22]	Labetalol (IV bolus)	Hydralazine (IV bolus)					🔴	⚫	MAP control: improved in control group (difference 7.8mmHg (p 0.02).<br>Safety: no statistical analysis. 1/40 intervention group developed scalp tingling. 2/20 control group developed headaches.
Shumard 2016[41]	Labetalol (oral)	Nifedipine (oral)			🔴	🔴			SBP control: improved in control group (difference in time to achieve target BP 1 day, p=0.0031).<br>DBP control: improved in control group (difference in time to achieve target BP 1 day, p=0.0031).
Sharma 2017[27 28]	Labetalol (oral)	Nifedipine (oral)			🟡	🟡		🟢	SBP: no significant difference.<br>DBP: no significant difference.<br>Safety: No major side effects reported in either group. Minor side effects more commonly reported in control group (20% intervention, 48% control, p=0.04).
**THIAZIDES (2 studies)**									
Gaisin 2013[25]	Indapamide (oral)	Methyldopa (oral)			🟡	🟡		⚫	SBP control: no significant difference.<br>DBP control: no significant difference.<br>Safety: no statistical analysis, no details reported.
Gaisin 2014[37]	Indapamide (oral) plus ursodeoxycholic acid (oral)	Methyldopa (oral)			🟡	🟡		🟡	SBP control: no significant difference.<br>DBP control: no significant difference.<br>Safety: no significant difference. No adverse events reported in either group.
**INDOLE ALKALOIDS (1 study)**									
Krebs 1956[43 44]	Reserpine (oral or IM)	Phenobarbital			⚫	⚫		⚫	SBP control: no statistical analysis.<br>DBP control: no statistical analysis.<br>Safety: no statistical analysis. No adverse events reported in intervention group, no comment on control.
**CENTRALLY-ACTING ALPHA-AGONISTS (1 study)**									
Noronha Neto 2016[29-31]	Clonidine (oral)	Captopril (oral)	🟡	🟡	🟢	🟢		🟡	Maternal mortality: no significant difference.<br>Maternal morbidity: no significant difference.<br>SBP control: improved in intervention group (difference in number of episodes of high BP (1.4, p<0.08).<br>DBP: improved in intervention group (difference in number of episodes of high BP (1.4, p<0.08).<br>Safety: no significant difference. Adverse reactions 18.6% intervention, 28.8% control, p=NS.

For peer review only

For peer review only - http://bmjopen.bmj.com/site/about/guidelines.xhtml

Table 2b: Primary outcome and safety data reporting in included studies (Loop diuretics, other drugs, uterine curettage and organisation of care, 21 studies)

Study ID	Intervention	Control	Primary outcome assessment					Safety data reporting	Results (for reported outcomes)
			Maternal mortality	Maternal morbidity	SBP control	DBP control	MAP control		
**LOOP DIURETICS (4 studies)**									
Matthews 1997[46]	Furosemide (oral)	Placebo					●		MAP control: no significant difference.
Ascarelli 2005[16]	Furosemide (oral)	No intervention		●	●	●			Maternal morbidity: no significant difference.<br>SBP control: no significant difference.<br>DBP control: no significant difference.
Amorim 2015[45]	Furosemide (oral)	Placebo			●	●	●		SBP control: improved in intervention group (difference not stated, p<0.001).<br>DBP control: improved in intervention group (difference not stated, p<0.001).<br>MAP control: improved in intervention group (difference not stated, p<0.001).
Veena 2017[19]	Furosemide (oral) + nifedipine (oral)	Nifedipine (oral)		●	●	●	●		Maternal morbidity: no significant difference.<br>SBP control: no significant difference.<br>DBP control: no significant difference.<br>MAP control: no significant difference.
**OTHER DRUGS (7 studies)**									
**Selective 5-HT antagonists**									
Weiner 1982[48]	R41468 (intravenous infusion)	Placebo					●		MAP control: improved in intervention group (difference 25.6mmHg, p<0.001).
Weiner 1984[49]	Ketanserin (IV infusion)	Placebo			●	●	●	●	SBP control: improved in intervention group (difference in SBP decline 34mmHg, p<0.001).<br>DBP control: improved in intervention group (difference in DBP decline 27mmHg, p<0.001).<br>MAP control: improved in intervention group (difference not stated, p<0.001)<br>Safety: No statistical analysis. 3/20 intervention group experienced blurred vision: 1 of these was hypotensive (responded to hydration). 1/20 intervention group experienced mild euphoria.
Montenegro 1985[50]	Ketanserin (IV bolus +/- infusion)	Placebo			●	●	●		SBP control: improved in intervention group (absolute difference not stated, p<0.001).<br>DBP control: improved in intervention group (absolute difference not stated, p<0.001).<br>MAP control: improved in intervention group (absolute difference not stated, p<0.001).
**Alternative therapies**									
Hladunewich 2006[51]	L-arginine (oral or IV bolus)	Placebo			●	●	●	●	SBP control: no significant difference.<br>DBP control: no significant difference.<br>MAP control: no significant difference.<br>Safety: no significant difference. No adverse events reported in either group.
Liu 2009[52]	Shengkangbao (oral or IV bolus)	No intervention			●	●			SBP control: no significant difference.<br>DBP control: no significant difference.

Page 29 of 31 Postnatal Hypertension Management
Version: 3.0 Date: 29/09/2017

For peer review only

Study ID	Intervention	Control	Primary outcome assessment					Safety data reporting	Results (for reported outcomes)
			Maternal mortality	Maternal morbidity	SBP control	DBP control	MAP control		
**Steroids**									
Barrilleaux 2005[53 54]	Dexamethasone (IV bolus)	Placebo					●		MAP control: no significant difference.
**Atrial natriuretic peptide**									
Shigemitsu 2015[47]	Carperitide (route not specified)	No intervention	●				●	●	Maternal mortality: no significant difference.<br>MAP control: no significant difference.<br>Safety: no significant difference. No adverse events reported in either group.
**UTERINE CURETTAGE (8 studies)**									
Salvatore 1967[58]	Uterine curettage	No intervention		●	●	●			Maternal morbidity: no statistical analysis.<br>SBP control: no statistical analysis.<br>DBP control: no statistical analysis.
Magann 1993[59]	Uterine curettage	No intervention					●	●	MAP control: improved in intervention group (difference at different time points to 24h postpartum 6-10mmHg, p<0.05).<br>Safety: no significant difference. No complications reported from intervention (follow-up to 7 weeks postpartum).
Magann 1994[60]	Uterine curettage	Nifedipine (oral) or no intervention					●<br>●	●	MAP control: no significant difference between intervention and oral nifedipine; improved in intervention group compared to no intervention (difference at 8-48h postpartum 9-13mmHg, p=0.0017).<br>Safety: no significant difference. No complications/side effects reported from interventions (follow-up to 7 weeks postpartum).
Gocmen 1996[57]	Uterine curettage	No intervention					●		MAP control: improved in intervention group (difference not stated, p=0.01).
Gomez 2005[61]	Uterine curettage	No intervention					●	●	MAP control: improved in intervention group (difference not stated, p<0.001).<br>Safety: no significant difference. No complications reported from intervention.
Alkan 2006[62]	Uterine curettage	No intervention					●	●	MAP control: improved in intervention group (difference 6.8mmHg, p<0.05).<br>Safety: No significant difference. No complications reported from intervention.
Ragab 2013[15]	Uterine curettage	No intervention	●	●			●		Maternal mortality: no significant difference.<br>Maternal morbidity: no statistical analysis.<br>MAP control: improved in intervention group (difference at 6h postpartum 12.3mmHg, P=0.02, difference at 24h postpartum 9.2mmHg, p=0.01)
Mallapur 2015[18]	Uterine curettage	No intervention					●		MAP control: improved in intervention group (difference at 4h postpartum 7.6mmHg, p<0.001).
**ORGANISATION OF CARE (2 studies)**									
York 1997[26]	Nurse specialist follow-up	No intervention							N/A
Bibbo 2014[33]	Specialist postpartum clinic	No intervention							N/A

For peer review only

For peer review only - http://bmjopen.bmj.com/site/about/guidelines.xhtml

Legend for Tables 2a&b

• = improved in intervention group; • = no significant difference; • = improved in control group; • = unclear

For primary outcome assessment where there was a significant difference between groups, the magnitude of the difference is reported; where any adverse events or side effects were reported this is presented

For peer review only

For peer review only - http://bmjopen.bmj.com/site/about/guidelines.xhtml

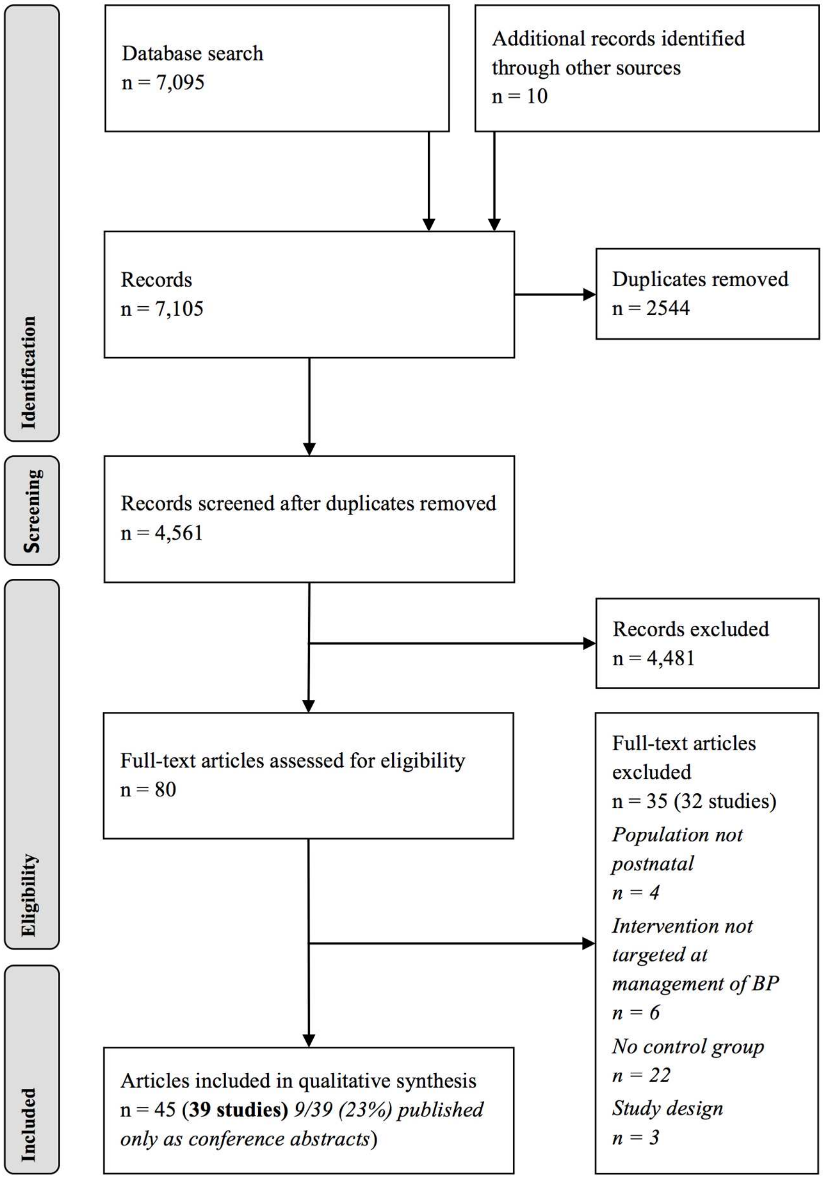

Figure 1: PRISMA Flowchart

137x193mm (300 x 300 DPI)

Appendix S1: Management of hypertensive disorders of pregnancy in the postpartum period: A systematic review protocol

Registration: PROSPERO CRD42015015527

http://www.crd.york.ac.uk/PROSPERO/display_record.asp?ID=CRD42015015527#.VL4ZI9KsWCk

Authors: Alexandra E Cairns, Louise Pealing, Nia Roberts, Richard J McManus
Corresponding author: Alexandra E Cairns, Nuffield Department of Primary Care Health Sciences, University of Oxford, New Radcliffe House, Radcliffe Observatory Quarter, Woodstock Road, Oxford, OX2 6GG
alexandra.cairns@phc.ox.ac.uk

Amendments: Protocol first published 22/12/2014 (version 1.0). Protocol amended (version 2.0 25/03/2015) to include all reporting items from the PRISMA-P 2015 checklist, and PROSPERO registration number.

Review funder: NIHR Collaborations for Leadership in Applied Health Research and Care (funding A Cairns' fellowship)

Review sponsor: University of Oxford

For peer review only

For peer review only - http://bmjopen.bmj.com/site/about/guidelines.xhtml

NUFFIELD DEPARTMENT OF PRIMARY CARE HEALTH SCIENCES
UNIVERSITY OF OXFORD

Abstract

Rationale: Hypertensive disorders of pregnancy (gestational hypertension and pre-eclampsia) are a leading cause of direct maternal death in the UK, and affect approximately 5-10% of pregnancies. Hypertensive disorders of pregnancy persist during the postpartum period, and complications can occur during this time.

Research question: How should hypertensive disorders of pregnancy be managed in the postnatal period to minimise harm to patients and optimise quality of life?

Objectives:

1. Organisation of care: how should blood pressure be monitored in women with hypertensive disorders of pregnancy in the postnatal period?
2. What blood pressure thresholds should be used for anti-hypertensive treatment initiation, adjustment and cessation in the postnatal period?
3. Which anti-hypertensive medication(s) should be used in the postnatal period?
4. What are the benefits and harms of other therapeutic interventions for women with hypertensive disorders of pregnancy in the postnatal period?

Search strategy: Medline and nine other electronic databases will be searched for articles published from inception until October 2014 using a search strategy designed to capture all the relevant literature concerning the management of hypertensive disorders of pregnancy in the postnatal period.

Study eligibility criteria:
Population: postnatal women with gestational hypertension or pre-eclampsia as defined by study
Intervention: therapeutic intervention for hypertensive disorders of pregnancy
Comparisons: another intervention, placebo or no intervention
Study design: RCT, prospective or retrospective cohort study or case-control study
Publication date: no restrictions
Language: no restrictions

Data management and extraction: Two reviewers will first review the titles of articles yielded by the search, and then the abstracts of articles of potential relevance. The full papers of potentially eligible papers will be assessed, and data extracted independently by the two reviewers using a data extraction sheet. Differences in study selection and data extraction will be resolved by discussion.

Assessment of methodological quality: This will be done using the Cochrane Collaboration's tool for assessing risk of bias in randomised trials, and for the assessment of bias in cohort and case-control studies we will use the Newcastle-Ottawa quality assessment scales.

Systematic review registration: This systematic review is registered with PROSPERO (International prospective register of systematic reviews).

For peer review only - http://bmjopen.bmj.com/site/about/guidelines.xhtml

NUFFIELD DEPARTMENT OF PRIMARY CARE HEALTH SCIENCES

Rationale

Definitions

The National Institute for Health and Clinical Excellence (NICE) defines gestational hypertension as new-onset raised blood pressure (≥ 140/90mmHg) beyond 20 weeks gestation. NICE defines pre-eclampsia as new-onset raised blood pressure (≥ 140/90mmHg) together with new-onset significant proteinuria (≥ 300mg/24hr), beyond 20 weeks gestation (1).

The International Society for the Study of Hypertension in Pregnancy (ISSHP) defines pre-eclampsia as new-onset raised blood pressure (as defined by NICE) in association with one of new-onset significant proteinuria (as defined by NICE), maternal organ dysfunction or uteroplacental insufficiency (2).

Epidemiology

Hypertensive disorders of pregnancy remain the second commonest direct cause of maternal death in the USA (3). Until recently this has also been the case in the UK (CMACE 2006-8)(4), but the most recent Confidential Enquiry into maternal deaths showed that for the triennium 2009-11, pre-eclampsia and eclampsia was the fourth commonest cause of direct death (behind thrombosis, genital tract sepsis and haemorrhage), with a rate of 0.42 deaths per 100,000 maternities (5).

A recent population-based retrospective study in the United States found the rate of pre-eclampsia to be 3.4%. This study showed a slight, but significant increase, in the rates of both mild, and to a greater extent, severe pre-eclampsia over the period studied (1980-2010) (6).

Reviews of the literature, and national guidelines, quote rates of gestational hypertension between 6% (7) and 15% (8). A retrospective study using data from the National Hospital Discharge Survey in the United States (1987-2004) demonstrated an incidence of 30.6 cases of gestational hypertension per 1000 deliveries in 2003-2004 (3.1%) (9). In a well-designed large randomised controlled trial assessing preventative strategies for hypertensive disorders of pregnancy in low risk, nulliparous women the incidence of gestational hypertension across both groups was 6% (10).

Physiology of blood pressure in pregnancy and postpartum

As a result of a significant decrease in systemic vascular resistance (as early as 5 weeks gestation) (11) there is a decrease in arterial pressures from early in the first trimester. Arterial pressures reach a nadir in the second trimester, and then begin to rise in the third trimester, before reaching near-preconception levels in the postnatal period (12).

For peer review only - http://bmjopen.bmj.com/site/about/guidelines.xhtml

NUFFIELD DEPARTMENT OF PRIMARY CARE HEALTH SCIENCES

Figure 1: Serial blood pressures before, during and after pregnancy (reproduced from the data of Mahendru et al. 2014) (12)

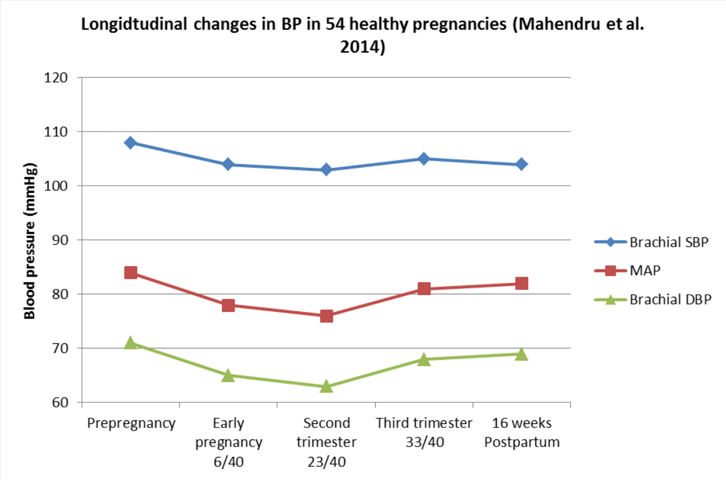

In gestational hypertension and pre-eclampsia the normal pregnancy-induced vasodilatation is reversed. In untreated women with pre-eclampsia significant increases in systemic vascular resistance are seen and result in elevation of blood pressure (13).

Hypertensive disorders of pregnancy in the postpartum period

There has been considerable focus on blood pressure control during pregnancy, especially with respect to pregnancy outcome. However, it is recognised that hypertensive disorders of pregnancy do persist during the postpartum period, and that complications can occur during this time. A small retrospective observational study published in 1987 looked at 67 women with moderate-severe pre-eclampsia: there was often an initial decrease in blood pressure after delivery, but this was followed by a rise to hypertensive levels in many women. In 50% of cases the blood pressure was 150/100mmHg or higher on day 5 after birth. The authors recommended continuing blood pressure monitoring and treatment in the postpartum period for women with a diagnosis of pre-eclampsia (14).

Most women with hypertensive disorders of pregnancy will be treatment-free by 3 months postpartum. In women whose blood pressure normalised after delivery the mean time to normalisation in a retrospective cohort study of 62 women was 5.4 weeks (15). This rapidly changing blood pressure, with shifting medication requirement, poses an additional challenge in terms of how best to manage this down-titration.

Approximately one third of eclamptic seizures occur postpartum, and studies suggest that over half of these seizures occur more than 48 hours after birth. Chames et al. (2002) highlight the importance of education of women and clinicians regarding prodromal symptoms of eclampsia in the postnatal

For peer review only - http://bmjopen.bmj.com/site/about/guidelines.xhtml

period (16). A case series published in 2005 of patients who sustained a stroke in association with severe pre-eclampsia or eclampsia, showed that more than half (57%) of these strokes occurred in the postpartum period (17).

Current guidelines

NICE guidelines highlight that very few clinical studies have addressed the management of blood pressure postpartum, and in practice clinical care is typically to continue antepartum antihypertensive medication and monitor blood pressure in the community with a focus on prevention of over-treatment.

NICE recommend frequency of monitoring in the postnatal period for both pre-eclampsia and gestational hypertension. The guidelines also stipulate thresholds for considering increasing or starting anti-hypertensive medication during this period (150/100 mmHg), and for reduction or stopping anti-hypertensive medication (consider at < 140/90 mmHg, and reduce at < 130/80 mmHg) (1).

Research question

How should hypertensive disorders of pregnancy be managed in the postnatal period to minimise harm to patients and optimise quality of life?

Objectives

The aim is to establish what evidence exists to guide the optimal approach to management of gestational hypertension and pre-eclampsia in the postnatal period. We want to address the specific sub-questions:

1. Organisation of care: how should blood pressure be monitored in women with hypertensive disorders of pregnancy in the postnatal period?
2. What blood pressure thresholds should be used for anti-hypertensive treatment initiation, adjustment and cessation in the postnatal period?
3. Which anti-hypertensive medication(s) should be used in the postnatal period?
4. What are the benefits and harms of other therapeutic interventions for women with hypertensive disorders of pregnancy in the postnatal period?

Information sources and search strategy

The systematic review of 'management of hypertensive disorders of pregnancy in the postpartum period' will be conducted in line with the PRISMA statement (18). Completion of a systematic review

For peer review only - http://bmjopen.bmj.com/site/about/guidelines.xhtml

NUFFIELD DEPARTMENT OF PRIMARY CARE HEALTH SCIENCES

is an iterative process, and it may be that modifications to the original review protocol are required during its conduct.

A search strategy designed to capture all the relevant literature concerning the management of hypertensive disorders of pregnancy in the postnatal period will be developed by an experienced trial search co-ordinator. Potentially relevant studies will be identified following screening of title and abstract of studies captured by the search and full text assessed for suitability.

Resources to be searched from inception to October 2014:

- Medline (Appendix 3) and 9 other electronic databases
- Trial registers (ClinicalTrials.gov; Current Controlled Trials; WHO; PROSPERO)
- Meta Search Engines
- Hand searches of reference lists
- Citation searching on Scopus and Web of Science
- Related articles search on PubMed
- Contact with authors and professional bodies / organisations: Experts in this field will be contacted for their recommendations of potentially relevant citations (19)

Study eligibility criteria

INCLUSION CRITERIA

Population: postnatal women with hypertensive disorders of pregnancy (gestational hypertension or pre-eclampsia).

Intervention: therapeutic intervention for management of hypertensive disorders of pregnancy

Comparisons: other intervention, placebo or no intervention

Study design: randomised controlled trial, cohort study (prospective and retrospective) or case-control study; human studies only

Publication Date: no restrictions

Language: no restrictions

EXCLUSION CRITERIA

Exclude report / study if **any** exclusion criteria fulfilled:

Population: antenatal or intrapartum women with hypertensive disorders of pregnancy; end-organ complications of pre-eclampsia (eclampsia, renal failure, HELLP syndrome)

For peer review only - http://bmjopen.bmj.com/site/about/guidelines.xhtml

Intervention: treatment of HELLP syndrome (haemolysis, elevated liver enzymes and low platelets); prevention or management of eclampsia; prevention of postpartum hypertension; choice of anaesthetic or sedative in pre-eclampsia; observational studies

Comparisons: no control group

Study design: guidelines, reviews, expert opinions, letters, commentaries, audits, case series and case reports excluded; animal studies

Data extraction

Two reviewers (AC and LP) will screen the titles and abstracts of articles yielded by the search against the eligibility criteria. Discrepancies will be resolved by consensus before determining the list of full papers for review. The reports will be screened independently by the two reviewers, and discrepancies will be resolved by discussion before deciding which papers to include in the review.

Data from included studies will be extracted independently by the two reviewers using a piloted and standardised data extraction sheet. Differences in data extraction will be resolved by discussion.

In the event that there is more than one report published about a single study: the reports will be reviewed separately but the data from that study grouped in our analysis, and the primary reference will be used.

In the event that data is missing from a report (for example the sole publication is a conference abstract) we will contact the authors directly to request further detail.

The study characteristics (study size, population, setting, study design, methodology, intervention, controls if applicable, outcome measures, and follow up period) will be recorded and reported.

Data synthesis

The data extracted will be aggregate.

Due to the heterogeneous nature of the outcomes reported in these studies a narrative synthesis is planned.

For trials where the population study is peripartum (i.e. a mixture of antepartum, intrapartum and postpartum) we will extract the data for the postpartum women and analyse this. If this is not feasible from the reported data then we will contact the study authors to request the data for this subgroup.

Outcomes

The results of all clinically relevant outcomes in hypertensive disorders of pregnancy that would be important to clinicians and patients will be extracted and reported.

The main outcomes we are interested in are listed in table 1 below:

For peer review only - http://bmjopen.bmj.com/site/about/guidelines.xhtml
For peer review only

NUFFIELD DEPARTMENT OF PRIMARY CARE HEALTH SCIENCES

Table 1

	Outcome measures	Timing
**Primary outcome(s)**	Maternal mortality<br>Major maternal morbidity (ischaemic stroke, intracranial haemorrhage, eclamptic seizure)<br>Systolic blood pressure control<br>Diastolic blood pressure control<br>Mean arterial pressure control	Direct maternal deaths upto day 42 postpartum; later maternal deaths upto 1 year postpartum
**Secondary outcome(s)**	Critical care admission<br>Postnatal readmission to secondary care<br>Length of hospital stay following delivery<br>Anti-hypertensive medication requirement<br>Maternal side effects of intervention<br>Development of pre-eclampsia with severe features<br>Postnatal complication requiring intervention<br>Urine output<br>Laboratory values	

Assessment of methodological quality

We will assess the risk of bias in each study. For randomised trials this will be done using the Cochrane Collaboration's tool for assessing risk of bias in randomised trials (Appendix 1, Table 2) (20). For each study the key domains will be identified, and then an overall assessment of bias within each trial made, according to the guidance published by the Cochrane Collaboration (Appendix 1, Table 2).

For the assessment of bias in cohort and case-control studies we will use the Newcastle-Ottawa quality assessment scales (Appendix 2, Tables 4 and 5) (21).

We will make a global assessment of bias across trials, based on the guidance from the Cochrane Collaboration (Appendix 1, Table 3):

- EITHER Most information is from trials at low risk of bias;
- OR most information is from trials at low or unclear risk of bias;
- OR the proportion of information from trials at high risk of bias is sufficient to affect the interpretation of results

For peer review only - http://bmjopen.bmj.com/site/about/guidelines.xhtml

Discussion

A Cochrane Review (2013) addresses the question of 'prevention and treatment of postpartum hypertension'. This only includes randomised controlled trials (9 in total), and does not address the issue of monitoring blood pressure during this period (22). Given the paucity of evidence cited in this area we believe there is a place for a review looking at all available evidence for the optimal approach to management of hypertensive disorders of pregnancy in the postpartum period.

Conflicts of interest

Neither AC nor LP have any conflicts of interest.

For peer review only

For peer review only - http://bmjopen.bmj.com/site/about/guidelines.xhtml

NUFFIELD DEPARTMENT OF PRIMARY CARE HEALTH SCIENCES
UNIVERSITY OF OXFORD

References

1. National Institute for Health and Care Excellence. NICE Clinical Guideline 107: Hypertension in pregnancy: the management of hypertensive disorders during pregnancy. 2011.
2. Tranquilli AL, Dekker G, Magee L, Roberts J, Sibai BM, Steyn W, et al. The classification, diagnosis and management of the hypertensive disorders of pregnancy: A revised statement from the ISSHP. Pregnancy Hypertension: An International Journal of Women's Cardiovascular Health. 2014;4(2):97-104.
3. American College of Obstetricians and Gynecologists. Diagnosis and management of preeclampsia and eclampsia. ACOG Practice Bulletin. ACOG Practice Bulletin No. 33: 2002.
4. Cantwell R, Clutton-Brock T, Cooper G, Dawson A, Drife J, Garrod D, et al. Saving Mothers' Lives: Reviewing maternal deaths to make motherhood safer: 2006-2008. The Eighth Report of the Confidential Enquiries into Maternal Deaths in the United Kingdom. BJOG : an international journal of obstetrics and gynaecology. 2011;118 Suppl 1:1-203.
5. Knight M, Kenyon S, Brocklehurst P, Neilson J, Shakespeare J, Kurinczuk J. Saving Lives, Improving Mothers' Care (MBRRACE-UK). 2014.
6. Ananth CV, Keyes KM, Wapner RJ. Pre-eclampsia rates in the United States, 1980-2010: age-period-cohort analysis. Bmj. 2013;347:f6564.
7. Yoder SR, Thornburg LL, Bisognano JD. Hypertension in pregnancy and women of childbearing age. The American journal of medicine. 2009;122(10):890-5.
8. National Institute for Health and Care Excellence. NICE Clinical Guideline 107: Hypertension in pregnancy: Costing report. Implementing NICE guidance. 2010.
9. Wallis AB, Saftlas AF, Hsia J, Atrash HK. Secular trends in the rates of preeclampsia, eclampsia, and gestational hypertension, United States, 1987-2004. American journal of hypertension. 2008;21(5):521-6.
10. Sibai BM, Caritis SN, Thom E, Klebanoff M, McNellis D, Rocco L, et al. Prevention of preeclampsia with low-dose aspirin in healthy, nulliparous pregnant women. The National Institute of Child Health and Human Development Network of Maternal-Fetal Medicine Units. The New England journal of medicine. 1993;329(17):1213-8.
11. Chapman AB, Abraham WT, Zamudio S, Coffin C, Merouani A, Young D, et al. Temporal relationships between hormonal and hemodynamic changes in early human pregnancy. Kidney international. 1998;54(6):2056-63.
12. Mahendru AA, Everett TR, Wilkinson IB, Lees CC, McEniery CM. A longitudinal study of maternal cardiovascular function from preconception to the postpartum period. Journal of hypertension. 2014;32(4):849-56.
13. Visser W, Wallenburg HC. Central hemodynamic observations in untreated preeclamptic patients. Hypertension. 1991;17(6 Pt 2):1072-7.
14. Walters BN, Walters T. Hypertension in the puerperium. Lancet. 1987;2(8554):330.
15. Podymow T, August P. Postpartum course of gestational hypertension and preeclampsia. Hypertension in pregnancy. 2010;29(3):294-300.
16. Chames MC, Livingston JC, Ivester TS, Barton JR, Sibai BM. Late postpartum eclampsia: a preventable disease? American journal of obstetrics and gynecology. 2002;186(6):1174-7.
17. Martin JN, Jr., Thigpen BD, Moore RC, Rose CH, Cushman J, May W. Stroke and severe preeclampsia and eclampsia: a paradigm shift focusing on systolic blood pressure. Obstetrics and gynecology. 2005;105(2):246-54.
18. Moher D, Liberati A, Tetzlaff J, Altman DG, Group P. Preferred reporting items for systematic reviews and meta-analyses: the PRISMA statement. PLoS medicine. 2009;6(7):e1000097.
19. McManus RJ, Wilson S, Delaney BC, Fitzmaurice DA, Hyde CJ, Tobias RS, et al. Review of the usefulness of contacting other experts when conducting a literature search for systematic reviews. Bmj. 1998;317(7172):1562-3.

For peer review only - http://bmjopen.bmj.com/site/about/guidelines.xhtml

20. Higgins JPT, Altman DG, Gøtzsche PC, Jüni P, Moher D, Oxman AD, et al. The Cochrane Collaboration's tool for assessing risk of bias in randomised trials2011 2011-10-18 10:55:48.
21. Wells GA, Shea B, O'Connell D, J. P, V. W, M. L, et al. The Newcastle-Ottawa Scale (NOS) for assessing the quality of nonrandomised studies in meta-analyses.
22. Magee L, von Dadelszen P. Prevention and treatment of postpartum hypertension. The Cochrane database of systematic reviews. 2013;4:CD004351.

For peer review only

For peer review only - http://bmjopen.bmj.com/site/about/guidelines.xhtml

NUFFIELD DEPARTMENT OF PRIMARY CARE HEALTH SCIENCES

Appendix 1

Table 2: Cochrane Collaboration's tool for assessing risk of bias (*adapted from Higgins and Altman*)(20)

**Bias domain**	**Source of bias**	**Support for judgment**	**Review authors' judgment** (assess as low, unclear or high risk of bias)
**Selection bias**	Random sequence generation	Describe the method used to generate the allocation sequence in sufficient detail to allow an assessment of whether it should produce comparable groups	Selection bias (biased allocation to interventions) due to inadequate generation of a randomised sequence
	Allocation concealment	Describe the method used to conceal the allocation sequence in sufficient detail to determine whether intervention allocations could have been foreseen before or during enrolment	Selection bias (biased allocation to interventions) due to inadequate concealment of allocations before assignment
**Performance bias**	Blinding of participants and personnel*	Describe all measures used, if any, to blind trial participants and researchers from knowledge of which intervention a participant received. Provide any information relating to whether the intended blinding was effective	Performance bias due to knowledge of the allocated interventions by participants and personnel during the study
**Detection bias**	Blinding of outcome assessment*	Describe all measures used, if any, to blind outcome assessment from knowledge of which intervention a participant received. Provide any information relating to whether the intended blinding was effective	Detection bias due to knowledge of the allocated interventions by outcome assessment
**Attrition bias**	Incomplete outcome data*	Describe the completeness of outcome data for each main outcome, including attrition and exclusions from the analysis. State whether attrition and exclusions were reported, the numbers in each intervention group (compared with total randomised participants), reasons for attrition or exclusions where reported, and any re-inclusions in analyses for the review	Attrition bias due to amount, nature, or handling of incomplete outcome data
**Reporting bias**	Selective reporting	State how selective outcome reporting was examined and what	Reporting bias due to selective

For peer review only - http://bmjopen.bmj.com/site/about/guidelines.xhtml

		was found	outcome reporting
**Other bias**	Anything else, ideally Pre-specified	State any important concerns about bias not covered in the other domains in the tool	Bias due to problems not covered elsewhere

*Assessments should be made for each main outcome or class of outcomes.

Table 3: Approach to formulating summary assessments of risk of bias for each important outcome (across domains) within and across trials (*adapted from Higgins and Altman*)(20)

Risk of bias	Interpretation	Within a trial	Across trials
**Low risk of bias**	Bias, if present, is unlikely to alter the results seriously	Low risk of bias for all key domains	Most information is from trials at low risk of bias
**Unclear risk of bias**	A risk of bias that raises some doubt about the results	Low or unclear risk of bias for all key domains	Most information is from trials at low or unclear risk of bias
**High risk of bias**	Bias may alter the results seriously	High risk of bias for one or more key domains	The proportion of information from trials at high risk of bias is sufficient to affect the interpretation of results

For peer review only

For peer review only - http://bmjopen.bmj.com/site/about/guidelines.xhtml

NUFFIELD DEPARTMENT OF PRIMARY CARE HEALTH SCIENCES

UNIVERSITY OF OXFORD

Appendix 2

Table 4: Newcastle-Ottawa quality assessment scale case control studies(21)

A study can be awarded a maximum of one star for each numbered item within the Selection and Exposure categories. A maximum of two stars can be given for Comparability.

**Selection**	Is the case definition adequate?	a) Yes, with independent validation ✵<br>b) Yes, e.g. record linkage or based on self-reports<br>c) No description
	Representativeness of the cases	a) Consecutive or obviously representative series of cases ✵<br>b) Potential for selection biases not stated
	Selection of controls	a) Community controls ✵<br>b) Hospital controls<br>c) No description
	Definition of controls	a) No history of disease (endpoint) ✵<br>b) No description of source
**Comparability**	Comparability of cases and controls on the basis of the design or analysis	a) Study controls for <<_>> (select the post important factor) ✵<br>b) Study controls for any additional factor ✵
**Exposure**	Ascertainment of exposure	a) Secure records (e.g. surgical records) ✵<br>b) Structured interview where blind to case/control status ✵<br>c) Interview not blinded to case/control status<br>d) Written self-report or medical record only<br>e) No description
	Same method of ascertainment for cases and controls	a) Yes ✵<br>b) No
	Non-response rate	a) Same rate for both groups ✵<br>b) Non-respondents described<br>c) Rate different and no designation

Table 5: Newcastle-Ottawa quality assessment scale cohort studies(21)

A study can be awarded a maximum of one star for each numbered item within the Selection and Outcome categories. A maximum of two stars can be given for Comparability.

**Selection**	Representativeness of the exposed cohort	a) Truly representative of the average <<_>> (describe) in the community ✵<br>b) Somewhat representative of the average <<_>> (describe) in the community ⍰<br>c) Selected group of users e.g. nurses, volunteers<br>d) No description of the derivation of the cohort
	Selection of the non-exposed cohort	a) Drawn from the same community as the exposed cohort ✵<br>b) Drawn from a different source<br>c) No description of the derivation of the non-exposed cohort
	Ascertainment of exposure	a) Secure record (e.g. surgical records) ✵<br>b) Structured interview ✵<br>c) Written self-report<br>d) No description

For peer review only

For peer review only - http://bmjopen.bmj.com/site/about/guidelines.xhtml

NUFFIELD DEPARTMENT OF PRIMARY CARE HEALTH SCIENCES

	Demonstration that the outcome of interest was not present at start of study	a) Yes ✸<br>b) No
**Comparability**	Comparability of cases and controls on the basis of the design or analysis	a) Study controls for <<_>> (select the post important factor) ✸<br>b) Study controls for any additional factor ✸
**Outcome**	Assessment of outcome	a) Independent blind assessment ✸<br>b) Record linkage ✸<br>c) Self-report<br>d) No description
	Was follow-up long enough for outcomes to occur	a) Yes (select an adequate follow up period for outcome of interest) ✸<br>b) No
	Adequacy of follow-up of cohorts	a) Complete follow-up – all subjects accounted for ✸<br>b) Subjects lost to follow-up unlikely to introduce bias: >_ _ % (select an adequate %) follow-up rate, or description provided of those lost) ✸<br>c) Follow-up rate < _ _ % (select an adequate %) and no description of those lost<br>d) No statement

For peer review only

For peer review only - http://bmjopen.bmj.com/site/about/guidelines.xhtml

Appendix 3: Medline search strategy

# ▼	Searches	Results
1	Pregnancy/ and Hypertension/	9226
2	exp Hypertension, Pregnancy-Induced/	29022
3	((pregnan* or gestation* or maternal or prenatal or pre-natal or antenatal or ante-natal or antepart* or ante-part* or obstetric*) and (hypertens* or blood pressure or bp or dbp or sbp or diastolic or systolic)).ti.	6787
4	((pregnan* or gestation* or maternal or prenatal or pre-natal or antenatal or ante-natal or antepart* or ante-part* or obstetric*) adj3 (hypertens* or blood pressure or bp or dbp or sbp or diastolic or systolic)).ti,ab.	12434
5	(eclamp* or preeclamp* or pre-eclamp* or hellp).ti,ab.	25194
6	1 or 2 or 3 or 4 or 5	46611
7	Postnatal Care/	4044
8	Aftercare/	6684
9	Postpartum Period/ and Maternal Health Services/	126
10	exp Puerperal Disorders/ and Maternal Health Services/	196
11	Postpartum period/ and (exp Antihypertensive agents/ or exp calcium channel blockers/ or exp Angiotensin-Converting Enzyme Inhibitors/ or exp Adrenergic beta-Antagonists/ or exp Diuretics/)	187
12	exp Puerperal disorders/ and (exp Antihypertensive agents/ or exp calcium channel blockers/ or exp Angiotensin-Converting Enzyme Inhibitors/ or exp Adrenergic beta-Antagonists/ or exp Diuretics/)	237
13	Postpartum period/ and exp Curettage/	30
14	exp Puerperal disorders/ and exp Curettage/	118
15	Postpartum period/ and hypertension/dt, th	33
16	exp Puerperal disorders/ and hypertension/dt, th	54
17	exp Puerperal disorders/dt, th	6408
18	((postnatal or post-natal or postpart* or post-part* or puerper*) adj5 (care or healthcare or service* or program* or scheme* or intervention*)).ti,ab.	4407
19	((postnatal or post-natal or postpart* or post-part* or puerper*) adj5 (clinic? or unit? or visit* or referral? or appointment?)).ti,ab.	1491
20	((postnatal or post-natal or postpart* or post-part* or puerper*) adj5 (manage* or treat* or therap* or medication? or recovery)).ti,ab.	7287
21	((postnatal or post-natal or postpart* or post-part* or puerper*) adj5 (antihypertens* or anti-hypertens* or calcium channel block* or beta block* or b block* or ace inhibitor* or angiotensin converting enzyme inhibitor* or diuretic*)).ti,ab.	41
22	((postnatal or post-natal or postpart* or post-part* or puerper*) adj5 (evaluat* or assess* or screen* or diagnos* or monitor* or follow up or supervis*)).ti,ab.	7562
23	((postnatal or post-natal or postpart* or post-part* or puerper*) adj5 curet*).ti,ab.	82
24	(postnatal or post-natal or postpart* or post-part* or puerper*).ti.	41491
25	7 or 8 or 9 or 10 or 11 or 12 or 13 or 14 or 15 or 16 or 17 or 18 or 19 or 20 or 21 or 22 or 23 or 24	64775

For peer review only - http://bmjopen.bmj.com/site/about/guidelines.xhtml

NUFFIELD DEPARTMENT OF PRIMARY CARE HEALTH SCIENCES

26	6 and 25	1896
27	((postnatal or post-natal or postpart* or post-part* or puerper*) and (hypertens* or blood pressure)).ti.	270
28	26 or 27	1990
29	exp animals/ not humans.sh.	4079856
30	(rat or rats or rodent? or mice or mouse or cow or cows or cattle or calf or calves or ewe? or sheep or goat or ruminant? or pig or pigs or minipig? or chicken? or horse or horses or murine or bovine or ovine or porcine or animal?).ti.	1682619
31	29 or 30	4373527
32	28 not 31	1881

For peer review only

For peer review only - http://bmjopen.bmj.com/site/about/guidelines.xhtml

Appendix S2: PRISMA 2009 Checklist

Section/topic	#	Checklist item	Reported on page #
**TITLE**			
Title	1	Identify the report as a systematic review, meta-analysis, or both.	1
**ABSTRACT**			
Structured summary	2	Provide a structured summary including, as applicable: background; objectives; data sources; study eligibility criteria, participants, and interventions; study appraisal and synthesis methods; results; limitations; conclusions and implications of key findings; systematic review registration number.	2
**INTRODUCTION**			
Rationale	3	Describe the rationale for the review in the context of what is already known.	5
Objectives	4	Provide an explicit statement of questions being addressed with reference to participants, interventions, comparisons, outcomes, and study design (PICOS).	5-6
**METHODS**			
Protocol and registration	5	Indicate if a review protocol exists, if and where it can be accessed (e.g., Web address), and, if available, provide registration information including registration number.	6
Eligibility criteria	6	Specify study characteristics (e.g., PICOS, length of follow-up) and report characteristics (e.g., years considered, language, publication status) used as criteria for eligibility, giving rationale.	6
Information sources	7	Describe all information sources (e.g., databases with dates of coverage, contact with study authors to identify additional studies) in the search and date last searched.	6
Search	8	Present full electronic search strategy for at least one database, including any limits used, such that it could be repeated.	Appendix S1
Study selection	9	State the process for selecting studies (i.e., screening, eligibility, included in systematic review, and, if applicable, included in the meta-analysis).	6
Data collection process	10	Describe method of data extraction from reports (e.g., piloted forms, independently, in duplicate) and any processes for obtaining and confirming data from investigators.	6-7
Data items	11	List and define all variables for which data were sought (e.g., PICOS, funding sources) and any assumptions and simplifications made.	6-7; Table 1
Risk of bias in individual studies	12	Describe methods used for assessing risk of bias of individual studies (including specification of whether this was done at the study or outcome level), and how this information is to be used in any data synthesis.	7
Summary measures	13	State the principal summary measures (e.g., risk ratio, difference in means).	6-7 (narrative)
Synthesis of results	14	Describe the methods of handling data and combining results of studies, if done, including measures of consistency (e.g., I^2) for each meta-analysis.	N/A

From: Moher D, Liberati A, Tetzlaff J, Altman DG, The PRISMA Group (2009). Preferred Reporting Items for Systematic Reviews and Meta-Analyses: The PRISMA Statement. PLoS Med 6(7): e1000097. doi:10.1371/journal.pmed1000097. For more information, visit: www.prisma-statement.org.

For peer review only

Appendix S2: PRISMA 2009 Checklist

Section/topic	#	Checklist item	Reported on page #
Risk of bias across studies	15	Specify any assessment of risk of bias that may affect the cumulative evidence (e.g., publication bias, selective reporting within studies).	7
Additional analyses	16	Describe methods of additional analyses (e.g., sensitivity or subgroup analyses, meta-regression), if done, indicating which were pre-specified.	N/A
**RESULTS**			
Study selection	17	Give numbers of studies screened, assessed for eligibility, and included in the review, with reasons for exclusions at each stage, ideally with a flow diagram.	7; Figure 1
Study characteristics	18	For each study, present characteristics for which data were extracted (e.g., study size, PICOS, follow-up period) and provide the citations.	7; Appendix S4
Risk of bias within studies	19	Present data on risk of bias of each study and, if available, any outcome level assessment (see item 12).	7-8; Appendix S6
Results of individual studies	20	For all outcomes considered (benefits or harms), present, for each study: (a) simple summary data for each intervention group (b) effect estimates and confidence intervals, ideally with a forest plot.	8-13; Tables 2a+b; Appendix S5
Synthesis of results	21	Present results of each meta-analysis done, including confidence intervals and measures of consistency.	N/A
Risk of bias across studies	22	Present results of any assessment of risk of bias across studies (see Item 15).	7-8; Appendix S6
Additional analysis	23	Give results of additional analyses, if done (e.g., sensitivity or subgroup analyses, meta-regression [see Item 16]).	N/A
**DISCUSSION**			
Summary of evidence	24	Summarize the main findings including the strength of evidence for each main outcome; consider their relevance to key groups (e.g., healthcare providers, users, and policy makers).	13-16
Limitations	25	Discuss limitations at study and outcome level (e.g., risk of bias), and at review-level (e.g., incomplete retrieval of identified research, reporting bias).	14-15
Conclusions	26	Provide a general interpretation of the results in the context of other evidence, and implications for future research.	15-16
**FUNDING**			
Funding	27	Describe sources of funding for the systematic review and other support (e.g., supply of data); role of funders for the systematic review.	17

For peer review only - http://bmjopen.bmj.com/site/about/guidelines.xhtml

From: Moher D, Liberati A, Tetzlaff J, Altman DG, The PRISMA Group (2009). Preferred Reporting Items for Systematic Reviews and Meta-Analyses: The PRISMA Statement. PLoS Med 6(7): e1000097. doi:10.1371/journal.pmed1000097. For more information, visit: www.prisma-statement.org.

Appendix S3: Primary reasons for article exclusion (n = 35)

	Population not postnatal	Intervention not targeted at management of BP	No control group	Study design
**n**	4	6	22	3
**Study IDs**	Berks 2015<br>Gerard 1983<br>Scardo 1999<br>Wacker 2006	Chandrasekaran 2015<br>Ehrenberg 2004<br>Ehrenberg 2006<br>Ossada 2016<br>Wasden 2012<br>Younger-Lewis 2016	Al Waili 2004<br>Alicino 1962<br>Barton 1991<br>Belfort 1988<br>Belfort 1992<br>Bittle 2014<br>Bosio 2003<br>Correa 1982<br>Dulitzky 1987<br>Hirshberg 2016<br>Hirshberg 2017<br>Hunter 1961<br>Onishi 2015<br>Robinson 1964<br>Rodriguez 2012<br>Saghir<br>Smith 2005<br>Sukerman-Voldman 1985<br>Taslimi 1991<br>Tkacheva 2006<br>Wacker 1994<br>Walters 1984	Editor, Emergency Medicine 1990<br>Cursino 2015<br>Gallegos 1961

For peer review only

APPENDIX S4: Main characteristics of included studies (n=39)

Author and year	Methods		Participants				Intervention		Outcomes	
	Study design	*Duration*	*n*[*]	*Age /y*[†]	*Setting*	*Country*	*Intervention*	*Control(s)*	*Primary*	*Secondary*
**ANTIHYPERTENSIVE MEDICATIONS (18 studies)**										
**Calcium channel blockers**										
**Barton 1990**[32]	RCT	Enrolled immediately after birth<br>F/U 48h	31	24.0<br>26.3	Tertiary referral hospital	USA	Nifedipine 10mg PO 4-hourly for 48 hours	Placebo	MAP	SBP, DBP<br>Maternal heart rate<br>AHT requirement<br>Urine output<br>Laboratory values (urine protein, creatinine clearance, HCT, plt, ALT, BUN, creatinine, serum electrolytes, UA, urine specific gravity)
Vermillion 1999[21]	RCT	Enrolled within 24h of birth<br>F/U 3 – 24h	21	27.2±7.3<br>27.0±6.4	Tertiary referral hospital	USA	Nifedipine 10mg stat PO then 20mg every 20min until BP <160/110mmHg or max 5 doses + IV placebo	Labetalol 20mg, then 40mg, then 80mg IV every 20min until BP <160/110mmHg or max 5 doses (300mg) + PO placebo	SBP + DBP	SBP (failure to achieve target <160mmHg)<br>DBP (failure to achieve target <110mmHg)<br>Maternal side effects<br>AHT requirement<br>Urine output
**Sayin 2005**[34]	RCT	Enrolled 24h after birth<br>F/U 72h after BP controlled	83	17-41	Tertiary referral hospital	Turkey[‡]	Nifedipine 10mg PO QDS until BP <150/100mmHg for 48h	Methyldopa 250mg PO TDS	SBP + DBP	Maternal mortality<br>AHT requirement<br>Hypertensive retinopathy
**Vasodilators**										
Palot 1979[36]	Retrospective cohort study	Not specified	54	24.5 (17-37)	Not specified	France[†]	Hydralazine 5mg IV stat then 1% IV infusion, furosemide 20mg IV stat and 30% hypertonic glucose	(1) Clonidine IV and furosemide 20mg IV stat *Or* (2) Non-systematic treatment	Maternal morbidity (development of pre-eclampsia with severe features)	BP (time to resolution of hypertension)

Abbreviations: AHT = antihypertensive; ALT = alanine aminotransferase; BD = twice daily; BP = blood pressure; BUN = blood urea nitrogen; DBP = diastolic blood pressure; F/U = follow-up; IM = intramuscular; IV = intravenous; MAP = mean arterial pressure; plt = platelets; PN = postnatal; OD = once daily; PO = oral; QDS = four times daily; RCT = randomised controlled trial; SBP = systolic blood pressure; S/L = sublingual; TDS = three times daily; UA = uric acid

[*] n = postnatal population (antenatal excluded)

[†] Where separate data available for study groups, intervention group listed first

[‡] Non-English language manuscript

For peer review only - http://bmjopen.bmj.com/site/about/guidelines.xhtml

For peer review only

Author and year	Methods		Participants				Intervention		Outcomes	
	Study design	*Duration*	*n*[*]	*Age /y*[†]	*Setting*	*Country*	*Intervention*	*Control(s)*	*Primary*	*Secondary*
**Griffis 1989**[38 39]	RCT	F/U 24h	26	Not specified	Tertiary referral hospital	USA	Hydralazine 20mg IM QDS for 24h	Methyldopa 250mg IV QDS x 24h	MAP	Maternal side effects<br>AHT requirement<br>Urine output (time to diuresis)
**Walss Rodriguez 1991**[40]	RCT	Not specified	38	16-40	Not specified	Mexico[†]	Hydralazine 40mg PO QDS, duration not specified; if DBP >110mmHg PRN nifedipine 10mg SL every 30min, to max 3 doses	Nifedipine 10mg SL every 30min if DBP ≥110mmHg	SBP	DBP<br>AHT requirement
Begum 2002[17]	Quasi-randomised trial	Not specified	15	24.09±4.93<br>22.72±5.08	Tertiary referral hospital	Bangladesh	Hydralazine 5mg then 2mg IV bolus every 15min until DBP 90-95mmHg	Hydralazine 20mg/200ml normal saline IV infusion; 10 drops per min, increased by 5 drops at 15min intervals; until DBP 90-95mmHg	DBP	Maternal side effects<br>AHT requirement<br>Maternal heart rate
**Vigil-De Gracia 2007**[35]	RCT	Enrolled day 2-3 after birth<br>F/U not specified	82	29.9±5.9<br>31.3±5.5	Tertiary referral hospital	Panama	Hydralazine 5mg IV every 20min until BP <160/110mmHg or max 5 doses	Labetalol 20mg, then 40mg, then 80mg IV every 20min until BP <160/110mmHg or max 5 doses (300mg)	SBP + DBP	Maternal mortality<br>Maternal morbidity (development of pre-eclampsia with severe features)<br>Maternal side effects<br>AHT requirement<br>Maternal heart rate
Hennessy 2007[23]	RCT	F/U 3h	37	21-43<br>(mean 33)	Tertiary referral hospital	Australia	Diazoxide 15mg IV every min, maximum dose 300mg	Hydralazine 5mg IV every 2min, maximum 15mg	SBP + DBP	SBP (10mmHg above target after 1 hour)<br>DBP (10mmHg above target after 1 hour)<br>Maternal side effects (including hypotension)<br>Time taken to administer drug
**Beta blockers**										
Garden 1982[24]	RCT	Enrolled immediately after birth<br>F/U 45-64h	6	25-44<br>20-28	Tertiary referral hospital	South Africa	Labetalol 200mg/200ml 5% dextrose, 20mg/h IV infusion, doubled every 30min until DBP <100mmHg or maximum dose 160mg/h	Dihydralazine 100mg/200ml 5% dextrose, 10mg/h IV infusion, doubled every 30min until DBP <100mmHg or maximum dose 80mg/h	DBP	Maternal side effects
**Fidler 1982**[42]	RCT	Enrolled 4 days after birth<br>F/U 9 days	80	29.7±1.0<br>27.8±0.9	Tertiary referral hospital	UK	Timolol 5mg PO TDS for 9 days	Methyldopa 250mg PO TDS for 9 days	DBP	SBP<br>DBP (time to achieve control, proportion achieving control)<br>Maternal side effects

For peer review only

Author and year	Methods		Participants				Intervention		Outcomes	
	Study design	*Duration*	*n*[*]	*Age /y*[†]	*Setting*	*Country*	*Intervention*	*Control(s)*	*Primary*	*Secondary*
Mabie 1987[22]	RCT	Enrolled 1-96 hours after birth<br>F/U 3h	41	23.7±6.9<br>22.9±7.0	Tertiary referral hospital	USA	Labetalol 20mg IV every 10min then escalating until DBP <100mmHg or maximum cumulative dose reached (300mg)	Hydralazine 5mg IV every 10minuntil DBP <100mmHg	MAP	MAP (time to maximal decrease)<br>DBP (achieving target <100mmHg)<br>Maternal side effects<br>AHT requirement<br>Maternal heart rate
Shumard 2016[41]	Retrospective cohort study	F/U not specified (but >24h)	128	Not specified	Not specified	USA	Labetalol PO (variable dose and frequency)	Nifedipine PO (variable dose and frequency)	Length of hospital stay after birth	SBP, DBP<br>AHT requirement
Sharma 2017[27,28]	RCT	F/U not specified (but >24h)	50	Not specified	Tertiary referral hospital	USA	Labetalol 200mg PO BD	Nifedipine XL 30mg PO OD	SBP + DBP	Maternal side-effects<br>Length of PN hospital stay<br>AHT requirement
**Other**										
Gaisin 2013[25]	RCT	6 months	30	23-29	Not specified (hospital)	Russia	Indapamide 1.5mg PO OD, duration unclear	Adjusted dose methyldopa	SBP + DBP	Safety data<br>Laboratory values (lipid and glucose metabolism)<br>Adherence to treatment<br>Weight reduction<br>Decrease in albuminuria<br>Decrease in LV mass index<br>Endothelial function<br>Milk production
Gaisin 2014[37]	RCT	1 year	30	24-28	Not specified (hospital)	Russia	Indapamide 1.5mg PO OD with ursodeoxycholic acid 250mg PO TDS, duration unclear	Adjusted dose methyldopa	SBP + DBP	Maternal side effects<br>Laboratory values (atherogenic lipid profile, glucose metabolism. renal function)<br>Offspring adverse events<br>Weight reduction<br>Decrease in microalbuminuria<br>Decrease in LV mass index<br>Endothelial function
Krebs 1956[43,44]	Retrospective cohort study	F/U not specified (but >24h)	140	Not specified	Not specified	Switzer-land[†]	Reserpine 0.25mg PO or IM TDS or QDS for 7 days	Phenobarbital	SBP + DBP	SBP + DBP (non-responders)<br>Maternal side effects<br>Resolution of albuminuria<br>Resolution of oedema
Katz 2015[29-31]	RCT	F/U not specified	90	Not specified	Tertiary referral hospital	Brazil	Clonidine 0.1mg PO repeated every 20min to maximum 6 doses	Captopril 25mg PO repeated every 20min to maximum 6 doses	SBP + DBP	SBP (% reduction)<br>SBP + DBP (daily mean)<br>Maternal side effects<br>AHT requirement
**LOOP DIURETICS (3 studies)**										
**Matthews 1997[46]**	RCT	Enrolled 12-24h after birth<br>F/U 6 weeks	19	Not specified	Tertiary referral hospital	UK	Furosemide 40mg PO OD for 7 days	Placebo	MAP	Length of PN hospital stay<br>AHT requirement<br>Urine output<br>Laboratory values (hypokalemia)

Author and year	Methods		Participants				Intervention		Outcomes	
	Study design	*Duration*	*n*[*]	*Age /y*[†]	*Setting*	*Country*	*Intervention*	*Control(s)*	*Primary*	*Secondary*
**Ascarelli 2005**[16]	RCT	Enrolled 2-24h after birth<br>F/U 6 weeks	264	22.8±6.1<br>22.9±6.0	Tertiary referral hospital	USA	Furosemide 20mg PO OD + potassium 20mEq PO OD for 5 days	No intervention	SBP	Maternal morbidity (postnatal complication requiring intervention)<br>DBP<br>Length of PN hospital stay<br>AHT requirement<br>Maternal weight<br>Maternal HR<br>Duration of magnesium sulphate
Amorim 2015[45]	RCT	Enrolled immediately after birth<br>F/U 5 days	120	Not specified	Tertiary referral hospital	Brazil	Furosemide 40mg PO OD, duration not specified	Placebo	SBP + DBP	MAP<br>SBP (daily episodes ≥180mmHg)<br>DBP (daily episodes ≥110mmHg)<br>Length of PN hospital stay<br>AHT requirement<br>Urine output<br>Maternal heart rate
Veena 2017[19]	RCT	Enrolled <24h after birth	100	24.34±4.31<br>24.02±4.27	Tertiary referral hospital	India	Furosemide 20mg PO OD + nifedipine 10mg PO TDS for 3 days	Nifedipine 10mg PO TDS for 3 days	SBP + DBP	MAP<br>Maternal morbidity (postnatal complication requiring intervention)<br>Length of hospital stay after birth<br>Antihypertensive medication requirement<br>Urine output
**OTHER DRUGS (7 studies)**										
**Selective 5-HT antagonists**										
Weiner 1982[48]	RCT (crossover)	F/U not specified	5	Not specified	Tertiary referral	USA	R41468 IV (dose not specified) bolus then infusion for 90min	Placebo	MAP	MAP (rate at which hypertension returned post-infusion)<br>Urine output (infusion related diuresis)
Weiner 1984[49]	RCT (crossover)	Enrolled immediately after birth<br>F/U 3.5h	20	28±6.4	Tertiary referral	USA	Ketanserin 10mg IV bolus then 4mg/hr IV infusion. Repeat bolus after 5min if no response.	Placebo	SBP + DBP	Maternal side effects<br>DBP (target <95mmHg)<br>MAP<br>AHT requirement<br>Response rate
Montenegro 1985[50]	RCT (crossover)	Enrolled immediately after birth<br>F/U not specified	30	21.5 (13-31)	Tertiary referral hospital	USA	Ketanserin 10mg IV bolus, repeated if no response. If no response to second bolus IV infusion 4mg/hr (increments of 2mg/hr every 10min to max 12mg/hr).	Placebo	MAP	Maternal side effects

For peer review only

Author and year	Methods		Participants				Intervention		Outcomes	
	Study design	*Duration*	*n**	*Age /y*†	*Setting*	*Country*	*Intervention*	*Control(s)*	*Primary*	*Secondary*
**Alternative therapies**										
**Hladunewich 2006**[51]	RCT	Enrolled immediately after birth<br>F/U 10 days	45	29±6<br>28±7	Tertiary referral hospital	USA	L-arginine 3.5g PO QDS or 10g IV TDS for 3-9 days	Placebo	MAP	Maternal side effects<br>SBP, DBP<br>AHT requirement<br>Laboratory values (glomerular filtration rate (GFR) (inulin clearance), Albumin/creatinine (A/C) ratio, vasoactive hormones (NO and cGMP), liver function tests (LFTs), plt)<br>Renal plasma flow (para-amino hippurate clearance), renal blood flow = renal plasma flow / (1-HCT), renovascular resistance
Liu 2009[52]	Quasi-randomised trial	Enrolled day 2 after birth<br>F/U 3 weeks	72	26.6±3.7<br>25.7±3.9	District general hospital	China†	Shengkangbao 10g PO or IV BD for 3 weeks	No intervention	Percentage of cases with positive albuminuria	SBP, DBP<br>Laboratory values (24h urinary albumin, plasma total protein, plasma albumin, urinary albumin negative inversion rate, renal function)
**Steroids**										
Barrilleaux 2005[53 54]	RCT	Enrolled immediately after birth<br>F/U 4.5 days	157 (175)	24.5±6.8<br>23.9±6.4	Tertiary referral hospital	USA	Dexamethasone 10mg x 2, then 5mg x 2 IV BD for 48 hours	Placebo	Antihypertensive medication requirement	MAP<br>Critical care admission<br>Length of PN hospital stay<br>Urine output<br>Laboratory values (plt, lactate dehydrogenase (LDH), aspartate aminotransferase (AST))<br>Stay in recovery >24h
**Atrial natriuretic peptide**										
Shigemitsu 2015[47]	Retrospective cohort study	F/U not specified	16	Not specified	Tertiary referral hospital	Japan	Carperitide (no further details)	Standard care	MAP	Maternal mortality<br>Maternal side effects<br>Need for dialysis<br>Time to diuresis
**UTERINE CURETTAGE (8 studies)**										
Salvatore 1967[58]	Prospective cohort study	Enrolled immediately after birth<br>F/U 10 days	48	16-45	Tertiary referral hospital	Brazil††	Uterine curettage	No intervention	SBP + DBP	Maternal morbidity (development of pre-eclampsia with severe features – seizures)
Magann 1993[59]	RCT	Enrolled immediately after birth<br>F/U 24h (telephone at 7 weeks)	32	22.9±5.6<br>23.4±6.6	Tertiary referral hospital	USA	Uterine curettage	No intervention	MAP	Maternal side effects<br>Length of PN hospital stay<br>AHT requirement<br>Urine output<br>Laboratory values (HCT, plt, AST, LDH)
Magann 1994[60]	RCT	Enrolled immediately after birth<br>F/U 48h (telephone at 7 weeks)	45	22.3±6.4<br>22.8±6.6<br>22.8±6.1	Tertiary referral hospital	USA	Uterine curettage	(1) Nifedipine PO<br>OR<br>(2) Usual care	MAP	Maternal side effects<br>Urine output<br>Laboratory values (HCT, plt, AST, LDH)

For peer review only

For peer review only - http://bmjopen.bmj.com/site/about/guidelines.xhtml

Author and year	Methods		Participants				Intervention		Outcomes	
	Study design	*Duration*	*n*[*]	*Age /y*[†]	*Setting*	*Country*	*Intervention*	*Control(s)*	*Primary*	*Secondary*
Gocmen 1996[57]	Prospective cohort study	Enrolled immediately after birth F/U 24h	50	Not specified	Tertiary referral hospital	Turkey[†]	Uterine curettage	No intervention	MAP	Urine output<br>Laboratory values (plt)
Gomez 2005[61]	RCT	Enrolled immediately after birth F/U not specified	86	Not specified	Tertiary referral hospital	Peru	Uterine curettage	No intervention	MAP	Maternal side effects<br>Length of PN hospital stay<br>AHT requirement<br>Urine output
Alkan 2006[62]	RCT	Enrolled immediately after birth F/U 24h	56	22.8±3.4<br>24.6±7.5	Tertiary referral hospital	Turkey	Uterine curettage	No intervention	MAP	Maternal side effects<br>Urine output<br>Laboratory values (plt, LDH, AST, ALT)
Ragab 2013[15]	RCT	Enrolled immediately after birth F/U 96h	420	Not specified	Tertiary referral hospital	Egypt	Uterine curettage	No intervention	MAP	Maternal mortality<br>Maternal morbidity (development of pre-eclampsia with severe features)<br>MAP (time to MAP ≤105mmHg)<br>Urine output<br>Laboratory values (creatinine, plt, UA)
Mallapur 2015[18]	RCT	Enrolled immediately after birth F/U 7 days	100	Not specified	Tertiary referral hospital	India	Uterine curettage	No intervention	MAP	Length of PN hospital stay<br>Urine output<br>Laboratory values (plt, renal and liver function)
**ORGANISATION OF CARE (2 studies)**										
York 1997[26]	RCT	Enrolled immediately after birth F/U 8 weeks	96[§]	28±7<br>27±7	Tertiary referral	USA	Contact with nurse specialist; early discharge if criteria met; 2 scheduled home visits and 10 telephone calls (twice weekly for 2 weeks, then weekly for 6 weeks) during F/U	Standard care	Postnatal readmission to secondary care	Functional status<br>Patient satisfaction<br>Neonatal rehospitalisation / acute neonatal care<br>Cost
Bibbo 2014[33]	Retrospective cohort study	F/U not specified (but >7 days)	138	Not specified	Tertiary referral hospital	USA	Specialised postpartum clinic	Usual care	Postnatal readmission to secondary care and triage visits	Primary care provider F/U

[§] Mixture of hypertension and diabetes – unable to separate

For peer review only

For peer review only - http://bmjopen.bmj.com/site/about/guidelines.xhtml

APPENDIX S5: Summary of main results for included studies (n=39)

ANTIHYPERTENSIVE MEDICATIONS (18 studies)

Calcium channel blockers

BARTON 1990[32]

Population: Postnatal women with severe pre-eclampsia
Setting: Tertiary referral centres, USA
Intervention: Nifedipine 10mg PO 4-hourly for 48 hours
Comparison: Placebo

Primary outcome	Treatment effect	Number of participants	Quality of the evidence
MAP (18-24 hours after birth)	Nifedipine group 93.9±1.6mmHg, placebo group 100.2±2.6mmHg. Difference -6.3mmHg ($p<0.05$).	31 (16 intervention, 15 control); follow-up complete for all participants	Double-blind RCT; overall low risk of bias

VERMILLION 1999[21]

Population: Antenatal and postnatal women with severe pre-eclampsia or super-imposed pre-eclampsia
Setting: Tertiary referral centres (USA)
Intervention: Nifedipine 10mg stat PO then 20mg every 20 minutes until BP <160/110mmHg or max 5 doses (90mg) + IV placebo
Comparison: Labetalol 20mg, then 40mg, then 80mg IV every 20 minutes until BP <160/110mmHg or max 5 doses (300mg) + PO placebo

Primary outcome	Treatment effect	Number of participants	Quality of the evidence
SBP + DBP (time to target <160/100mmHg)	Nifedipine group 25.1±13.6 minutes, labetalol group 43.6±25.4 minutes. Difference -18.5 minutes ($p=0.002$).	50 (21 postnatal: 10 intervention, 11 control); follow-up complete for all participants	Double-blind RCT; overall high risk of bias (other bias); small number of postnatal women (42%) (n<30): unable to obtain data for postnatal subgroup

SAYIN 200534

Population: Postnatal women with pre-eclampsia, severe pre-eclampsia, superimposed pre-eclampsia or eclampsia
Setting: Tertiary referral centres (Turkey)
Intervention: Nifedipine 10mg PO 6-hourly until BP <150/100mmHg for 48 hours
Comparison: Methyldopa 250mg PO 8-hourly

Primary outcome	Treatment effect	Number of participants	Quality of the evidence
SBP + DBP (time to target <150/100mmHg)	Nifedipine group 6.7±2.5 days; methyldopa group 8.6±5.5 days. Difference -1.9 days (NS).	83 (42 intervention, 41 control); follow-up complete for all participants	Open-label RCT; overall high risk of bias (multiple domains)

Vasodilators

PALOT 1979[36]

Population: Postnatal women with 'arterial hypertensions of labour and the postpartum period'
Setting: Not specified (France)
Intervention: Hydralazine 5mg IV stat then 1% IV infusion, furosemide 20mg IV stat and 30% hypertonic glucose
Comparison: Clonidine IV and furosemide 20mg IV stat

Primary outcome	Treatment effect	Number of participants	Quality of the evidence
Maternal morbidity (development of pre-eclampsia with severe features)	Hydralazine group: no women developed eclampsia, clonidine group: 2 women developed eclampsia. No statistical analysis.	54 (11 intervention, 24 control, 19 non-systematic treatment); completeness of follow-up not specified	Retrospective cohort study; overall high risk of bias (comparability); no statistical analysis

GRIFFIS 1989[38 39]

Population: Postnatal women with pre-eclampsia
Setting: Tertiary referral centres (USA)
Intervention: Hydralazine 20mg IM 6-hourly for 24h
Comparison: Methyldopa 250mg IV 6-hourly for 24h

Primary outcome	Treatment effect	Number of participants	Quality of the evidence
MAP (mean at 6 and 12 hours)	6 hours: hydralazine group 104.5mmHg, methyldopa group 112mmHg. Difference -7.5mmHg ($p=0.0057$). 12 hours: hydralazine group 100mmHg, methyldopa group108mmHg. Difference -8mmHg (NS).	26 (12 intervention, 14 control); follow-up complete for all participants	Open-label RC; overall high risk of bias (multiple domains); small sample size (n<30)

WALSS RODRIGUEZ 1991[40]

Population: Postnatal women with severe pre-eclampsia
Setting: Not specified (Mexico)
Intervention: Hydralazine 40mg PO 6-hourly, duration not specified + if DBP >110mmHg PRN nifedipine 10mg sublingual every 30 minutes, to maximum of 3 doses (30mg)
Comparison: Nifedipine 10mg sublingual every 30 minutes if DBP ≥110mmHg

For peer review only

For peer review only - http://bmjopen.bmj.com/site/about/guidelines.xhtml

Primary outcome	Treatment effect	Number of participants	Quality of the evidence
SBP (mean)	Hydralazine group 143.6mmHg, nifedipine group138.0mmHg. Difference 5.6mmHg (NS).	38 (18 intervention, 20 control); completeness of follow-up not specified	Open-label RCT; overall high risk of bias (multiple domains)

BEGUM 2002[17]

Population: Antenatal and postnatal women with eclampsia
Setting: Tertiary referral centres (Bangladesh)
Intervention: Hydralazine 5mg then 2mg IV bolus every 15 minutes until DBP 90-95mmHg
Comparison: Hydralazine 20mg /200ml normal saline IV infusion; 10 drops per min, increased by 5 drops at 15 min intervals; until DBP 90-95mmHg

Primary outcome	Treatment effect	Number of participants	Quality of the evidence
DBP (time to target 90-95mmHg)	Bolus hydralazine group 65.23±23.38 minutes, hydralazine infusion group 186.36±79.77 minutes. Difference -121.13 minutes (p<0.001).	77 (15 postnatal: 9 intervention, 6 control); completeness of follow-up not specified	Open-label RCT; overall high risk of bias (multiple domains); small number of postnatal women (19%) (n<30): unable to obtain data for postnatal subgroup

VIGIL DE GRACIA 2007[35]

Population: Postnatal women with severe gestational hypertension, severe pre-eclampsia or super-imposed pre-eclampsia
Setting: Tertiary referral centres (Panama)
Intervention: Hydralazine 5mg IV every 20 minutes until BP <160/110mmHg or maximum 5 doses
Comparison: Labetalol 20mg, then 40mg, then 80mg IV every 20 minutes until BP <160/110mmHg or maximum 5 doses (300mg)

Primary outcome	Treatment effect	Number of participants	Quality of the evidence
SBP + DBP (persistent hypertension >=160/110mmHg after 5 doses of medication)	Hydralazine group 0/42, labetalol group 1/40 (NS).	82 (42 intervention, 40 control); follow-up complete for all participants	Open-label RCT; overall high risk of bias (multiple domains)

HENNESSY 2007[23]

Population: Antenatal and postnatal women with pre-eclampsia, superimposed pre-eclampsia or essential hypertension
Setting: Tertiary referral (Australia)
Intervention: Diazoxide 15mg IV every 3 minutes, until target BP (140/90mmHg) reached or maximum cumulative dose 300mg
Comparison: Hydralazine 5mg IV every 20 minutes, until target BP (140/90mmHg) reached or maximum cumulative dose 15mg

Primary outcome	Treatment effect	Number of participants	Quality of the evidence
SBP + DBP (proportion achieving target BP <=140/90mmHg)	Diazoxide group 67%, hydralazine group 43% (p<0.01). RR 0.637 (95% CI 0.46 to 0.89) for not reaching target BP with intervention.	124 total (37 postnatal: 11 intervention, 16 control); follow-up complete for all participants	Open-label RCT; overall high risk of bias (multiple domains); small proportion of postnatal women (30%): unable to obtain data for postnatal subgroup

Beta-blockers

GARDEN 1982[24]

Population: Antenatal and postnatal women with severe pre-eclampsia or eclampsia
Setting: Tertiary referral (South Africa)
Intervention: Labetalol 200mg/200ml 5% dextrose, 20mg/h IV infusion, doubled every 30 minutes until DBP <100mmHg or maximum dose 160mg/hour
Comparison: Dihydralazine 100mg/200ml 5% dextrose, 10mg/h IV infusion, doubled every 30 minutes until DBP <100mmHg or maximum dose 80mg/hour

Primary outcome	Treatment effect	Number of participants	Quality of the evidence
DBP (proportion achieving target DBP 90-100mHg within 2 hours)	Labetalol group 5/6, dihydralazine group 2/6. No statistical analysis.	12 total (6 postnatal: 3 intervention, 3 control); follow-up complete for all participants	RCT (blinding not specified); overall high risk of bias (other bias); very small sample size (n<15): unable to obtain data for postnatal subgroup

FIDLER 1982[42]

Population: Postnatal women with gestational hypertension
Setting: Tertiary referral (UK)
Intervention: Timolol 5mg PO 8-hourly for 9 days
Comparison: Methyldopa 250mg PO 8-hourly for 9 days

Primary outcome	Treatment effect	Number of participants	Quality of the evidence
DBP (day 1)	Timolol group 88.7mmHg, methyldopa group 93.8mmHg. Difference -5.1mmHg (p<0.05).	80 (40 intervention, 40 control); follow-up complete in 79/80 (99%)	RCT (blinding not specified); overall high risk of bias (multiple domains)

MABIE 1987[22]

Population: Antenatal and postnatal women with pre-eclampsia, superimposed pre-eclampsia, eclampsia or essential hypertension
Setting: Tertiary referral (USA)
Intervention: Labetalol 20mg IV every 10 minutes then escalating until DBP <100mmHg or maximum cumulative dose reached (300mg)
Comparison: Hydralazine 5mg IV every 10 minutes until DBP <100mmHg

For peer review only

Primary outcome	Treatment effect	Number of participants	Quality of the evidence
MAP (mean maximal decrease)	Labetalol group 25.5±11.2mmHg, hydralazine group 33.3±13.2mmHg. Difference -7.8mmHg (*p*=0.02).	60 (41 postnatal: 27 intervention, 14 control); follow-up complete for all participants	Open-label RCT; overall high risk of bias (multiple domains)

SHUMARD 2016[41]
Population: Postnatal women with gestational hypertension or pre-eclampsia
Setting: Not specified (USA)
Intervention: Labetalol PO (variable dose and frequency)
Comparison: Nifedipine PO (variable dose and frequency)

Primary outcome	Treatment effect	Number of participants	Quality of the evidence
Length of hospital stay after delivery	Labetalol group 3.5 days, nifedipine group 3.6 days. Difference -0.1 days (NS).	128 (42 intervention, 86 control); follow-up complete for all participants	Retrospective cohort study; overall high risk of bias (comparability); conference abstract only, authors did not provide further data

SHARMA 2017[27,28]
Population: Postnatal women with gestational hypertension or pre-eclampsia
Setting: Tertiary referral (USA)
Intervention: Labetalol 200mg PO 12-hourly
Comparison: Nifedipine XL 30mg PO once daily

Primary outcome	Treatment effect	Number of participants	Quality of the evidence
SBP + DBP (time to sustained BP control: absence of severe hypertension for >=12 hours)	Labetalol group 37.6 hours, nifedipine group 38.2 hours. Difference -0.6 hours (NS).	50 (25 intervention, 25 control); follow-up complete for all participants	Open-label RCT; overall high risk of bias (multiple domains)

Other antihypertensive medications

GAISIN 2013[25]
Population: Postnatal women with pre-eclampsia, super-imposed pre-eclampsia or essential hypertension
Setting: Not specified (Russia)
Intervention: Indapamide 1.5mg PO OD, duration unclear
Comparison: Adjusted dose methyldopa

Primary outcome	Treatment effect	Number of participants	Quality of the evidence
SBP + DBP	Indapamide group 113±6/74±4mmHg, methyldopa group 116±5/75±4mmHg. Difference -3/+1mmHg (NS).	30 (15 intervention, 15 control); completeness of follow-up not specified	Open-label RCT; overall high risk of bias (multiple domains); conference abstract only, authors did not provide further data

GAISIN 2014[37]
Population: Postnatal women with pre-eclampsia
Setting: Not specified (Russia)
Intervention: Indapamide 1.5mg PO once daily + ursodeoxycholic acid 250mg PO three times daily, duration unclear
Comparison: Adjusted dose methyldopa

Primary outcome	Treatment effect	Number of participants	Quality of the evidence
SBP + DBP	Indapamide group 122±6/75±4 mmHg, methyldopa group 126±6/78±5mmHg. Difference -4/-3mmHg (NS).	30 (allocation not described); completeness of follow-up not specified	Open-label RCT; overall high risk of bias (multiple domains); conference abstract only, authors did not provide further data; number of participants in each group not stated

KREBS 1956[43,44]
Population: Postnatal women with gestational hypertension, pre-eclampsia, severe pre-eclampsia or eclampsia
Setting: Not specified (Germany)
Intervention: Reserpine 0.25mg PO or intramuscular 6-8 hourly for 7 days
Comparison: Phenobarbital

Primary outcome	Treatment effect	Number of participants	Quality of the evidence
SBP + DBP (maximal reduction)	Reserpine halved time to maximal BP reduction (no further details reported). No statistical analysis.	140 (70 intervention, 70 control); completeness of follow-up not specified	Retrospective cohort study; overall high risk of bias (selection and outcome assessment); no statistical analysis

NORONHA NETO 2016[29-31]
Population: Postnatal women with severe HDP
Setting: Tertiary referral (Brazil)
Intervention: Clonidine 0.1mg PO repeated every 20 minutes to max 6 doses
Comparison: Captopril 25mg PO repeated every 20 minutes to max 6 doses

For peer review only

Primary outcome	Treatment effect	Number of participants	Quality of the evidence
SBP + DBP (episodes SBP ≥180mmHg and/or DBP ≥110mmHg)	Clonidine group 2.1±2.1 episodes, captopril group 3.5±4.7 episodes. Difference -1.4 episodes (NS).	90 (45 intervention, 45 control); completeness of follow-up not specified	Double-blind RCT; overall low risk of bias
**DIURETICS (4 studies)**			
**MATTHEWS 1997[46]**<br>**Population:** Postnatal women with severe pre-eclampsia or eclampsia<br>**Setting:** Tertiary referral centres (UK)<br>**Intervention:** Furosemide 40mg PO once daily for 7 days<br>**Comparison:** Placebo			
**Primary outcome**	**Treatment effect**	**Number of participants**	**Quality of the evidence**
MAP (decrease)	Intervention group -10.6mmHg, control group -9.75mmHg. Difference -0.85mmHg (NS).	19 (10 intervention, 9 control); follow-up complete in 18/19 (95%)	Double-blind RCT; overall high risk of bias (other bias); small sample size (n<30)
**ASCARELLI 2005[16]**<br>**Population:** Postnatal women with pre-eclampsia, severe pre-eclampsia or superimposed pre-eclampsia<br>**Setting:** Tertiary referral centres (USA)<br>**Intervention:** Furosemide 20mg PO once daily + potassium 20mEq PO once daily for 5 days<br>**Comparison:** No intervention			
**Primary outcome**	**Treatment effect**	**Number of participants**	**Quality of the evidence**
SBP	No significant difference between groups (details not reported). Severe pre-eclampsia (n=70) day 2 SBP furosemide group 142±13mmHg, usual care group 153±19mmHg. Difference -11mmHg (*p*<0.004).	264 (132 intervention, 132 control); completeness of follow-up not specified.	Open-label RCT; overall high risk of bias (multiple domains)
**AMORIM 2015[45]**<br>**Population:** Postnatal women with severe pre-eclampsia<br>**Setting:** Tertiary referral (Brazil)<br>**Intervention:** Furosemide 40mg PO once daily for maximum 5 days<br>**Comparison:** Placebo			
**Primary outcome**	**Treatment effect**	**Number of participants**	**Quality of the evidence**
SBP + DBP	Furosemide group had significantly improved SBP + DBP. Magnitude of difference not reported (*p*<0.001).	120 (allocation not described); follow-up complete in 118/120 (98%).	Double-blind RCT; overall high risk of bias (reporting bias); conference abstract only, authors did not provide further data; number of participants in each group not stated
**VEENA 2017[19]**<br>**Population:** Postnatal women with severe pre-eclampsia<br>**Setting:** Tertiary referral centre (India)<br>**Intervention:** Furosemide 10mg PO once daily plus nifedipine 10mg PO three times daily for 3 days<br>**Comparison:** Nifedipine 10mg PO three times daily for 3 days			
**Primary outcome**	**Treatment effect**	**Number of participants**	**Quality of the evidence**
SBP + DBP	No significant difference between groups (absolute values and differences not reported, *p*=0.457 for SBP and *p*=0.642 for DBP).	100 (50 intervention, 50 control); follow-up complete in 98/100 (98%)	Open-label RCT; overall high risk of bias (multiple domains)
**OTHER DRUGS (7 studies)**			
**Selective 5-HT antagonists**			
**WEINER 1982[48]**<br>**Population:** Postnatal women with severe pre-eclampsia<br>**Setting:** Tertiary referral (USA)<br>**Intervention:** R41468 IV (dose not specified) bolus then infusion for 90 minutes<br>**Comparison:** Placebo			
**Primary outcome**	**Treatment effect**	**Number of participants**	**Quality of the evidence**
MAP (mean maximal decline)	SSRI group -31.6mmHg, placebo group -6.0mmHg. Difference -25.6mmHg (p<0.001).	5 (crossover); follow-up complete in all participants	Double blind RCT (crossover); overall high risk of bias (other bias); conference abstract only, authors did not provide further data; very small sample size (n<15)

For peer review only

WEINER 1984[49]

Population: Postnatal women with pre-eclampsia and super-imposed pre-eclampsia
Setting: Tertiary referral (USA)
Intervention: Ketanserin 10mg IV bolus then 4mg/hr IV infusion. Repeat bolus after 5 minutes if no response.
Comparison: Placebo

Primary outcome	Treatment effect	Number of participants	Quality of the evidence
SBP + DBP (mean maximal decline)	SSRI group -41/-34mmHg, placebo group -7/-7mmHg. Difference -34/-27mmHg (p<0.001).	20 (crossover); follow-up complete in all participants	Double blind RCT (crossover); overall high risk of bias (other bias); small sample size (n<30)

MONTENEGRO 1985[50]

Population: Postnatal women with pre-eclampsia
Setting: Tertiary referral (USA)
Intervention: Ketanserin 10mg IV bolus, repeated if no response. If no response to second bolus IV infusion 4mg/hr (increments of 2mg/hr every 10 minutes to max 12mg/hr).
Comparison: Placebo

Primary outcome	Treatment effect	Number of participants	Quality of the evidence
MAP	SSRI group had significantly improved MAP, over 30 minutes after drug administered. F = 9.66 (p<0.01)	30 (crossover); follow-up complete in 23/30 (77%)	Double blind RCT (crossover); overall high risk of bias (multiple domains)

Alternative therapies

HLADUNEWICH 2006[51]

Population: Postnatal women with pre-eclampsia
Setting: Tertiary referral (USA)
Intervention: L-arginine 3.5g PO four times daily OR L-arginine 10g IV three times daily (if unable to take PO) for 3-9 days postpartum
Comparison: Placebo

Primary outcome	Treatment effect	Number of participants	Quality of the evidence
MAP	Day 3: L-arginine group 102±12mmHg, placebo group 103±12mmHg. Difference -1mmHg (NS). Day 10: L-arginine group 98±14mmHg, placebo group 96±1mmHg. Difference 2mmHg (NS).	45 (22 intervention, 23 control); follow-up complete in 39/45 (87%)	Double blind RCT; overall high risk of bias (multiple domains)

LIU 2009[52]

Population: Postnatal women with severe pre-eclampsia
Setting: District general (China)
Intervention: Shengkangbao 10g PO or IV twice daily for 3 weeks
Comparison: No intervention

Primary outcome	Treatment effect	Number of participants	Quality of the evidence
Percentage of cases with positive albuminuria	3 weeks: shengkangbao group 0.7+/-0.8% positive albuminuria, usual care group 1.5+/-0.9%. Difference -0.8% (p<0.01).	77 (allocation not described); follow-up complete in 72/77 (94%)	Open-label quasi-randomised study; overall high risk of bias (multiple domains)

Steroids

BARRILLEAUX 2005[53 54]

Population: Postnatal women with severe pre-eclampsia and eclampsia
Setting: Tertiary referral (USA)
Intervention: Dexamethasone 10mg x2, then 5mg x 2 IV 12-hourly for 48 hours
Comparison: Placebo (IV saline)

Primary outcome	Treatment effect	Number of participants	Quality of the evidence
Antihypertensive medication requirement	Dexamethasone group 38/77 (49%), placebo group 31/80 (39%) required antihypertensive treatment in the first 48h PN. Difference 10% (NS).	157 (77 intervention, 80 control); follow-up complete in 155/157 (99%)	Double blind RCT; overall high risk of bias (reporting bias)

Atrial natriuretic peptide

SHIGEMITSU 2015[47]

Population: Postnatal women with severe pre-eclampsia, HELLP syndrome or placental abruption
Setting: Tertiary referral (Japan)
Intervention: Carperitide (no further details supplied)
Comparison: No intervention

Primary outcome	Treatment effect	Number of participants	Quality of the evidence
MAP	Carperitide group had significantly improved MAP at 48 hours. Magnitude of difference not reported, no p value presented.	16 (6 intervention, 10 control); follow-up complete for all participants	Retrospective cohort study; overall high risk of bias (comparability); conference abstract only, authors did not provide further data; small sample size (n<30)

For peer review only

For peer review only - http://bmjopen.bmj.com/site/about/guidelines.xhtml

UTERINE CURETTAGE (8 studies)

SALVATORE 1967[58]
Population: Postnatal women with severe pre-eclampsia or eclampsia
Setting: Tertiary referral (Brazil)
Intervention: Uterine curettage
Comparison: No intervention

Primary outcome	Treatment effect	Number of participants	Quality of the evidence
SBP + DBP (proportion achieving target <140/90mmHg)	24 hours: curettage group 45%, usual care group 11%. No statistical analysis. 48 hours: curettage group 70%, usual care group 29%. No statistical analysis.	48 (20 intervention, 28 control; follow-up complete for all participants	Prospective cohort study; overall high risk of bias (comparability); significant differences in study groups (9/20 intervention group eclamptic at enrolment, 28/28 control group)

MAGANN 1993[59]
Population: Postnatal women with severe pre-eclampsia
Setting: Tertiary referral (USA)
Intervention: Uterine curettage
Comparison: No intervention

Primary outcome	Treatment effect	Number of participants	Quality of the evidence
MAP	Curettage group had significantly improved MAP to 24 hours after birth. Difference -6 to -10mmHg (16 hours p<0.0002).	32 (16 intervention, 16 control); completeness of follow-up not specified	Open-label RCT; overall high risk of bias (multiple domains)

MAGANN 1994[60]
Population: Postnatal women with severe pre-eclampsia
Setting: Tertiary referral (USA)
Intervention: Uterine curettage
Comparison: Oral nifedipine OR no intervention

Primary outcome	Treatment effect	Number of participants	Quality of the evidence
MAP	Curettage group had significantly improved MAP 8-48 hours after birth. Difference -9 to -13mmHg (p=0.0017). No difference between curettage and nifedipine.	45 (15 intervention, 15 each control group); completeness of follow-up not specified	Open-label RCT; overall high risk of bias (multiple domains)

GOCMEN 1996[57]
Population: Postnatal women with pre-eclampsia
Setting: Tertiary referral (Turkey)
Intervention: Uterine curettage
Comparison: No intervention

Primary outcome	Treatment effect	Number of participants	Quality of the evidence
MAP	Curettage group had significantly improved MAP to 24 hours after birth. Magnitude of difference not reported (p=0.01).	50 (30 intervention, 20 control); completeness of follow-up not specified	Prospective cohort study; overall high risk of bias (comparability and outcome assessment); conference abstract only, authors did not provide further data

GOMEZ 2005[61]
Population: Postnatal women with severe pre-eclampsia
Setting: Tertiary referral (Peru)
Intervention: Uterine curettage
Comparison: No intervention

Primary outcome	Treatment effect	Number of participants	Quality of the evidence
MAP	Intervention group had significantly improved MAP. Time point not specified. Magnitude of difference not reported (p<0.001).	86 (27 intervention, 59 control); completeness of follow-up not specified	Open-label RCT; overall high risk of bias (multiple domains); conference abstract only, authors did not provide further data

ALKAN 2006[62]
Population: Postnatal women with severe pre-eclampsia
Setting: Tertiary referral (Turkey)
Intervention: Uterine curettage
Comparison: No intervention

Primary outcome	Treatment effect	Number of participants	Quality of the evidence
MAP	24 hours: curettage group 103.4±7.8mmHg, usual care group 110.2±4.8. Difference -6.8mmHg (p<0.05).	56 (31 intervention, 25 control); follow-up complete for all participant	Open-label RCT; overall high risk of bias (multiple domains)

For peer review only

RAGAB 2013[15]

Population: Postnatal women with severe pre-eclampsia or eclampsia
Setting: Tertiary referral (Egypt)
Intervention: Uterine curettage
Comparison: No intervention

Primary outcome	Treatment effect	Number of participants	Quality of the evidence
MAP	6 hours: curettage group 140.1±6.12mmHg, usual care group 152.4±3.7mmHg. Difference -12.3mmHg (*p*=0.02). 24 hours: curettage group 101.4±7.14mmHg, usual care group 110.6±2.22mmHg. Difference -9.2mmHg (*p*=0.01).	420 (220 intervention, 200 control); follow-up complete for all participants	Open-label RCT; overall high risk of bias (multiple domains)

MALLAPUR 2015[18]

Population: Postnatal women with severe pre-eclampsia or eclampsia
Setting: Tertiary referral (India)
Intervention: Uterine curettage
Comparison: No intervention

Primary outcome	Treatment effect	Number of participants	Quality of the evidence
MAP	From 4 hours after birth: curettage group 116±4.4mmHg, usual care group 123.6±6.1mmHg. Difference -7.6mmHg (*p*<0.001).	100 (50 intervention, 50 control); completeness of follow-up not specified	Open-label RCT; overall high risk of bias (multiple domains); conference abstract only, authors did not provide further data

ORGANISATION OF CARE (2 studies)

YORK 1997[26]

Population: Postnatal women with pre-eclampsia or essential hypertension, or diabetes
Setting: Tertiary referral (USA)
Intervention: Nurse specialist follow-up
Comparison: No intervention

Primary outcome	Treatment effect	Number of participants	Quality of the evidence
Postnatal readmission to secondary care	No significant difference between groups.	96 (44 intervention, 52 control); completeness of follow-up not specified	Open-label RCT; overall high risk of bias (multiple domains); population mixed diabetes and/or hypertension – unable to separate

BIBBO 2014[33]

Population: Postnatal women with pre-eclampsia
Setting: Tertiary referral (USA)
Intervention: Specialised postpartum clinic
Comparison: No intervention

Primary outcome	Treatment effect	Number of participants	Quality of the evidence
Postnatal readmission to secondary care and triage visits	Clinic group 21.7%, usual care group 8.7%. Difference 13% (*p*<0.039).	138 (69 intervention, 69 control); completeness of follow-up not specified.	Retrospective cohort study; overall high risk of bias (comparability); conference abstract only, authors did not provide further data

For peer review only

Appendix S6: Risk of bias in included studies (n=38)

Appendix S6a: Risk of bias in included RCTs and quasi-randomised studies (n=31)

Study ID	Random sequence generation	Allocation concealment	Blinding of participants and personnel	Blinding of outcome assessment	Incomplete outcome data	Selective reporting	Other bias
**ANTIHYPERTENSIVE MEDICATIONS**							
Fidler 1982[42]	Unclear	Unclear	Unclear	Unclear	High	High	Low
Garden 1982[24]	Unclear	Unclear	Unclear	Unclear	Low	Unclear	High
Mabie 1987[22]	Unclear	Unclear	High	High	Low	Low	High
Griffis 1989[38 39]	Unclear	Low	High	High	High	High	High
Barton 1990[32]	Low	Low	Low	Low	Low	Low	Low
Walss Rodriguez 1991[40]	Low	Low	High	High	Unclear	Unclear	Low
Vermillion 1999[21]	Low	Low	Low	Low	Low	Low	High
Begum 2002[17]	High	High	High	High	Unclear	Unclear	High
Sayin 2005[34]	Unclear	Unclear	High	High	Low	Unclear	High
Hennessy 2007[23]	Unclear	Low	High	High	Low	Low	High
Vigil-de-Gracia 2007[35]	Low	Low	High	High	Low	Low	Low
Gaisin 2013[25]	Unclear	Unclear	High	High	Unclear	High	High
Gaisin 2014[37]	Unclear	Unclear	High	High	Unclear	Unclear	High
Noronha Neto 2016[29-31]	Low	Low	Low	Unclear	Low	Low	Low
Sharma 2017[27 28]	Low	Low	High	High	Unclear	Low	Low
**DIURETICS**							
Matthews 1997[46]	Unclear	Low	Low	Low	Low	Unclear	High
Ascarelli 2005[16]	Unclear	Low	High	High	Unclear	High	Low
Amorim 2015[45]	Low	Low	Low	Low	Low	High	Low
Veena 2017[19]	Low	Low	High	High	Unclear	Unclear	Unclear
**OTHER DRUGS**							
Weiner 1982[48]	Unclear	Unclear	Low	Low	Unclear	Unclear	High
Weiner 1984[49]	Low	Unclear	Low	Low	Low	Unclear	High
Montenegro 1985[50]	Unclear	Unclear	Low	Low	High	High	High
Barrilleaux 2005[53 54]	Low	Low	Low	Low	Low	High	High
Hladunewich 2006[51]	Low	Low	Low	Low	High	High	High
Liu 2009[52]	High	High	High	High	High	Unclear	High
**UTERINE CURETTAGE**							
Magann 1993[59]	Low	Low	High	High	Unclear	Unclear	Low
Magann 1994[60]	Low	Unclear	High	High	Unclear	Unclear	Low
Gomez 2005[61]	Unclear	Unclear	High	High	Unclear	High	Low
Alkan 2006[62]	Unclear	Unclear	High	High	Low	High	High
Ragab 2013[15]	Low	Low	High	High	Low	Low	Low
Mallapur 2015[18]	Low	Unclear	High	High	Unclear	Unclear	High
**ORGANISATION OF CARE**							
York 1997[26]	Unclear	Low	High	High	Unclear	Unclear	High

For peer review only

Appendix S6b: Risk of bias in included cohort studies (n=7)

Study ID	Selection: Representative-ness[2]	Selection: Selection of non-exposed[3]	Selection: Ascertainment of exposure[4]	Selection: Outcome of interest not present at start	Comparability[1]	Outcome: Assessment[5]	Outcome: F/U long enough	Outcome: Adequacy of F/U[6]
**ANTIHYPERTENSIVE MEDICATIONS**								
Krebs 1956[43 44]	Low (a)	Low (a)	Unclear (d)	Low (Yes)	Low (a)	High (b)	Low (Yes)	Unclear (d)
Palot 1979[36]	Unclear (d)	Low (a)	Unclear (d)	Low (Yes)	High (Neither)	Unclear (d)	Low (Yes)	Unclear (d)
Shumard 2016[41]	Low (a)	Low (a)	Low (a)	Low (Yes)	High (Neither)	Low (a)	Low (Yes)	Low (a)
**OTHER DRUGS**								
Shigemitsu 2015[47]	Unclear (d)	Unclear (c)	Low (a)	Low (Yes)	High (Neither)	Low (a)	Low (Yes)	Unclear (d)
**UTERINE CURETTAGE**								
Salvatore 1967[58]	High (b)	High (b)	Low (a)	Low (Yes)	High (Neither)	Low (a)	Low (Yes)	Low (a)
Gocmen 1996[57]	Unclear (d)	Unclear (c)	Unclear (d)	Low (Yes)	High (Neither)	Unclear (d)	High (No)	Unclear (d)
**ORGANISATION OF CARE**								
Bibbo 2014[33]	Unclear (d)	Unclear (c)	Unclear (d)	Low (Yes)	High (Neither)	Unclear (d)	Low (Yes)	Unclear (d)

[1] (a) study controls for most important factor; (b) study controls for any additional factor

[2] (a) truly representative of the average in the community; (b) somewhat representative of the average in the community; (c) selected group of users e.g. nurses, volunteers; (d) no description of the derivation of the cohort

[3] (a) drawn from the same community as the exposed cohort; (b) drawn from a different source; (c) no description of the derivation of the non-exposed cohort

[4] (a) secure record (e.g. surgical record); (b) structured interview; (c) written self-report; (d) no description

[5] (a) independent blind assessment; (b) record linkage; (c) self-report; (d) no description

[6] (a) complete follow-up; (b) subjects lost to follow-up unlikely to introduce bias (>90% follow-up rate); (c) follow up rate <90% and no description of those lost; (d) no statement

For peer review only
